# Populations at Risk for Severe or Complicated Rhinovirus Illness: A Systematic Review and Meta‐Analysis

**DOI:** 10.1111/irv.70251

**Published:** 2026-03-18

**Authors:** David Gou, Jessica Bartoszko, Laura Weiler, Asal Alavi Parsi, Ante Cuvalo, Sahith Rajkumar, Dominik Mertz, Mark Loeb

**Affiliations:** ^1^ Temerty Faculty of Medicine University of Toronto Toronto Ontario Canada; ^2^ Faculty of Health Sciences McMaster University Hamilton Ontario Canada; ^3^ Department of Health Research Methods, Evidence and Impact McMaster University Hamilton Canada; ^4^ Faculty of Engineering McMaster University Hamilton Ontario Canada; ^5^ Division of Infectious Diseases, Department of Medicine McMaster University Hamilton Ontario Canada; ^6^ Division of Microbiology, Department of Pathology and Laboratory Medicine McMaster University Hamilton Ontario Canada

**Keywords:** meta‐analysis, respiratory tract infections, rhinovirus, risk, systematic review

## Abstract

**Background:**

Although most rhinovirus infections are mild and subside quickly, vulnerable populations may experience severe illness. Identifying populations at risk for severe or complicated rhinovirus illness can strengthen the ongoing search for preventative and therapeutic treatments. This systematic review and meta‐analysis aimed to summarize the populations at risk for the development of severe or complicated rhinovirus illness.

**Methods:**

We searched CENTRAL, EMBASE, and MEDLINE in April 2024 for studies reporting risk factors for severe rhinovirus infection, defined as lower respiratory tract infection (LRTI), hospitalization, critical care unit (CCU) admission, mechanical ventilation, or death. We pooled odds ratios using random‐effects meta‐analysis, assessed risk of bias using the Newcastle–Ottawa Scale, and rated the certainty of evidence using the GRADE framework.

**Results:**

From 29 observational studies (*n* = 13,185 participants), we analyzed 13 risk factor–outcome combinations. With high certainty, age < 1 year and premature birth are not associated with the risk of LRTI, and diabetes mellitus is not associated with mortality. With moderate certainty, any comorbidity and pulmonary comorbidity are probably associated with increased risk of LRTI, age > 18 years and malignancy are probably associated with increased risk of mortality, and malignancy is probably associated with an increased risk of CCU admission. Many risk factors lacked sufficient evidence for meta‐analysis.

**Conclusions:**

Individuals with comorbidities are at greater risk of severe rhinovirus illness. Our findings can inform clinical risk stratification and guide the development and targeted use of emerging therapies. Further comprehensive research is required to elucidate additional risk factors and strengthen the evidence.

## 1 Introduction

Rhinovirus is a common cause of acute respiratory tract illnesses and upper respiratory tract infections [1]. Rhinovirus has been detected in nearly 25% of children admitted to hospital and pediatric intensive care units [2, 3]. There is a significant societal impact of rhinovirus infections owing to disruptions in social interactions, decreases in productivity, and absenteeism from work or school [4].

While primarily associated with the common cold, rhinovirus infection presentation can range from asymptomatic to severe lower respiratory tract infections (LRTIs) and other severe outcomes [5, 6, 7]. Such severe or complicated courses burden healthcare systems. In the United States, the annual incidence of rhinovirus‐associated hospitalizations in adults is estimated to range from 137 to 174 per 100,000 people [8]. Further complications may require admission to a critical care unit (CCU) or the use of mechanical ventilation and may result in death [9]. Identifying those at risk for severe illness may allow for preventative measures and can reduce unnecessary diagnostic tests or treatments [10].

Currently, there are no specific preventative or therapeutic treatments for rhinovirus infection [11]. Historically, candidate antiviral drugs that have been assessed were either ineffective or not commercially viable, and vaccines to date have been ineffective due to antigenic diversity [11]. Nevertheless, new treatments have shown some promise [12, 13, 14, 15]. It is therefore important to identify specific populations at risk for complications of rhinovirus infection that could benefit most from such therapies.

Some studies have explored the roles of age, comorbidities, and lifestyle factors in rhinovirus‐associated hospitalizations and LRTIs [16, 17]. However, existing evidence is fragmented, with studies reporting on different types of risk factors, varying severe outcomes, and heterogeneous populations. Thus, this systematic review and meta‐analysis sought to comprehensively summarize and quantify the factors associated with severe or complicated rhinovirus illness.

## 2 Methods

This systematic review and meta‐analysis was conducted with guidance from the Cochrane Handbook, and its findings are reported in accordance with the Preferred Reporting Items for Systematic Reviews of Interventions (PRISMA) 2020 statement [18, 19]. The completed PRISMA 2020 checklist is found in Table S1. Our decisions regarding search strategy, inclusion criteria, study selection, data extraction, risk of bias assessment, and data analysis were all established a priori, although the protocol was not registered publicly.

### 2.1 Study Identification and Data Extraction

We systematically searched CENTRAL, EMBASE (Ovid), and MEDLINE (Ovid) from inception to April 4, 2024 using a combination of relevant keywords and subject headings. The complete search strategies used for each database are presented in Tables S2–S4.

We included cohort, case‐control, cross‐sectional, and ecological studies, as well as baseline data from randomized controlled trials, which reported original data on a population with laboratory‐confirmed rhinovirus infection (by viral culture, nucleic acid amplification testing, antigen detection, or paired serology testing). Relevant outcomes representing severe or complicated rhinovirus illness were LRTI, community‐acquired pneumonia, all‐cause hospitalization, rhinovirus‐related hospitalization, admission to a CCU, mechanical ventilation, death, and rhinovirus‐related death. Eligible studies reported on any patient‐related risk factor associated with at least one outcome of interest or a composite thereof. We excluded noncomparative studies, conference abstracts, and non‐English studies.

Five reviewers (DG, LW, AAP, AC, and SR) screened titles and abstracts independently and in duplicate using COVIDENCE [20]. Any disagreements were resolved by discussion and consultation with a third reviewer if necessary to achieve consensus. Next, we retrieved all eligible records for inclusion and conducted independent and in‐duplicate full‐text review. Disagreements were resolved in a similar manner to reach consensus.

Four reviewers (DG, LW, AAP, and AC) extracted data independently and in duplicate using standardized and prepiloted extraction sheets. We abstracted information related to study design and study participants. In addition, we extracted relevant data for each risk factor, including its definition, frequency, measure of effect, and confidence interval (CI) for the associated outcome. We prioritized the extraction of measures of association (ORs, HRs, or RRs) over proportions. If a study reported on the same risk factor–outcome pair using multiple approaches, we prioritized adjusted ORs, HRs, or RRs, followed by crude ORs, HRs, or RRs. Discrepancies were resolved through discussion, with consultation of a third reviewer if necessary.

### 2.2 Data Synthesis and Data Analysis

We classified risk factors amenable to meta‐analysis into comorbidities and demographic characteristics. Assuming that heterogeneity existed in findings between studies, we employed random effects meta‐analysis to pool effect estimates and 95% CIs for each risk factor through inverse variance analysis when possible.

We assessed statistical heterogeneity through visual inspection of forest plots for the consistency of point estimates and the overlap of CIs and the *I* ^2^ statistic. We assessed publication bias through visual inspection of funnel plots when at least 10 studies were included in the meta‐analysis.

For outcomes reported as proportions, we calculated odds ratios using the R *epitools* package. We used the R *metafor* and *epitools* packages for all statistical analyses, with a two‐sided *p*‐value of 0.05 or less indicating statistical significance for all comparisons.

Where possible, we planned to explore age (children vs. adults) as a source of heterogeneity in subgroup analyses. Children were defined as persons aged 17 years, while adults were those 18 years and older.

### 2.3 Risk of Bias Assessment and Certainty of Evidence

Two reviewers (DM and ML) used the Newcastle–Ottawa Scale (NOS) to assess the risk of bias of the included observational studies independently and in duplicate [21]. NOS assesses risk of bias by allocating up to nine points across three domains: selection of the study population (four points), comparability of cases and controls (two points), and ascertainment of exposure and outcome (three points). A higher score indicates a lower risk of bias.

We assessed the certainty of evidence relating to each risk factor using the Grading of Recommendations Assessment, Development, and Evaluation (GRADE) framework [22]. Our application of the GRADE framework is detailed in the Supporting Information. For prognostic reviews, well‐conducted observational cohort studies start at a high certainty of evidence [22]. To estimate absolute effects, we calculated absolute risk differences (ARDs) using the relative measure of association (OR) and baseline risk, defined as the median risk of the outcome among individuals without the risk factor. To assess inconsistency, we visually inspected forest plots and considered the overlap in 95% CIs. In our assessment of imprecision, based on review team consensus, we considered a 2% ARD threshold to be a clinically meaningful risk factor.

## 3 Results

**FIGURE 1:**
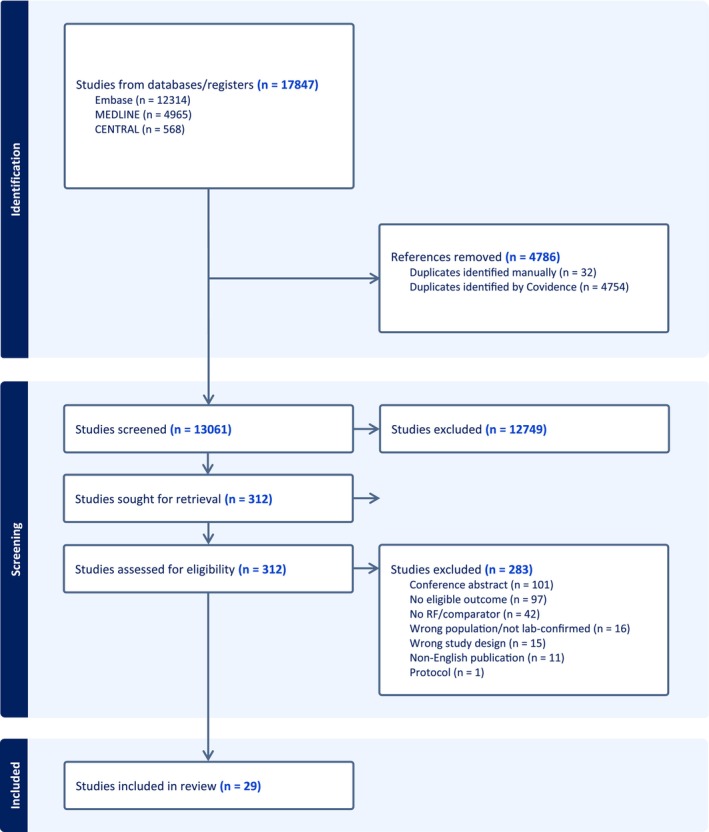
PRISMA flow diagram for study selection.

Our literature search identified 17,847 studies, and we screened 13,061 studies following de‐duplication. Three hundred and twelve full texts were retrieved for further review, and 29 studies reporting on 13,185 patients were finally included [7, 16, 23, 24, 25, 26, 27, 28, 29, 30, 31, 32, 33, 34, 35, 36, 37, 38, 39, 40, 41, 42, 43, 44, 45, 46, 47, 48, 49]. Result S1 presents citations excluded during full‐text review. The PRISMA flowchart for our study selection process is illustrated in Figure 1.

Table 1 describes the characteristics of the included studies, of which 25 were cohort studies, three were cross‐sectional studies, and one was a case‐control study. The studies included a median of 161 (range: 11–3740) participants with a median mean age of 3.3 (range of means: 0.5–71.6) years. Eleven studies included pediatric patients exclusively [23, 25, 26, 27, 28, 30, 33, 35, 40, 43, 47] and 10 studies included adult patients exclusively [7, 16, 24, 29, 32, 39, 41, 42, 44, 48]. Most studies (*n* = 10) were conducted in Asia [7, 24, 28, 29, 38, 40, 41, 42, 47, 48], followed by Europe (*n* = 9) [16, 26, 31, 32, 35, 36, 37, 44, 45], North America (*n* = 8) [23, 27, 30, 34, 39, 43, 46, 49], and Africa (*n* = 2) [25, 33]. Table S5 presents the 220 risk factor–outcome combinations not amenable to meta‐analysis since they were reported only by a single study. Finally, 14 unique risk factor–outcome combinations were amenable to meta‐analysis.

**TABLE 1:** Characteristics of included studies (*n* = 29).

Study	Country	Design	Recruitment period	Eligible participants	Mean ± SD age (years)	Age group	Female (%)
Amarin 2023	United States	Cohort	Nov 2015–Jul 2016	406	3.81 ± 4.06	Pediatrics	39.16%
Bahabri 2022	Saudi Arabia	Cohort	Jan 2016–Dec 2019	106	71.5 ± 29.63	Adults	52.83%
Baillie 2020	South Africa	Case‐control	Aug 2011–Aug 2013	422	0.56 ± 0.62	Pediatrics	49.53%
Bruning 2015	Netherlands	Cohort	Nov 2009–Dec 2012	120	1.20 ± 0.47	Pediatrics	41.67%
Cherry 1967	United States	Cohort	Jan 1964–May 1964	11	1.64 ± 2.15	Pediatrics	36.36%
Cheuk 2007	Hong Kong	Cohort	Aug 2001–Jul 2002	151	3.34 ± 2.47	Pediatrics	46.36%
Choi 2015	South Korea	Cohort	Jun 2012–May 2013	165	65.11 ± 14.49	Adults	31.52%
Chu 2016	United States	Cohort	Dec 2011–May 2013	445	0.94 ± 1.08	Pediatrics	38.43%
Comte 2020	France	Cohort	Oct 2015–Sep 2016	169	0.54 ± NR	Both	43.20%
Corne 2002	United Kingdom	Cohort	Sep 1993–Dec 1993	51	NR	Adults	47.06%
Esposito 2012	Burundi	Cohort	Nov 2010–Oct 2011	186	1.77 ± 2.14	Pediatrics	47.31%
Galindo‐Fraga 2013	Mexico	Cohort	Apr 2010–Apr 2011	161	NR	Both	NR
Garcia‐Garcia 2015	Spain	Cohort	Oct 2004–Jul 2011	534	NR	Pediatrics	NR
Gerna 2009	Italy	Cohort	Nov 2006–May 2007	148	NR	Both	NR
Goka 2015	United Kingdom	Cross‐sectional	Jan 2007–Jun 2012	3740	NR	Both	46.66%
Hung 2017	Hong Kong	Cohort	Mar 2014–Feb 2015	728	71.55 ± 20.21	Adults	55.36%
Hung 2019	Taiwan	Cohort	Jan 2013–Dec 2014	76	3.85 ± NR	Both	40.79%
Jacobs 2013	United States	Cohort	Mar 2008–Apr 2011	63	55.00 ± 12.50	Adults	39.68%
Kellner 1988	Vietnam	Cohort	Sep 1984–May 1986	60	NR	Pediatrics	NR
Kim 2019	South Korea	Cohort	Jan 2010–Dec 2015	410	NR	Adults	NR
Lee 2021	South Korea	Cohort	Nov 2012–Nov 2017	85	NR	Adults	NR
Nicholson 1996	United Kingdom	Cohort	Apr 1992–Mar 1994	96	NR	Adults	NR
Perez 2015	United States	Cohort	Feb 2013–Jun 2014	116	0.78 ± 0.60	Pediatrics	37.93%
Pierangeli 2011	Italy	Cohort	Feb 2009–Mar 2010	27	70.90 ± 19.60	Adults	NR
Piralla 2012	Italy	Cohort	Oct 2008–Sep 2009	296	NR	Both	NR
Sanchez‐Codez 2021	United States	Cross‐sectional	Jul 2011–Dec 2013	2473	1.20 ± 2.78	Both	41.00%
Song 2023	Hong Kong	Cross‐sectional	Jan 2015–Dec 2016	1330	3.15 ± 4.26	Pediatrics	38.57%
To 2016	Hong Kong	Cohort	Jan 2011–Dec 2013	22	56.86 ± 20.07	Adults	31.82%
Waghmare 2019	United States	Cohort	Jan 2009–Apr 2016	588	42.80 ± 26.30	NR	40.65%

Abbreviation: NR = not reported.

### 3.1 Risk of Bias of the Included Studies

The median NOS score for risk of bias was 7 (range: 5–9) out of a maximum of 9 (Tables S6 and S7). The two most frequent sources of bias were poor comparability of risk factors and reference cohorts and poor representation of the population at risk. Of note, only seven studies (24.1%) adequately controlled or adjusted for important confounding variables, including age, and 15 studies (51.7%) did not control or adjust for any variables. Furthermore, 16 (55.2%) studies were only somewhat representative of the population at risk, and six (20.7%) were inadequately representative. Common reasons for poor representativeness include restricted sampling frames and nonconsecutive enrolment. The complete risk of bias assessments are presented in Tables S6 and S7. We were unable to assess publication bias due to an insufficient number of studies.

### 3.2 Demographic Characteristic Risk Factors

Our review identified six risk factors related to patients' demographic characteristics that were amenable to meta‐analysis (Table 2 and Figures S1–S6). We found with high certainty that age less than 1 year (OR 1.04, 95% CI 0.73–1.46; ARD 1 more per 1000 participants, 95% CI 7 fewer to 12 more; *I* ^2^ = 0%) and premature birth (OR 1.32, 95% CI 0.97–1.81; ARD 7 more per 1000, 95% CI 1 fewer to 19 more; *I* ^2^ = 0%) are both not associated with the risk of LRTI. With moderate certainty in the evidence, age greater than 18 years is probably associated with an increased risk of mortality (OR 8.99, 95% CI 2.46–32.91; ARD 114 more per 1000, 95% CI 23 more to 337 more; *I* ^2^ = 0%) and male sex is probably not associated with the risk of admission to a CCU (OR 1.03, 95% CI 0.87–1.23; ARD 1 more per 1000, 95% CI 3 fewer to 6 more; *I* ^2^ = 0%). The evidence is very uncertain on the prognostic values of smoking and male sex as risk factors for LRTI. We were unable to perform our planned subgroup analysis by age due to an insufficient number of studies.

**TABLE 2:** Summary of findings of the demographic risk factors for severe or complicated rhinovirus illness.

Predictor and outcome	Study results and measurements	Prevalence	Absolute effect estimates		Certainty of the evidence	Plain language summary
			Baseline	With predictor		
Age < 1 year LRTI	Odds ratio: 1.04 (95% CI 0.73–1.46) Based on data from 608 participants in two studies	45.56%	27 per 1000	28 per 1000	**High**	Age < 1 year is not associated with the risk of LRTI
			Difference: 1 more per 1000 (95% CI 7 fewer—12 more)			
Age > 18 years Mortality	Odds ratio: 8.99 (95% CI 2.46–32.91) Based on data from 237 participants in two studies	8.44%	16 per 1000	130 per 1000	**Moderate** Due to serious imprecision	Age > 18 years is probably associated with an increased risk of mortality
			Difference: 114 more per 1000 (95% CI 23 more—337 more)			
Male sex Admission to a CCU	Odds ratio: 1.03 (95% CI 0.87–1.23) Based on data from 2657 participants in three studies	22.83%	27 per 1000	28 per 1000	**Moderate** Due to serious inconsistency	Male sex is probably not associated with the risk of admission to a CCU
			Difference: 1 more per 1000 (95% CI 3 fewer—6 more)			
Male sex LRTI	Odds ratio: 1.30 (95% CI 0.78–2.18) Based on data from 1092 participants in five studies	48.08%	24 per 1000	31 per 1000	**Very low** Due to very serious inconsistency and serious imprecision	We are very uncertain about the prognostic value of male sex on the risk of LRTI
			Difference: 7 more per 1000 (95% CI 5 fewer—27 more)			
Premature birth LRTI	Odds ratio: 1.32 (95% CI 0.97–1.81) Based on data from 979 participants in two studies	41.37%	24 per 1000	31 per 1000	**High**	Premature birth is not associated with the risk of LRTI
			Difference: 7 more per 1000 (95% CI 1 fewer—19 more)			
Smoking LRTI	Odds ratio: 0.72 (95% CI 0.11–4.70) Based on data from 750 participants in two studies	42.86%	31 per 1000	22 per 1000	**Very low** Due to serious inconsistency and very serious imprecision	We are very uncertain about the prognostic value of smoking on the risk of LRTI
			Difference: 8 fewer per 1000 (95% CI 27 fewer—98 more)			

Abbreviations: CCU = critical care unit, CI = confidence interval, LRTI = lower respiratory tract infection.

### 3.3 Comorbidity Risk Factors

We identified seven risk factors related to comorbidities that were amenable to meta‐analysis: any comorbidity and admission to a CCU, malignancy and mortality, diabetes mellitus and mortality, stroke and mortality, pulmonary comorbidity and LRTI, any comorbidity and LRTI, and malignancy and admission to a CCU (Table 3 and Figures S7–S13). We found with high certainty that diabetes mellitus is not associated with the risk of mortality (OR 0.84, 95% CI 0.54–1.31; ARD 5 fewer per 1000, 95% CI 13 fewer to 8 more; *I* ^2^ = 0%). With moderate certainty in the evidence, we found that the presence of any comorbidity (OR 1.79, 95% CI 1.04–3.06; ARD 20 more per 1000, 95% CI 1 more to 51 more; *I* ^2^ = 82.83%) or pulmonary comorbidity (OR 4.48, 95% CI 2.62–7.65; ARD 35 more per 1000, 17 more to 65 more; *I* ^2^ = 0%) are each probably associated with an increased risk of LRTI, and malignancy is probably associated with an increased risk of admission to a CCU (OR 2.23, 95% CI 0.62–8.11; ARD 25 more per 1000, 8 fewer to 128 more; *I* ^2^ = 0%) and an increased risk of mortality (OR 1.97, 95% CI 1.17–3.31; ARD 22 more per 1000, 95% CI 4 more to 51 more; *I* ^2^ = 17.57%). We found with low certainty that the presence of any comorbidity may not be associated with admission to a CCU (OR 1.42, 95% CI 0.65–3.13; ARD 10 more per 1000, 95% CI 8 fewer to 47 more; *I* ^2^ = 64.88%), and stroke may not be associated with mortality (OR 1.24, 95% CI 0.68–2.26; ARD 6 more per 1000, 95% CI 8 fewer to 31 more; *I* ^2^ = 0%). Studies reporting on the presence of any comorbidity commonly assessed for cardiac, hematologic, genetic, metabolic, and neurologic comorbidities, immunodeficiency, and malignancy. A subgroup analysis by age was not possible due to an insufficient number of studies.

**TABLE 3:** Summary of findings of the comorbidity risk factors for severe or complicated rhinovirus illness.

Predictor and outcome	Study results and measurements	Prevalence	Absolute effect estimates		Certainty of the evidence	Plain language summary
			Baseline	With predictor		
Any comorbidity Admission to a CCU	Odds ratio: 1.42 (95% CI 0.65–3.13) Based on data from 2627 participants in three studies	33.33%	24 per 1000	33 per 1000	**Low** Due to serious inconsistency and serious imprecision	Any comorbidity may not be associated with the risk of admission to a CCU
			Difference: 10 more per 1000 (95% CI 8 fewer—47 more)			
Any comorbidity LRTI	Odds ratio: 1.79 (95% CI 1.04–3.06) Based on data from 541 patients in two studies)	33.27%	27 per 1000	47 per 1000	**Moderate** Due to serious imprecision	Any comorbidity is probably associated with an increased risk of LRTI
			Difference: 20 more per 1000 (95% CI 1 more—51 more)			
Diabetes mellitus Mortality	Odds ratio: 0.84 (95% CI 0.54–1.31) Based on data from 750 patients in two studies)	17.20%	28 per 1000	23 per 1000	**High**	Diabetes mellitus is not associated with the risk of mortality
			Difference: 5 fewer per 1000 (95% CI 13 fewer—8 more)			
Malignancy Admission to a CCU	Odds ratio: 2.23 (95% CI 0.62–8.11) Based on data from 184 participants in two studies	22.83%	21 per 1000	46 per 1000	**Moderate** Due to serious imprecision	Malignancy is probably associated with an increased risk of admission to a CCU
			Difference: 25 more per 1000 (95% CI 8 fewer—128 more)			
Malignancy Mortality	Odds ratio: 1.97 (95% CI 1.17–3.31) Based on data from 1303 participants in three studies	13.74%	24 per 1000	46 per 1000	**Moderate** Due to serious imprecision	Malignancy is probably associated with an increased risk of mortality
			Difference: 22 more per 1000 (95% CI 4 more—51 more)			
Pulmonary comorbidity LRTI	Odds ratio: 4.48 (95% CI 2.62–7.65) Based on data from 596 participants in two studies	45.97%	11 per 1000	46 per 1000	**Moderate** Due to serious imprecision	Pulmonary comorbidity is probably associated with an increased risk of LRTI
			Difference: 35 more per 1000 (95% CI 17 more—65 more)			
Stroke Mortality	Odds ratio: 1.24 (95% CI 0.68–2.26) Based on data from 750 participants in two studies	17.20%	26 per 1000	32 per 1000	**Low** Due to serious inconsistency and serious imprecision	Stroke may not be associated the risk of mortality
			Difference: 6 more per 1000 (95% CI 8 fewer—31 more)			

Abbreviations: CCU = critical care unit, CI = confidence interval, LRTI = lower respiratory tract infection.

### 3.4 Risk Factors Not Amenable to Meta‐Analysis

Several risk factor–outcome combinations were reported by only one study and were not amenable to meta‐analysis (Table S5). Although age was investigated by many studies, different thresholds prevented pooling of effect estimates. In adults, age > 65 years may increase the risk of hospitalization (OR 36.06, 95% CI 2.59–2306.93) [44]. In children, there was conflicting evidence on the effect of younger age on rhinovirus illness severity. In the general population, two studies found that adults tended to have an increased risk of hospitalization, mortality, and LRTI, and admission to a CCU compared to children [34, 38]. Race, ethnicity, a plethora of comorbid conditions, and several environmental factors were assessed in single studies, of which most did not have statistically significant associations. Of note, living in a nursing home was associated with increased mortality (adjusted OR 4.59, 95% CI 3.02–7.04) [7]. Several risk factors relating to transplant recipients were reported, although there were insufficient studies to pool estimates.

## 4 Discussion

This systematic review and meta‐analysis identified several risk factors for severe outcomes of rhinovirus infection. With high certainty in the evidence, among patients with rhinovirus illness, we found that age less than 1 year and premature birth are not associated with LRTI, and diabetes mellitus is not associated with mortality. We found with moderate certainty that age greater than 18 years and malignancy are probably associated with increased mortality, pulmonary comorbidity and the presence of any comorbidity are probably associated with an increased risk of LRTI, malignancy is probably associated with an increased risk of admission to a CCU, and sex is probably not associated with the risk of admission to a CCU. With low certainty, we found that any comorbidity may not be associated with the risk of admission to a CCU, and stroke may not be associated with a risk of mortality.

To the best of our knowledge, our review is the first to systematically synthesize evidence pertaining to the populations at risk for severe or complicated rhinovirus illness. Our findings agree with previous studies that those with underlying health conditions are more likely to develop severe presentations of rhinovirus infection [50, 51]. However, we were unable to confirm the role of age in rhinovirus illness complications due to the paucity of evidence [52]. Furthermore, since parents may bring younger children to the doctor for less severe symptoms, the effect of lower age in children may be underestimated [53]. Similarly, those with fewer comorbidities may less frequently solicit care, contributing to an overrepresentation of severe cases of rhinovirus illness among healthier populations. Our findings for age as a risk factor must be interpreted in context: Studies reporting on age greater than 1 year as a risk factor only included young children, and the increased risk of mortality associated with age greater than 18 years is likely driven by the elderly population.

Our review is particularly relevant with the ongoing search for effective rhinovirus therapies. Although no pharmaceutical treatments are currently approved for rhinovirus infection, several candidates show promise. Ribavirin, co‐administered with pegylated interferon α, is used to treat respiratory syncytial virus and hepatitis C infections by impairing viral RNA synthesis [12]. Ribavirin has shown promise in reducing viral replication and clearing rhinovirus RNA in a small case series and a case report [12, 13]. Vapendavir, a capsid‐binding inhibitor, has shown efficacy in reducing upper respiratory symptoms and excellent tolerability in a randomized, double‐blind, placebo‐controlled phase 2 trial [14]. Other treatments have been studied indirectly in relevant populations, such as azithromycin for the prevention of asthma exacerbations, or have not yet progressed to human trials [15]. Identifying populations with the greatest risk of severe outcomes from rhinovirus infections who would benefit most from therapies is important to maximize their impact. Furthermore, in the absence of effective treatments, knowledge of populations with increased risk of severe rhinovirus illness can reduce usage of unnecessary testing and medication in clinical settings [10].

### 4.1 Strength and Limitations of the Evidence and Our Systematic Review and Meta‐Analysis

While many included studies were of moderate to high quality based on NOS assessment, several limitations in the available evidence reduce the certainty and generalizability of our findings. The number of studies available for each meta‐analysis was limited, which constrained the breadth of our meta‐analyses. Some studies had relatively small sample sizes, contributing to imprecise effect estimates. Almost all studies were performed at a single site, negatively affecting the generalizability of the findings.

In addition, variation in the measurement and reporting of risk factors among the included studies impeded our ability to pool data, as many risk factors were only reported by a single study. For example, studies that assessed for “any comorbidity” as a risk factor were more frequently amenable to meta‐analysis, while those that reported specific comorbid conditions were often unpooled. Conditions such as asthma and chronic obstructive pulmonary disease that have previously been suggested to be associated with increased severity of rhinovirus infections were unable to be meta‐analyzed [5]. Future research into patients receiving transplants could be especially impactful given their immunocompromised status. While several studies reported on these participants, differences in the reported risk factors prevented meta‐analysis. Further, some studies used unclear categories of comorbidities, preventing pooling between studies. Additionally, the discretization of continuous variables and employment of different thresholds between studies limited the data available for pooling. This was especially prominent for age, where various cutoffs were reported, especially for studies investigating pediatric populations. Future studies should use commonly reported cutoffs or those used in previous studies to increase consistency of the evidence and facilitate meta‐analysis [54].

Methodological differences between studies also led to challenges in interpretation. Several risk factor–outcome combinations showed high heterogeneity, reducing the generalizability of the pooled effect sizes. Most risk factor–outcome combinations that had heterogeneous findings were due to differences in the direction of the effect. Common differences between studies include study setting, study population (including age and comorbidities at baseline), and duration of follow‐up. The lack of adequate adjustment for key confounding variables in 22 studies (75.9%) also introduces the potential for bias in our findings. These limitations in the evidence illustrate a need for more comprehensive studies assessing populations at risk of severe or complicated rhinovirus illness.

Aside from limitations stemming from the quality and availability of evidence, our review has some notable limitations. We were unable to assess for publication bias due to the small number of studies available for each risk factor–outcome combination. Furthermore, the NOS was originally designed for cohort and case‐control studies, not cross‐sectional ones. However, this limitation is only relevant for a few of the included studies. Additionally, given the limited empirical data to support a specific minimally important difference, our choice of a 2% ARD threshold, although based on team consensus, may not reflect the values of all stakeholders.

Our review is strengthened by the comprehensive search strategy, which did not place restrictions on publication date. When a study reported multiple models, we included only the most comprehensively adjusted models. We also captured a broader range of evidence by including studies reporting proportions and deriving odds ratios from their results. We rigorously assessed the included studies for risk of bias and employed the GRADE approach to report the certainty in the evidence. In addition, we presented our findings using ARDs, which improve the clinical contextualization of our findings.

## 5 Conclusion

In this systematic review and meta‐analysis, we identified several populations at risk for the development of severe or complicated rhinovirus illness. Individuals with age greater than 18 years, malignancy, pulmonary comorbidity, or any comorbidity are more likely to experience a severe course of illness. However, age of less than 1 year, preterm birth, diabetes mellitus, or sex are not associated with the severity of rhinovirus infection. To complement the current search for treatments, further research is required to explore and validate other risk factors of interest.

## Author Contributions

**David Gou:** writing – original draft, methodology, formal analysis, investigation, validation, conceptualization. **Jessica Bartoszko:** writing – review and editing, methodology, formal analysis, supervision, project administration, validation, investigation, conceptualization. **Laura Weiler:** writing – review and editing, investigation. **Asal Alavi Parsi:** writing – review and editing, investigation. **Ante Cuvalo:** writing – review and editing, investigation. **Sahith Rajkumar:** writing – review and editing, investigation. **Dominik Mertz:** writing – review and editing, methodology, formal analysis, supervision, project administration, validation, investigation, conceptualization. **Mark Loeb:** writing – review and editing, methodology, formal analysis, resources, project administration, validation, supervision, investigation, conceptualization.

## Funding

The authors have nothing to report.

## Ethics Statement

This work did not require IRB approval because it relies wholly upon previously published literature without personal health information.

## Conflicts of Interest

The authors declare no conflicts of interest.

## Supporting information




**Table S1:** Completed PRISMA 2020 checklist.
**Table S2:** CENTRAL search strategy from inception to April 4, 2024.
**Table S3:** EMBASE search strategy from inception to April 4, 2024.
**Table S4:** MEDLINE search strategy from inception to April 4, 2024.
**Table S5:** Risk factor–outcome combinations not amenable to meta‐analysis.
**Table S6:** Newcastle–Ottawa Scale risk of bias assessments for the included cohort studies (*n* = 25).
**Table S7:** Newcastle–Ottawa Scale risk of bias assessments for the included case‐control study (*n* = 1).
**Table S8:** Newcastle–Ottawa Scale risk of bias assessments for the included cross‐sectional studies (*n* = 3).
**Result S1.** Citations of excluded full‐text studies.
**Figure S1:** Age < 1 year as a risk factor for lower respiratory tract infection.
**Figure S2:** Age ≥ 18 years as a risk factor for mortality.
**Figure S3:** Male sex as a risk factor for admission to a critical care unit.
**Figure S4:** Male sex as a risk factor for lower respiratory tract infection.
**Figure S5:** Premature birth as a risk factor for lower respiratory tract infection.
**Figure S6:** Smoking as a risk factor for lower respiratory tract infection.
**Figure S7:** Forest plot for any comorbidity as a risk factor for admission to a critical care unit.
**Figure S8:** Forest plot for any comorbidity as a risk factor for lower respiratory tract infection.
**Figure S9:** Forest plot for diabetes mellitus as a risk factor for mortality.
**Figure S10:** Malignancy as a risk factor for admission to a critical care unit.
**Figure S11:** Malignancy as a risk factor for mortality.
**Figure S12:** Pulmonary comorbidity as a risk factor for lower respiratory tract infection.
**Figure S13:** Stroke as a risk factor for mortality.

## Data Availability

The data that support the findings of this study are available in the Supporting Information of this article.
