# Supplementary material for: Populations at Risk for Severe or Complicated Rhinovirus Illness: A Systematic Review and Meta‐Analysis
Source: Influenza Other Respir Viruses. 2026 Mar 18;20(3):e70251. doi: 10.1111/irv.70251 (PMC13098166; doi:10.1111/irv.70251)

Supplemental material for **Populations at risk for severe or complicated rhinovirus illness: A systematic review and meta-analysis**

David Gou, BHSc^1,2^, Jessica Bartoszko, PhD^3^, Laura Weiler^4^, Asal Alavi Parsi^2^, Ante Cuvalo^2^, Sahith Rajkumar^2^, Dominik Mertz, MD, MSc^5,6^, Mark Loeb, MD, MSc^5,6^*

^1^ Temerty Faculty of Medicine, University of Toronto, Toronto, Ontario, Canada

^2^ Faculty of Health Sciences, McMaster University, Hamilton, Ontario, Canada

^3^ Department of Health Research Methods, Evidence and Impact, McMaster University, Hamilton, Canada

^4^ Faculty of Engineering, McMaster University, Hamilton, Ontario, Canada

^5^ Division of Infectious Diseases, Department of Medicine, McMaster University, Hamilton, Ontario, Canada

^6^ Division of Microbiology, Department of Pathology and Laboratory Medicine, McMaster University, Hamilton, Ontario, Canada

**Corresponding author***

Mark Loeb

Department of Medicine

McMaster University

1280 Main St W,

Hamilton, ON L8S 4K1

Canada

Email: [loebm@mcmaster.ca](mailto:loebm@mcmaster.ca)

Contents

[Supplemental Methods. Detailed approach to the assessment of the certainty of evidence using the GRADE framework. 3](#_Toc217331789)

[Table S1. Completed PRISMA 2020 checklist. 5](#_Toc217331790)

[Table S2. CENTRAL search strategy from inception to April 4, 2024. 8](#_Toc217331791)

[Table S3. EMBASE search strategy from inception to April 4, 2024. 8](#_Toc217331792)

[Table S4. MEDLINE search strategy from inception to April 4, 2024. 8](#_Toc217331793)

[Table S5. Risk factor-outcome combinations not amenable to meta-analysis. 10](#_Toc217331794)

Table S6. Newcastle-Ottawa Scale risk of bias assessments for the included cohort studies…………………………

Table S7 Newcastle-Ottawa Scale risk of bias assessments for the included case-control study…………………….

Table S8 Newcastle-Ottawa Scale risk of bias assessments for cross sectional studies………………………………

[Result S1. Citations of excluded full-text studies. 21](#_Toc217331795)

[Figure S1. Age <1 year as a risk factor for lower respiratory tract infection. 38](#_Toc217331796)

[Figure S2. Age ≥18 years as a risk factor for mortality. 38](#_Toc217331797)

[Figure S3. Male sex as a risk factor for admission to a critical care unit. 39](#_Toc217331798)

[Figure S4. Male sex as a risk factor for lower respiratory tract infection. 39](#_Toc217331799)

[Figure S5. Premature birth as a risk factor for lower respiratory tract infection. 40](#_Toc217331800)

[Figure S6. Smoking as a risk factor for lower respiratory tract infection. 40](#_Toc217331801)

[Figure S7. Forest plot for any comorbidity as a risk factor for admission to a critical care unit. 41](#_Toc217331802)

[Figure S8. Forest plot for any comorbidity as a risk factor for lower respiratory tract infection. 41](#_Toc217331803)

[Figure S9. Forest plot for diabetes mellitus as a risk factor for mortality. 42](#_Toc217331804)

[Figure S10. Malignancy as a risk factor for admission to a critical care unit. 42](#_Toc217331805)

[Figure S11. Malignancy as a risk factor for mortality. 43](#_Toc217331806)

[Figure S12. Pulmonary comorbidity as a risk factor for lower respiratory tract infection. 43](#_Toc217331807)

[Figure S13. Stroke as a risk factor for mortality. 44](#_Toc217331808)

# **Supplemental Methods.** Detailed approach to the assessment of the certainty of evidence using the GRADE framework.

For reviews of prognosis, each meta-analysis starts with a high certainty of evidence.^1^ Each GRADE domain is rated as “not serious,” “serious” (downgrade once), “very serious” (downgrade twice), or “extremely serious” (downgrade three times), with the final GRADE rating determined by the aggregation of all the domains: high (not downgraded), moderate (downgraded once), low (downgraded twice), or very low (downgraded three or more times).

**Risk of bias**

We used the Newcastle-Ottawa Scale to assess the risk of bias and classified each study as low or high risk of bias overall.^2^ This scale produces a quantitative rating with a maximum score of nine points across three domains: selection of the study population, comparability of cases and controls, and ascertainment of exposure and outcome. We used a conservative threshold of 7 or more points to indicate a low risk of bias, and 6 or fewer points to indicate a high risk of bias.

For each meta-analysis, we downgraded the certainty of the evidence once when studies with a high risk of bias contributed ≥ 50% of the weight of the pooled effect estimates, and twice when such studies contributed ≥ 80% of the weight.

**Inconsistency**

We used visual inspection of the forest plots and the I^2^ statistic to assess inconsistency. For visual inspection of the forest plots, we considered the variability in point estimates in relation to the null and the overlap of confidence intervals across studies. For the I^2^ statistic, we considered 50–90% to suggest substantial heterogeneity, and 75–100% to suggest considerable heterogeneity.^3^ When rating down, we placed particular emphasis on the visual inspection of the forest plots, as the I^2^ statistic must be interpreted in context.^4^

**Imprecision**

If the confidence interval crosses the minimally important difference (2% absolute risk difference) threshold (i.e., includes both no effect and an important benefit or harm), we rated down once for imprecision. If the confidence interval spans both important benefit and important harm, we rated down twice for imprecision. If the confidence interval does not cross either 2% absolute risk difference threshold, we did not rate down. However, we also considered the ratio of the upper confidence interval and the lower confidence interval, with ratios greater than 2.5 suggesting imprecision and supporting a decision to rate down.

**Indirectness**

If the study sample, risk factor, or outcome reported by individual studies did not accurately reflect the review question, we rated down for indirectness.

**Publication bias**

If publication bias was detected via visual inspection of funnel plots, we rated down for publication bias.

**Citations**

1. Foroutan F, Guyatt G, Zuk V, et al. GRADE Guidelines 28: Use of GRADE for the assessment of evidence about prognostic factors: rating certainty in identification of groups of patients with different absolute risks. *J Clin Epidemiol*. 2020;121:62-70. doi:10.1016/j.jclinepi.2019.12.023

2. Wells G, Shea B, O’Connell D, et al. The Newcastle-Ottawa Scale (NOS) for assessing the quality of nonrandomised studies in meta-analyses. Accessed November 13, 2024. https://www.ohri.ca/programs/clinical_epidemiology/oxford.asp

3. Guyatt GH, Oxman AD, Kunz R, et al. GRADE guidelines: 7. Rating the quality of evidence—inconsistency. *J Clin Epidemiol*. 2011;64(12):1294-1302. doi:10.1016/j.jclinepi.2011.03.017

4. Guyatt G, Zhao Y, Mayer M, et al. GRADE guidance 36: updates to GRADE’s approach to addressing inconsistency. *J Clin Epidemiol*. 2023;158:70-83. doi:10.1016/j.jclinepi.2023.03.003

# **Table S1**. Completed PRISMA 2020 checklist.

| **Section and Topic** | **Item #** | **Checklist item** | **Location where item is reported** |
| --- | --- | --- | --- |
| **TITLE** | | |  |
| Title | 1 | Identify the report as a systematic review. | Page 1 |
| **ABSTRACT** | | |  |
| Abstract | 2 | See the PRISMA 2020 for Abstracts checklist. | N/A |
| **INTRODUCTION** | | |  |
| Rationale | 3 | Describe the rationale for the review in the context of existing knowledge. | Pages 4–5 |
| Objectives | 4 | Provide an explicit statement of the objective(s) or question(s) the review addresses. | Page 5 |
| **METHODS** | | |  |
| Eligibility criteria | 5 | Specify the inclusion and exclusion criteria for the review and how studies were grouped for the syntheses. | Pages 5–6 |
| Information sources | 6 | Specify all databases, registers, websites, organisations, reference lists and other sources searched or consulted to identify studies. Specify the date when each source was last searched or consulted. | Page 5,  Tables S2–S4 |
| Search strategy | 7 | Present the full search strategies for all databases, registers and websites, including any filters and limits used. | Tables S2–S4 |
| Selection process | 8 | Specify the methods used to decide whether a study met the inclusion criteria of the review, including how many reviewers screened each record and each report retrieved, whether they worked independently, and if applicable, details of automation tools used in the process. | Page 6 |
| Data collection process | 9 | Specify the methods used to collect data from reports, including how many reviewers collected data from each report, whether they worked independently, any processes for obtaining or confirming data from study investigators, and if applicable, details of automation tools used in the process. | Page 6 |
| Data items | 10a | List and define all outcomes for which data were sought. Specify whether all results that were compatible with each outcome domain in each study were sought (e.g. for all measures, time points, analyses), and if not, the methods used to decide which results to collect. | Pages 5–6 |
|  | 10b | List and define all other variables for which data were sought (e.g. participant and intervention characteristics, funding sources). Describe any assumptions made about any missing or unclear information. | Page 6 |
| Study risk of bias assessment | 11 | Specify the methods used to assess risk of bias in the included studies, including details of the tool(s) used, how many reviewers assessed each study and whether they worked independently, and if applicable, details of automation tools used in the process. | Page 7 |
| Effect measures | 12 | Specify for each outcome the effect measure(s) (e.g. risk ratio, mean difference) used in the synthesis or presentation of results. | Page 6 |
| Synthesis methods | 13a | Describe the processes used to decide which studies were eligible for each synthesis (e.g. tabulating the study intervention characteristics and comparing against the planned groups for each synthesis (item #5)). | Page 6 |
|  | 13b | Describe any methods required to prepare the data for presentation or synthesis, such as handling of missing summary statistics, or data conversions. | Pages 6–7 |
|  | 13c | Describe any methods used to tabulate or visually display results of individual studies and syntheses. | Pages 6–7 |
|  | 13d | Describe any methods used to synthesize results and provide a rationale for the choice(s). If meta-analysis was performed, describe the model(s), method(s) to identify the presence and extent of statistical heterogeneity, and software package(s) used. | Pages 6–7 |
|  | 13e | Describe any methods used to explore possible causes of heterogeneity among study results (e.g. subgroup analysis, meta-regression). | Page 7 |
|  | 13f | Describe any sensitivity analyses conducted to assess robustness of the synthesized results. | N/A |
| Reporting bias assessment | 14 | Describe any methods used to assess risk of bias due to missing results in a synthesis (arising from reporting biases). | Pages 6–7 |
| Certainty assessment | 15 | Describe any methods used to assess certainty (or confidence) in the body of evidence for an outcome. | Pages 7–8, Supplemental Methods |
| **RESULTS** | | |  |
| Study selection | 16a | Describe the results of the search and selection process, from the number of records identified in the search to the number of studies included in the review, ideally using a flow diagram. | Page 8, Figure 1 |
|  | 16b | Cite studies that might appear to meet the inclusion criteria, but which were excluded, and explain why they were excluded. | Result S1 |
| Study characteristics | 17 | Cite each included study and present its characteristics. | Page 8, Table 1 |
| Risk of bias in studies | 18 | Present assessments of risk of bias for each included study. | Pages 8–9,  Tables S6–S7 |
| Results of individual studies | 19 | For all outcomes, present, for each study: (a) summary statistics for each group (where appropriate) and (b) an effect estimate and its precision (e.g. confidence/credible interval), ideally using structured tables or plots. | Pages 9–11, Tables 2–3 |
| Results of syntheses | 20a | For each synthesis, briefly summarise the characteristics and risk of bias among contributing studies. | Pages 9–10, Tables 2–3 |
|  | 20b | Present results of all statistical syntheses conducted. If meta-analysis was done, present for each the summary estimate and its precision (e.g. confidence/credible interval) and measures of statistical heterogeneity. If comparing groups, describe the direction of the effect. | Pages 9–10, Tables 2–3, Figures S1–S13 |
|  | 20c | Present results of all investigations of possible causes of heterogeneity among study results. | Pages 9–10 |
|  | 20d | Present results of all sensitivity analyses conducted to assess the robustness of the synthesized results. | N/A |
| Reporting biases | 21 | Present assessments of risk of bias due to missing results (arising from reporting biases) for each synthesis assessed. | Page 9 |
| Certainty of evidence | 22 | Present assessments of certainty (or confidence) in the body of evidence for each outcome assessed. | Pages 9–10, Tables 2–3 |
| **DISCUSSION** | | |  |
| Discussion | 23a | Provide a general interpretation of the results in the context of other evidence. | Pages 11–16 |
|  | 23b | Discuss any limitations of the evidence included in the review. | Pages 13–14 |
|  | 23c | Discuss any limitations of the review processes used. | Pages 14 |
|  | 23d | Discuss implications of the results for practice, policy, and future research. | Pages 12–13 |
| **OTHER INFORMATION** | | |  |
| Registration and protocol | 24a | Provide registration information for the review, including register name and registration number, or state that the review was not registered. | Page 5 |
|  | 24b | Indicate where the review protocol can be accessed, or state that a protocol was not prepared. | Page 5 |
|  | 24c | Describe and explain any amendments to information provided at registration or in the protocol. | Page 5 |
| Support | 25 | Describe sources of financial or non-financial support for the review, and the role of the funders or sponsors in the review. | Page 1 |
| Competing interests | 26 | Declare any competing interests of review authors. | Page 1 |
| Availability of data, code and other materials | 27 | Report which of the following are publicly available and where they can be found: template data collection forms; data extracted from included studies; data used for all analyses; analytic code; any other materials used in the review. | Page 1 |

# Table S2. CENTRAL search strategy from inception to April 4, 2024.

| Line # | Search Statement | Results |
| --- | --- | --- |
| #1 | (Rhinovirus, Human) | 320 |
| #2 | Rhinovirus | 541 |
| #3 | MeSH descriptor: [Rhinovirus] explode all trees | 182 |
| #4 | #1 or #2 or #3 | 541 |

# Table S3. EMBASE search strategy from inception to April 4, 2024.

| Line # | Search Statement | Results |
| --- | --- | --- |
| 1 | Rhinovirus, Human/ | 2725 |
| 2 | exp Rhinovirus/ | 10736 |
| 3 | Rhinovirus.mp. | 13304 |
| 4 | 1 or 2 or 3 | 13304 |
| 5 | exp risk/ | 3159208 |
| 6 | exp epidemics/ | 132645 |
| 7 | exp Mortality/ | 1396907 |
| 8 | exp Morbidity/ | 439794 |
| 9 | morbidit*.ti. | 46488 |
| 10 | ((mortality or risk or risks or morbidit* or severe or severity) adj7 rhinovirus).mp. | 1515 |
| 11 | Susceptibil:.ti. and rhinovirus.mp. | 77 |
| 12 | (Susceptibl* and rhinovirus).mp. | 337 |
| 13 | Death/ | 290763 |
| 14 | Disease Outbreaks/ | 60382 |
| 15 | exp Hospitalization/ | 543155 |
| 16 | risk factor*.mp. | 1807966 |
| 17 | exp Disease Transmission, Infectious/ | 249208 |
| 18 | (risk or risks or mortality).ti. | 1065882 |
| 19 | 5 or 6 or 7 or 8 or 9 or 10 or 11 or 12 or 13 or 14 or 15 or 16 or 17 or 18 | 5369820 |
| 20 | 4 and 19 | 4433 |
| 21 | 2 or 20 | 11557 |
| 22 | animals/ not humans/ | 1083791 |
| 23 | 21 not 22 | 11514 |

# Table S4. MEDLINE search strategy from inception to April 4, 2024.

| Line # | Search Statement | Results |
| --- | --- | --- |
| 1 | exp Rhinovirus/ | 4217 |
| 2 | Rhinovirus.mp. | 7188 |
| 3 | 1 or 2 | 7188 |
| 4 | exp risk/ | 1395326 |
| 5 | exp epidemics/ | 138633 |
| 6 | exp Mortality/ | 425145 |
| 7 | exp Morbidity/ | 655607 |
| 8 | morbidit*.ti. | 36631 |
| 9 | ((mortality or risk or risks or morbidit* or severe or severity) adj7 rhinovirus).mp. | 512 |
| 10 | Susceptibil:.ti. and rhinovirus.mp. | 42 |
| 11 | (Susceptibl* and rhinovirus).mp. | 174 |
| 12 | Death/ | 20451 |
| 13 | Disease Outbreaks/ | 92206 |
| 14 | exp Hospitalization/ | 296209 |
| 15 | risk factor*.mp. | 1403738 |
| 16 | exp Disease Transmission, Infectious/ | 81618 |
| 17 | (risk or risks or mortality).ti. | 783261 |
| 18 | 4 or 5 or 6 or 7 or 8 or 9 or 10 or 11 or 12 or 13 or 14 or 15 or 16 or 17 | 3208358 |
| 19 | 3 and 18 | 1770 |
| 20 | 1 or 19 | 5095 |
| 21 | animals/ not humans/ | 5142068 |
| 22 | 20 not 21 | 4865 |

# Table S5. Risk factor-outcome combinations not amenable to meta-analysis.

| Study | Risk factor | Outcome | Reference group or unit change | Measure of Effect | Measure of Effect Value (95% CI) |
| --- | --- | --- | --- | --- | --- |
| Amarin 2023 | age | All-cause hospitalization | per 1 year increase | Adjusted OR | 0.86 (0.8–0.93) |
| Amarin 2023 | male sex | All-cause hospitalization | female sex | Adjusted OR | 0.64 (0.4–1.02) |
| Amarin 2023 | non-Hispanic black | All-cause hospitalization | non-Hispanic white | Adjusted OR | 0.26 (0.15–0.46) |
| Amarin 2023 | non-Hispanic other | All-cause hospitalization | non-Hispanic white | Adjusted OR | 1.25 (0.56–2.82) |
| Amarin 2023 | Hispanic | All-cause hospitalization | non-Hispanic white | Adjusted OR | 0.34 (0.18–0.64) |
| Amarin 2023 | tobacco smoke exposure | All-cause hospitalization | Yes vs No | Adjusted OR | 0.92 (0.57–1.48) |
| Amarin 2023 | history of asthma | All-cause hospitalization | Yes vs No | Adjusted OR | 3.37 (1.8–6.32) |
| Amarin 2023 | age | All-cause hospitalization | per 1 year increase | Adjusted OR | 0.83 (0.76–0.91) |
| Amarin 2023 | male sex | All-cause hospitalization | female sex | Adjusted OR | 0.56 (0.34–0.93) |
| Amarin 2023 | non-Hispanic black | All-cause hospitalization | non-Hispanic white | Adjusted OR | 0.27 (0.14–0.51) |
| Amarin 2023 | non-Hispanic other | All-cause hospitalization | non-Hispanic white | Adjusted OR | 0.99 (0.4–2.44) |
| Amarin 2023 | Hispanic | All-cause hospitalization | non-Hispanic white | Adjusted OR | 0.34 (0.17–0.69) |
| Amarin 2023 | tobacco smoke exposure | All-cause hospitalization | Yes vs No | Adjusted OR | 0.87 (0.52–1.46) |
| Amarin 2023 | history of asthma | All-cause hospitalization | Yes vs No | Adjusted OR | 3.96 (1.93–8.13) |
| Bahabri 2022 | smoking | Critical care unit | Yes vs No | Crude OR | 1.98 (0.18–12.65) |
| Bahabri 2022 | diabetes | Critical care unit | Yes vs No | Crude OR | 1 (0.3–3.35) |
| Bahabri 2022 | hypertension | Critical care unit | Yes vs No | Crude OR | 1.67 (0.49–6.67) |
| Bahabri 2022 | hypothyroidism | Critical care unit | Yes vs No | Crude OR | 1.36 (0.22–6.01) |
| Bahabri 2022 | heart failure | Critical care unit | Yes vs No | Crude OR | 2.09 (0.62–7.11) |
| Bahabri 2022 | cerebrovascular accident | Critical care unit | Yes vs No | Crude OR | 0.57 (0.06–2.87) |
| Bahabri 2022 | chronic kidney disease | Critical care unit | Yes vs No | Crude OR | 0.5 (0.05–2.49) |
| Bahabri 2022 | dialysis | Critical care unit | Yes vs No | Crude OR | 0 (0–3.35) |
| Bahabri 2022 | ischemic heart disease | Critical care unit | Yes vs No | Crude OR | 2.74 (0.71–9.86) |
| Bahabri 2022 | dyslipidemia | Critical care unit | Yes vs No | Crude OR | 0.64 (0.11–2.61) |
| Bahabri 2022 | chronic respiratory disease | Critical care unit | Yes vs No | Crude OR | 2.37 (0.71–8.66) |
| Bahabri 2022 | liver disease | Critical care unit | Yes vs No | Crude OR | 2.89 (0.05–58.91) |
| Bahabri 2022 | immunosuppression | Critical care unit | Yes vs No | Crude OR | 3.18 (0.46–17.29) |
| Bahabri 2022 | transplant | Critical care unit | Yes vs No | Crude OR | 2.89 (0.05–58.91) |
| Bahabri 2022 | bedbound before hospitalization | Critical care unit | Yes vs No | Crude OR | 0.35 (0.04–1.72) |
| Baillie 2020 | age >12 months | LRTI | age <= 12 months | Crude OR | 0.99 (0.63–1.55) |
| Baillie 2020 | HIV | LRTI | Yes vs No | Adjusted OR | 1.65 (0.85–3.28) |
| Baillie 2020 | HIV exposed uninfected | LRTI | Yes vs No | Adjusted OR | 1.43 (0.91–2.25) |
| Baillie 2020 | never breastfed | LRTI | Yes vs No | Adjusted OR | 0.8 (0.52–1.23) |
| Baillie 2020 | underweight | LRTI | Yes vs No | Adjusted OR | 3.53 (1.22–12.48) |
| Baillie 2020 | Day care attendance | LRTI | Yes vs No | Adjusted OR | 0.88 (0.49–1.58) |
| Baillie 2020 | Smoker in household | LRTI | Yes vs No | Adjusted OR | 1.38 (0.9–2.11) |
| Bruning 2015 | >1 comorbidity | Critical care unit | no comorbidity | Crude OR | 0.94 (0.13–5.16) |
| Bruning 2015 | prematurity | Critical care unit | Yes vs No | Crude OR | 1 (0.29–3.21) |
| Bruning 2015 | cardiac comorbidity | Critical care unit | Yes vs No | Crude OR | 1 (0.02–20.08) |
| Bruning 2015 | pulmonary comorbidity | Critical care unit | Yes vs No | Crude OR | 1 (0.02–20.08) |
| Bruning 2015 | renal comorbidity | Critical care unit | Yes vs No | Crude OR | 1 (0.02–20.08) |
| Bruning 2015 | genetic disorder | Critical care unit | Yes vs No | Crude OR | 1.64 (0.41–6.24) |
| Choi 2015 | immunosuppressant use | All-cause mortality | Yes vs No | Adjusted OR | 6.62 (2.37–19.17) |
| Chu 2016 | age <1 months | LRTI | Yes vs No | Crude OR | 1.52 (0.6–4.16) |
| Chu 2016 | age 1-6 months | LRTI | Yes vs No | Crude OR | 0.69 (0.45–1.06) |
| Chu 2016 | age 7-12 months | LRTI | Yes vs No | Crude OR | 1.14 (0.67–1.96) |
| Chu 2016 | age 13-<36 months | LRTI | Yes vs No | Crude OR | 1.17 (0.78–1.74) |
| Chu 2016 | White | LRTI | Yes vs No | Crude OR | 1.23 (0.83–1.83) |
| Chu 2016 | Black | LRTI | Yes vs No | Crude OR | 0.85 (0.41–1.77) |
| Chu 2016 | Asian | LRTI | Yes vs No | Crude OR | 1.08 (0.47–2.63) |
| Chu 2016 | other race | LRTI | Yes vs No | Crude OR | 0.84 (0.56–1.25) |
| Chu 2016 | Hispanic | LRTI | Yes vs No | Crude OR | 0.99 (0.65–1.52) |
| Chu 2016 | not Hispanic | LRTI | Yes vs No | Crude OR | 0.92 (0.58–1.47) |
| Chu 2016 | Unknown ethnicity | LRTI | Yes vs No | Crude OR | 1.29 (0.57–3.07) |
| Chu 2016 | malignancy | LRTI | Yes vs No | Crude OR | 1.76 (0.28–18.65) |
| Chu 2016 | solid organ transplant | LRTI | Yes vs No | Crude OR | 0.7 (0.05–9.69) |
| Chu 2016 | primary immunodeficiency | LRTI | Yes vs No | Crude OR | 0.35 (0.01–6.72) |
| Chu 2016 | cardiac comorbidity | LRTI | Yes vs No | Crude OR | 2 (0.83–5.33) |
| Chu 2016 | hematologic comorbidity | LRTI | Yes vs No | Crude OR | 0.7 (0.05–9.69) |
| Chu 2016 | genetic/metabolic comorbidity | LRTI | Yes vs No | Crude OR | 1.76 (0.28–18.65) |
| Chu 2016 | neurologic comorbidity | LRTI | Yes vs No | Crude OR | 0.8 (0.34–1.96) |
| Chu 2016 | gastrointestinal comorbidity | LRTI | Yes vs No | Crude OR | 0.7 (0.09–5.25) |
| Chu 2016 | other comorbidity | LRTI | Yes vs No | Crude OR | 1.57 (0.94–2.69) |
| Comte 2020 | age <=2 years | LRTI | age >2 years | Crude OR | 0.06 (0–0.54) |
| Comte 2020 | age <=2 years | LRTI | age >2 years | Crude OR | 1.73 (0.81–3.87) |
| Corne 2002 | asthma | LRTI | Yes vs No | Crude OR | 3.47 (0.84–17.78) |
| Esposito 2012a | age 12-48 months | LRTI | age <12 or >48 months | Crude OR | 1.13 (0.59–2.16) |
| Esposito 2012a | age >48 months | LRTI | age <=48 months | Crude OR | 0 (0–1.48) |
| Esposito 2012a | age <12 months | CAP | age >=12 months | Crude OR | 0.69 (0.37–1.29) |
| Esposito 2012a | age 12-48 months | CAP | age <12 or >48 months | Crude OR | 1.14 (0.61–2.13) |
| Esposito 2012a | age >48 months | CAP | age <=48 months | Crude OR | Inf (0.97–Inf) |
| Galindo-Fraga 2013 | age <18 years | All-cause hospitalization | age >=18 years | Crude OR | 0.78 (0.32–1.97) |
| Galindo-Fraga 2013 | age 18-59 years | All-cause hospitalization | age <18 or >=60 years | Crude OR | 0.6 (0.29–1.25) |
| Galindo-Fraga 2013 | age >=60 years | All-cause hospitalization | age <60 years | Crude OR | 3 (1.11–9.55) |
| Galindo-Fraga 2013 | age <18 years | All-cause mortality | age >=18 years | Crude OR | 0 (0–1.78) |
| Galindo-Fraga 2013 | age 18-59 years | All-cause mortality | age <18 or >=60 years | Crude OR | 1.07 (0.26–5.22) |
| Galindo-Fraga 2013 | age >=60 years | All-cause mortality | age <60 years | Crude OR | 2.47 (0.5–10.54) |
| Goka 2015 | age <=5 years | All-cause hospitalization | age >5 years | Adjusted OR | 1.45 (1.18–1.77) |
| Goka 2015 | age <=5 years | All-cause mortality or critical care unit | age >5 years | Adjusted OR | 2.14 (1.53–3.02) |
| Hung 2017 | male sex | All-cause mortality | female sex | Crude OR | 0.83 (0.55–1.24) |
| Hung 2017 | elderly home resident | All-cause mortality | Yes vs No | Adjusted OR | 4.59 (3.02–7.04) |
| Hung 2017 | pulmonary diseases | All-cause mortality | Yes vs No | Crude OR | 1.76 (1.12–2.74) |
| Hung 2017 | cardiovascular diseases | All-cause mortality | Yes vs No | Crude OR | 1.12 (0.68–1.8) |
| Hung 2017 | smoker | All-cause mortality | Yes vs No | Crude OR | 1.21 (0.74–1.93) |
| Hung 2017 | influenza vaccination | All-cause mortality | Yes vs No | Crude OR | 0.71 (0.28–1.56) |
| Hung 2017 | pneumococcal vaccination | All-cause mortality | Yes vs No | Crude OR | 1.3 (0.7–2.32) |
| Hung 2019 | age >= 18 years | Critical care unit | Yes vs No | Adjusted OR | 10.2 (2.06–100.51) |
| Hung 2019 | age >= 18 years | LRTI | age < 18 years | Crude OR | 3.73 (0.9–22.49) |
| Hung 2019 | age >= 18 years | LRTI | age < 18 years | Crude OR | 0 (0–2.59) |
| Hung 2019 | age >= 18 years | LRTI | age < 18 years | Crude OR | 0.27 (0.01–2.13) |
| Hung 2019 | age >= 18 years | LRTI | age < 18 years | Crude OR | 10.42 (2.62–52.03) |
| Hung 2019 | age >= 18 years | Critical care unit | Yes vs No | Crude OR | 1.64 (0.21–19.62) |
| Hung 2019 | age >= 18 years | LRTI | age < 18 years | Crude OR | 8.18 (1.01–102.36) |
| Jacobs 2013 | COPD or asthma | LRTI | Yes vs No | Adjusted OR | 0.64 (0.05–4.92) |
| Jacobs 2013 | tobacco use ever | LRTI | Yes vs No | Crude OR | 1.12 (0.36–3.44) |
| Jacobs 2013 | diabetes | LRTI | Yes vs No | Crude OR | 0.87 (0.16–4.18) |
| Jacobs 2013 | COPD or asthma | LRTI | Yes vs No | Crude OR | 0.04 (0–0.26) |
| Lee 2021 | solid organ transplant | All-cause mortality | Yes vs No | Crude OR | 0.38 (0.12–1.07) |
| Lee 2021 | hematopoietic stem cell transplant | All-cause mortality | Yes vs No | Crude OR | 0.76 (0.15–3.16) |
| Lee 2021 | non-transplant critically ill patients in ICU | All-cause mortality | Yes vs No | Crude OR | 2.76 (1.04–7.66) |
| Perez 2015 | bronchopulmonary dysplasia | Critical care unit | Yes vs No | Adjusted OR | 1.65 (0.5–7.6) |
| Pierangeli 2011 | age >65 years | LRTI | age 18-65 years | Crude OR | Inf (0.25–Inf) |
| Pierangeli 2011 | age >65 years | All-cause hospitalization | age 18-65 years | Crude OR | 36.06 (2.59–2306.93) |
| Piralla 2012 | transplant within 1-3 months | LRTI | immunocompetent | Adjusted OR | 0.13 (0.03–0.42) |
| Piralla 2012 | transplant greater than 3 months | LRTI | immunocompetent | Adjusted OR | 0.2 (0.09–0.44) |
| Piralla 2012 | oncohematologic | LRTI | immunocompetent | Adjusted OR | 0.36 (0.17–0.75) |
| Piralla 2012 | age <5 years | LRTI | age >5 years | Adjusted OR | 4.24 (2.46–7.42) |
| Sanchez-Codez 2021 | age | Critical care unit | per 1 year increase | Crude OR | 1.07 (1.05–1.09) |
| Song 2023 | rs3887998 | LRTI | Yes vs No | Crude OR | 1.01 (0.82–1.24) |
| Song 2023 | rs140154310 | LRTI | Yes vs No | Crude OR | 2.06 (0.97–4.37) |
| Song 2023 | rs73195657 | LRTI | Yes vs No | Crude OR | 1.45 (0.97–2.17) |
| Song 2023 | rs146004234 | LRTI | Yes vs No | Crude OR | 0.87 (0.5–1.5) |
| Song 2023 | rs4730125 | LRTI | Yes vs No | Crude OR | 0.93 (0.76–1.14) |
| Song 2023 | rs6967330 | LRTI | Yes vs No | Crude OR | 1.2 (0.89–1.62) |
| Song 2023 | rs73195665 | LRTI | Yes vs No | Crude OR | 1.17 (0.78–1.75) |
| Song 2023 | rs408223 | LRTI | Yes vs No | Crude OR | 0.93 (0.69–1.25) |
| Song 2023 | rs3887998_rs73195657 AT haplotype | LRTI | rs3887998_rs73195657 GT haplotype | Crude OR | 0.88 (0.7–1.11) |
| Song 2023 | rs3887998_rs73195657 AC haplotype | LRTI | rs3887998_rs73195657 GT haplotype | Crude OR | 1.55 (1.02–2.37) |
| Song 2023 | rs4730125_rs6967330_ rs73195665 TGG haplotype | LRTI | rs4730125_rs6967330_ rs73195665 GGG haplotype | Crude OR | 0.96 (0.78–1.18) |
| Song 2023 | rs4730125_rs6967330_ rs73195665 GAA haplotype | LRTI | rs4730125_rs6967330_ rs73195665 GGG haplotype | Crude OR | 1.15 (0.76–1.76) |
| Song 2023 | rs4730125_rs6967330_ rs73195665 GAG haplotype | LRTI | rs4730125_rs6967330_ rs73195665 GGG haplotype | Crude OR | 1.14 (0.75–1.73) |
| To 2016 | >=1 comorbidity | All-cause mortality | Yes vs No | Crude OR | Inf (0.04–Inf) |
| To 2016 | atrial fibrillation | All-cause mortality | Yes vs No | Crude OR | 2.52 (0.03–65.76) |
| To 2016 | lung carcinoma | All-cause mortality | Yes vs No | Crude OR | Inf (0.12–Inf) |
| To 2016 | prostate carcinoma | All-cause mortality | Yes vs No | Crude OR | Inf (0.12–Inf) |
| To 2016 | COPD | All-cause mortality | Yes vs No | Crude OR | 5.05 (0.05–467.89) |
| To 2016 | end stage renal failure | All-cause mortality | Yes vs No | Crude OR | 0 (0–175.02) |
| To 2016 | hypertension | All-cause mortality | Yes vs No | Crude OR | 1.93 (0.11–33.17) |
| To 2016 | ischemic heart disease | All-cause mortality | Yes vs No | Crude OR | 0 (0–175.02) |
| To 2016 | obstructive sleep apnea | All-cause mortality | Yes vs No | Crude OR | 0 (0–175.02) |
| To 2016 | peripheral vascular disease | All-cause mortality | Yes vs No | Crude OR | 0 (0–175.02) |
| To 2016 | systemic lupus erythematosus | All-cause mortality | Yes vs No | Crude OR | 0 (0–175.02) |
| To 2016 | transient ischemic attack | All-cause mortality | Yes vs No | Crude OR | 0 (0–175.02) |
| To 2016 | cirrhosis | All-cause mortality | Yes vs No | Crude OR | 5.05 (0.05–467.89) |
| To 2016 | asthma | All-cause mortality | Yes vs No | Crude OR | 0 (0–12.4) |
| To 2016 | fatty liver | All-cause mortality | Yes vs No | Crude OR | 0 (0–26.1) |
| To 2016 | epilepsy | All-cause mortality | Yes vs No | Crude OR | 0 (0–175.02) |
| To 2016 | bronchiectasis | All-cause mortality | Yes vs No | Crude OR | 0 (0–175.02) |
| To 2016 | lymphoma | All-cause mortality | Yes vs No | Crude OR | 0 (0–175.02) |
| To 2016 | hyperlipidemia | All-cause mortality | Yes vs No | Crude OR | 0 (0–12.4) |
| To 2016 | hyperparathyroidism | All-cause mortality | Yes vs No | Crude OR | Inf (0.12–Inf) |
| To 2016 | bronchiolitis obliterans organizing pneumonia | All-cause mortality | Yes vs No | Crude OR | 0 (0–175.02) |
| To 2016 | diabetic nephropathy | All-cause mortality | Yes vs No | Crude OR | 0 (0–175.02) |
| To 2016 | cardiomyopathy | All-cause mortality | Yes vs No | Crude OR | Inf (0.12–Inf) |
| To 2016 | cholecystectomy | All-cause mortality | Yes vs No | Crude OR | Inf (0.12–Inf) |
| To 2016 | renal impairment | All-cause mortality | Yes vs No | Crude OR | Inf (0.12–Inf) |
| To 2016 | biliary atresia | All-cause mortality | Yes vs No | Crude OR | Inf (0.12–Inf) |
| To 2016 | liver transplant | All-cause mortality | Yes vs No | Crude OR | Inf (0.12–Inf) |
| To 2016 | gout | All-cause mortality | Yes vs No | Crude OR | 0 (0–175.02) |
| To 2016 | eczema | All-cause mortality | Yes vs No | Crude OR | 0 (0–175.02) |
| To 2016 | multiple myeloma | All-cause mortality | Yes vs No | Crude OR | 0 (0–175.02) |
| To 2016 | hypopituitarism | All-cause mortality | Yes vs No | Crude OR | 0 (0–175.02) |
| To 2016 | obesity | All-cause mortality | Yes vs No | Crude OR | 0 (0–175.02) |
| To 2016 | germ cell tumor | All-cause mortality | Yes vs No | Crude OR | 0 (0–175.02) |
| Waghmare 2019 | age 21-60 years at transplant | LRTI | age <21 years at transplant | Crude HR | 0.82 (0.51–1.33) |
| Waghmare 2019 | age 61+ years at transplant | LRTI | age <21 years at transplant | Crude HR | 1.25 (0.72–2.17) |
| Waghmare 2019 | male sex | LRTI | female sex | Crude HR | 1.16 (0.77–1.74) |
| Waghmare 2019 | transplant year 2022-2015 | LRTI | transplant year 1992-2010 | Crude HR | 0.91 (0.61–1.36) |
| Waghmare 2019 | white race | LRTI | nonwhite race | Crude HR | 1.12 (0.7–1.78) |
| Waghmare 2019 | unknown race | LRTI | nonwhite race | Crude HR | 0.72 (0.28–1.9) |
| Waghmare 2019 | HCT cells from bone marrow/cord | LRTI | HCT cells from peripheral blood stem cells | Crude HR | 1.35 (0.9–2.04) |
| Waghmare 2019 | non-myeloablative conditioning regimen | LRTI | myeloablative ± TBI conditioning regimen | Crude HR | 1.68 (1.11–2.54) |
| Waghmare 2019 | % FEV1/FVC >70 | LRTI | Yes vs No | Crude HR | 0.85 (0.49–1.47) |
| Waghmare 2019 | % TLC >80 | LRTI | Yes vs No | Crude HR | 1.24 (0.63–2.41) |
| Waghmare 2019 | white blood cell count <=1000x10E6 cells/L | LRTI | Yes vs No | Crude HR | 1.39 (0.78–2.49) |
| Waghmare 2019 | neutrophil count <=100x10E6 cells/L | LRTI | Yes vs No | Crude HR | 1.91 (1.04–3.49) |
| Waghmare 2019 | monocyte count <=100x10E6 cells/L | LRTI | Yes vs No | Crude HR | 2.01 (1.24–3.26) |
| Waghmare 2019 | platelet count <10000x10E6 cells/L | LRTI | Yes vs No | Crude HR | 1.55 (0.38–6.3) |
| Waghmare 2019 | IVIG given | LRTI | Yes vs No | Crude HR | 1.42 (0.72–2.82) |
| Waghmare 2019 | time to URTI from transplant 101-365 days | LRTI | time to URTI from transplant <=100 days | Crude HR | 1.42 (0.89–2.28) |
| Waghmare 2019 | time to URTI from transplant >365 days | LRTI | time to URTI from transplant <=100 days | Crude HR | 0.8 (0.5–1.29) |
| Waghmare 2019 | HCT-CI score >=3 | LRTI | HCT-CI score 0-2 | Crude HR | 0.7 (0.47–1.06) |
| Waghmare 2019 | missing HCT-CI score | LRTI | HCT-CI score 0-2 | Crude HR | 0.41 (0.06–2.94) |
| Waghmare 2019 | donor statin use | LRTI | no donor statin use | Crude HR | 1.16 (0.28–4.9) |
| Waghmare 2019 | unknown donor statin use | LRTI | no donor statin use | Crude HR | 0.81 (0.51–1.28) |
| Waghmare 2019 | acute GVHD as time-dependent grades II-IV | LRTI | acute GVHD as time-dependent grades 0-I | Crude HR | 1.52 (1.01–2.28) |
| Waghmare 2019 | chronic GVHD as time-dependent | LRTI | Yes vs No | Crude HR | 1.18 (0.79–1.75) |
| Waghmare 2019 | donor CMV serostatus + | LRTI | Yes vs No | Crude HR | 1.22 (0.82–1.83) |
| Waghmare 2019 | allogeneic/unrelated HCT donor | LRTI | autologous HCT donor | Crude HR | 2.08 (1.22–3.58) |
| Waghmare 2019 | lymphocyte count <=100x10E6 cells/L | LRTI | lymphocyte count >100x10E6 cells/L | Crude HR | 2.39 (1.33–4.29) |
| Waghmare 2019 | steroid use >=1 to <2 mg/kg/day | LRTI | steroid use 0 to <1 mg/kg/day | Crude HR | 1.73 (0.84–3.58) |
| Waghmare 2019 | steroid use >=2 mg/kg/day | LRTI | steroid use 0 to <1 mg/kg/day | Crude HR | 2.91 (1.27–6.69) |
| Waghmare 2019 | any previous HRV event | LRTI | Yes vs No | Crude HR | 1.67 (1.05–2.65) |
| Waghmare 2019 | albumin <=3 g/dL | LRTI | Yes vs No | Crude HR | 1.65 (0.99–2.76) |
| Waghmare 2019 | recipient statin use | LRTI | Yes vs No | Crude HR | 2.06 (1.16–3.64) |
| Waghmare 2019 | recipient CMV serostatus + | LRTI | Yes vs No | Crude HR | 1.79 (1.17–2.73) |
| Waghmare 2019 | transplant year 2022-2015 | LRTI | transplant year 1992-2010 | Crude HR | 1.07 (0.52–2.2) |
| Waghmare 2019 | white race | LRTI | nonwhite race | Crude HR | 1.92 (0.73–5.01) |
| Waghmare 2019 | unknown race | LRTI | nonwhite race | Crude HR | 0.71 (0.08–6.08) |
| Waghmare 2019 | HCT cells from bone marrow/cord | LRTI | HCT cells from peripheral blood stem cells | Crude HR | 0.85 (0.38–1.9) |
| Waghmare 2019 | allogeneic/unrelated HCT donor | LRTI | autologous HCT donor | Crude HR | 2.37 (0.83–6.78) |
| Waghmare 2019 | age 21-60 years at transplant | LRTI | age <21 years at transplant | Crude HR | 1.95 (0.66–5.76) |
| Waghmare 2019 | age 61+ years at transplant | LRTI | age <21 years at transplant | Crude HR | 2.76 (0.85–8.96) |
| Waghmare 2019 | male sex | LRTI | female sex | Crude HR | 0.94 (0.46–1.91) |
| Waghmare 2019 | % FEV1/FVC >70 | LRTI | Yes vs No | Crude HR | 0.71 (0.29–1.76) |
| Waghmare 2019 | % TLC >80 | LRTI | Yes vs No | Crude HR | 1.06 (0.36–3.09) |
| Waghmare 2019 | white blood cell count <=1000x10E6 cells/L | LRTI | Yes vs No | Crude HR | 2.15 (0.88–5.24) |
| Waghmare 2019 | lymphocyte count <=100x10E6 cells/L | LRTI | lymphocyte count >100x10E6 cells/L | Crude HR | 1.87 (0.72–4.87) |
| Waghmare 2019 | neutrophil count <=100x10E6 cells/L | LRTI | Yes vs No | Crude HR | 2.47 (0.95–6.43) |
| Waghmare 2019 | IVIG given | LRTI | Yes vs No | Crude HR | 0.94 (0.23–3.95) |
| Waghmare 2019 | any previous HRV event | LRTI | Yes vs No | Crude HR | 0.36 (0.11–1.17) |
| Waghmare 2019 | time to URTI from transplant 101-365 days | LRTI | time to URTI from transplant <=100 days | Crude HR | 1.17 (0.52–2.64) |
| Waghmare 2019 | time to URTI from transplant >365 days | LRTI | time to URTI from transplant <=100 days | Crude HR | 0.46 (0.18–1.18) |
| Waghmare 2019 | HCT-CI score >=3 | LRTI | HCT-CI score 0-2 | Crude HR | 0.81 (0.38–1.72) |
| Waghmare 2019 | missing HCT-CI score | LRTI | HCT-CI score 0-2 | Crude HR | 3.05 (0.71–13.1) |
| Waghmare 2019 | donor statin use | LRTI | no donor statin use | Crude HR | 2.34 (0.29–19) |
| Waghmare 2019 | unknown donor statin use | LRTI | no donor statin use | Crude HR | 1.1 (0.46–2.61) |
| Waghmare 2019 | recipient statin use | LRTI | Yes vs No | Crude HR | 1.74 (0.67–4.53) |
| Waghmare 2019 | acute GVHD as time-dependent grades II-IV | LRTI | acute GVHD as time-dependent grades 0-I | Crude HR | 1.09 (0.54–2.21) |
| Waghmare 2019 | chronic GVHD as time-dependent | LRTI | Yes vs No | Crude HR | 0.84 (0.41–1.73) |
| Waghmare 2019 | recipient CMV serostatus + | LRTI | Yes vs No | Crude HR | 2.1 (0.97–4.56) |
| Waghmare 2019 | donor CMV serostatus + | LRTI | Yes vs No | Crude HR | 0.86 (0.41–1.83) |
| Waghmare 2019 | albumin <=3 g/dL | LRTI | Yes vs No | Crude HR | 3.21 (1.48–6.94) |
| Waghmare 2019 | non-myeloablative conditioning regimen | LRTI | myeloablative ± TBI conditioning regimen | Crude HR | 2.24 (1.08–4.63) |
| Waghmare 2019 | monocyte count <=100x10E6 cells/L | LRTI | Yes vs No | Crude HR | 2.28 (1.01–5.15) |
| Waghmare 2019 | albumin <=3 g/dL | LRTI | Yes vs No | Crude HR | 2.84 (1.3–6.21) |
| Waghmare 2019 | monocyte count <=100x10E6 cells/L | LRTI | Yes vs No | Crude HR | 2.33 (1.02–5.31) |
| Waghmare 2019 | steroid use >=1 to <2 mg/kg/day | LRTI | steroid use 0 to <1 mg/kg/day | Crude HR | 3.17 (1.09–9.2) |
| Waghmare 2019 | steroid use >=2 mg/kg/day | LRTI | steroid use 0 to <1 mg/kg/day | Crude HR | 4.04 (1.2–13.6) |

Note: LRTI = lower respiratory tract infection; OR = odds ratio; HR = hazard ratio. Some studies have multiple entries for the same outcome because they reported subgroups of each outcome (e.g., LRTI could include bronchitis and bronchiolitis assessed as distinct outcomes).

**Table S6.** Newcastle-Ottawa Scale risk of bias assessments for the included cohort studies (*n* = 25)

| Study | Selection | | | | Comparability | Outcome | | | Total Score |
| --- | --- | --- | --- | --- | --- | --- | --- | --- | --- |
|  | Representativeness of exposed cohort | Selection of non-exposed cohort | Ascertainment of exposure | Demonstration that outcome of interest was not present at start of study | Comparability of cohorts | Assessment of outcome | Was follow-up long enough | Adequacy of follow-up |  |
| Amarin 2023 | A | A | A | A | A | B | A | A | 9 |
| Bahabri 2022 | A | A | A | A | C | B | A | A | 7 |
| Bruning 2015 | B | A | A | A | C | B | A | A | 6 |
| Cherry 1967 | C | A | A | A | C | B | A | D | 5 |
| Cheuk 2007 | B | A | A | A | C | B | A | A | 7 |
| Choi 2015 | A | A | A | A | C | B | A | A | 7 |
| Chu 2016 | B | A | A | A | A | B | A | A | 9 |
| Comte 2020 | B | A | A | A | A | B | A | A | 8 |
| Corne 2002 | B | A | A | A | C | B | A | A | 8 |
| Esposito 2012 | B | A | A | A | C | B | A | A | 7 |
| Galindo-Fraga 2013 | B | A | A | A | C | B | A | A | 7 |
| Garcia-Garcia 2015 | B | A | A | A | C | B | A | A | 7 |
| Gerna 2009 | No | A | A | A | C | B | A | A | 6 |
| Hung 2017 | A | A | A | A | B | B | A | A | 8 |
| Hung 2019 | B | A | A | A | B | B | A | A | 8 |
| Jacobs 2013 | B | A | A | A | B | B | A | A | 8 |
| Kellner 1988 | B | A | A | A | C | B | A | A | 7 |
| Kim 2019 | A | A | A | A | C | B | A | A | 7 |
| Lee 2021 | C | A | A | A | C | B | A | A | 6 |
| Nicholson 1996 | C | A | A | A | A | B | A | A | 8 |
| Perez 2015 | C | A | A | A | B | B | A | A | 7 |
| Pierangeli 2011 | B | A | A | A | C | B | A | A | 7 |
| Piralla 2012 | B | A | A | A | A | B | A | A | 9 |
| To 2016 | C | A | A | A | C | B | A | A | 6 |
| Waghmare 2019 | B | A | A | A | B | B | A | B | 8 |

**Table S7.** Newcastle-Ottawa Scale risk of bias assessments for the included case-control study (*n* = 1).

| Study | Selection | | | | Comparability | Outcome | | | Total Score |
| --- | --- | --- | --- | --- | --- | --- | --- | --- | --- |
|  | Case definition adequate | Representativeness of cases | Selection of controls | definition of controls | Comparability of cases and controls | Ascertainment of exposure | Same method of ascertainment | Non-response rate |  |
| Baillie 2020 | A | A | A | A | A | A | A | A | 9 |

**Table S8.** Newcastle-Ottawa Scale risk of bias assessments for the included cross-sectional studies (*n* = 3).

| Study | Selection | | | | Comparability | Outcome | | Total Score |
| --- | --- | --- | --- | --- | --- | --- | --- | --- |
|  | Representativeness of sample | Sample size | Non-respondents/missing data | Ascertainmen of the exposure | Comparability of cohorts | Assessment of outcome | Statistical test |  |
| Goka 2015 | A | A | A | A | A | B | A | 9 |
| Sanchez-Codez 2021 | B | A | A | A | A | A | A | 9 |
| Song 2023 | B | A | A | A | C | B | A | 7 |

# Result S1. Citations of excluded full-text studies.

**1. Conference abstract (n = 101)**

1. Abdul Ghani A, Morrow B, Hardie D, Argent A. An investigation into the prevalence and outcome of patients admitted to a Paediatric Intensive Care Unit (PICU) with viral respiratory tract infections in Cape Town, South Africa. Pediatric Critical Care Medicine. 2011;12(3 SUPPL. 1):A53.
2. Ahrens J, Morrow B, Argent A. Pandemic influenza a H1N1 (2009) in critically ill children admitted to a paediatric intensive care unit, South Africa. Pediatric Critical Care Medicine. 2011;12(3 SUPPL. 1):A124.
3. Al-Hajjar S, Al-Ahmed O, Al-Thawadi S. Rhinovirus infections in high-risk children. Archives of Disease in Childhood. 2012;97(SUPPL. 2):A265.
4. Aliaga FA, Olivares F, Appiani F, Farias P, Alberto F, Hernandez A. Viral induce acute respiratory distress syndrome (ARDS): Characterization of patients in a tertiary hospital in South America. Intensive Care Medicine Experimental. 2016;4(Supplement 1).
5. Alvarez AE, De Lima Marson FA, Santiago J, Bertuzzo CS, Arns CW, Ribeiro JD. Severe acute viral bronchiolitis in infants: Clinical features, etiology and outcomes. Pediatric Pulmonology. 2016;51(Supplement 42):S14.
6. Annamalay AA, Abbott S, Bizzintino J, Khoo SK, Green R, Le Souef PN. Role of human rhinovirus in acute lower respiratory infections in HIV-infected and HIV-uninfected South African children. American Journal of Respiratory and Critical Care Medicine. 2013;187(MeetingAbstracts).
7. Ayyash A, DeFelice N, Zebrowski A, Mei C, Patel A, Thanik E. A study of the environmental drivers on asthma in pediatric patients during the COVID-19 pandemic. Journal of Allergy and Clinical Immunology. 2024;153(2 Supplement):AB181.
8. Baalachandran R, Laroche D, Ghazala L, Carr GE. Predictors of mortality in patients admitted to an intensive care unit with viral pneumonia. American Journal of Respiratory and Critical Care Medicine. 2015;191(MeetingAbstracts).
9. Castejon-Ramirez S, Chaisavaneeyajirn S, Ferrolino JA, Allison KJ, Peterson M, Dallas RH, et al. Clinical Outcomes of Human Rhinovirus Infections in Pediatric Hemopoietic Stem Cell Transplant Patients. Journal of the Pediatric Infectious Diseases Society. 2023;12(Supplement 1):S21-S3.
10. Chen MIC, Jiang L, Lee VJ, Lim WY, Lin RTP, Cui L, et al. Difference in distribution of viral respiratory pathogens in acute respiratory illness (ARI) between a prospective community cohort and inpatients in Singapore. Annals of the Academy of Medicine Singapore. 2015;44(10 SUPPL. 1):S346.
11. Clark T, Nicholson K, Medina MJ. The burden of respiratory virus infection in hospitalised adult patients with acute respiratory illnesscategory: Scientific free paper. Journal of Infection. 2011;63(6):e23-e4.
12. Cox DW, Khoo SK, Bizzintino J, Ferrari G, Zhang G, Lee WM, et al. Young children presenting to an emergency department with an acute lower respiratory illness due to human rhinovirus have increased respiratory admissions to hospital. American Journal of Respiratory and Critical Care Medicine. 2012;185(MeetingAbstracts).
13. Crossman H, Barron M, Levi M. Characteristics of human rhinovirus (HRV) infections in transplant recipients. American Journal of Transplantation. 2016;16(Supplement 3):696-7.
14. Davis PG, Khoo SK, Cox DW, Bizzintino J, Lee W, Geelhoed GC, et al. Human rhinovirus (HRV)-C is as common in children with HRV who required emergency treatment for an acute respiratory illness as symptomatic sibling controls. American Journal of Respiratory and Critical Care Medicine. 2011;183(1 MeetingAbstracts).
15. De Souza CCT, Chacorowski ARP, Vituri SC. Evaluation of respiratory viruses in children hospitalized for bronchiolitis. Pediatric Pulmonology. 2016;51(Supplement 42):S34-S5.
16. Delgado-Corcoran C. Rhinovirus infecion in pediatric patients undergoing heart surgery. Pediatric Critical Care Medicine. 2013;14(5 SUPPL. 1):S113.
17. Dugan C, Calderwood C, Monk E, McCann N, Brown J, Lipman M, et al. Respiratory viral pathogens and associated hospital admissions in HIV-positive adults and patients with obstructive lung disease. HIV Medicine. 2017;18(Supplement 1):45.
18. Ferguson P, Gilroy N, Mackay I, Sloots T, Nissen M, Sorrell T. Human rhinovirus-C: A frequently detected species in adult haematopoietic stem cell transplant recipients with lower respiratory tract infection. Clinical Microbiology and Infection. 2011;17(SUPPL. 4):S665.
19. Fernandez R, Melon S, Fernandez C, Palomo P, Gonzalez AJ, Alonso E, et al. Respiratory viral infections after HSCT. Bone Marrow Transplantation. 2012;47(SUPPL. 1):S23.
20. Gaddam S, Kancharla A, Jayaraman D, Venkatramanan P, Madhuravasal Krishnan J, Dhanurekha L, et al. Profile of respiratory viral infections in pediatric hematology and oncology unit - Prospective study from a tertiary care center in South India. Pediatric Hematology Oncology Journal. 2023;8(4 Supplement):S45-S6.
21. Gomes DN, Alfaiate A, Clerigo V, Fernandes L, Castanho M, Sousa S, et al. Late Breaking Abstract - Co-infections, an unexplored branch in the COVID-19 pandemic. European Respiratory Journal. 2020;56(Supplement 64).
22. Harris L, Allcock R, Kresoje N, Le Souef P, Laing I. Genetic variants in human respiratory virus receptors were associated with infection, recovery and recurrence of wheezing exacerbations in children. Respirology. 2018;23(Supplement 1):26.
23. Harris L, Khoo K, Franks K, Prastanti F, Oo S, Everard J, et al. IFITM3 variants were associated with rhinovirus (RV) species a infection and recurrent hospital visits in children with acute lower respiratory illness. Respirology. 2016;21(SUPPL. 2):122.
24. Hasan L, Crescencio JCR, Jenkins M, Burgess MJ. Human Rhinovirus Infection in Multiple Myeloma Patients: Effect on Morbidity and Mortality. Open Forum Infectious Diseases. 2021;8(SUPPL 1):S752.
25. Henningfeld J, Xia N, Gambrell-Sanders D, Zhang L, Yan K, Steuart R. Respiratory-Related Investigations Following Pediatric Decannulation. American Journal of Respiratory and Critical Care Medicine. 2023;207(1).
26. Hill H, Kemper S, Maupin K. Comparing rhinovirus pneumonia, bacterial pneumonia and rhinovirus and bacterial pneumonia infections in adults at Charleston area medical center. Chest. 2016;150(4 Supplement 1):146A.
27. Holmdahl I, Filiou A, Asarnoj A, Van Hage M, Borres MP, Stenberg Hammar K, et al. Early life wheeze-risk factors for asthma in school age. Allergy: European Journal of Allergy and Clinical Immunology. 2020;75(SUPPL 109):67-8.
28. Hsu C, Wang L, Maestri E, Jacob A, Mayo S, Wang XW, et al. VIRAL EXPOSURE PREDICTS MORBIDITY AND MORTALITY IN ALCOHOLASSOCIATED HEPATITIS. Hepatology. 2023;78(Supplement 1):S1671-S2.
29. Ieven M, Loens K, Coenjaerts F, Lammens C, Vanderstraeten A, Verheij T, et al. The role of respiratory viruses and M. pneumoniae in lower respiratory tract infections in primary care. Clinical Microbiology and Infection. 2009;15(S4):S27.
30. Imakita M, Shiraki K, Yutani C, Ishibashi-Ueda H. Pneumonia caused by rhinovirus. Clinical infectious diseases : an official publication of the Infectious Diseases Society of America. 2000;30(3):611-2.
31. Jones A, Helm J, Bright-Thomas R, Brennan A, Webb K, Mutton K. Is rhinovirus associated with exacerbations in adults with CF? Journal of Cystic Fibrosis. 2010;9(SUPPL. 1):S37.
32. Kalra S, Rebaza AP, Bermejo SD, Gomez JL, Dela Cruz C. Characteristics and outcomes of adults with multiple co-occurring respiratory viral infections. American Journal of Respiratory and Critical Care Medicine. 2020;201(1).
33. Kharosi ZA, Dildar B, Al-Maamari K, Al-Mubaihsi SM. Epidemiological and clinical characteristics of respiratory viral infections in adults at sultan qaboos university hospital (SQUH), in Oman. Respirology. 2017;22(Supplement 3):208-9.
34. Kim DR, Kim KR, Park H, Cho J, Huh HJ, Lee NY, et al. Children with severe human rhinovirus lower respiratory tract infection. Open Forum Infectious Diseases. 2022;9(Supplement 2):S891.
35. Kim SR, Dossetter BJ, Xie H, Leisenring WM, Cheng GS, Englund JA, et al. Supplemental Oxygen-Free Days in Human Rhinovirus Infections of the Lower Respiratory Tract in Hematopoietic Cell Transplant Recipients. Transplantation and Cellular Therapy. 2023;29(2 Supplement):S323.
36. Kloepfer K, Vrtis R, Pappas T, Kang T, Salazar L, Anderson EL, et al. Detection of streptococcus pneumoniae and human rhinovirus is associated with loss of asthma control. Journal of Allergy and Clinical Immunology. 2013;131(2 SUPPL. 1):AB233.
37. Kosma E, Hammargren M, Kilander CP. Bronchiolitis during the first year after birth in term and preterm infants. 2014;44.
38. Kraft CS, Jacob JT, Sears MH, Burd EM, Caliendo AM, Hill CE, et al. Clinical characteristics of respiratory infection in adults with enterovirus 68 (EV68). Clinical and Translational Science. 2012;5(2):146.
39. Kulkarni S, Michail S, Smith C, Tomlins J, Murray J, Dennis M, et al. Retrospective analysis of seasonal respiratory viral infections (SRVI) in hematology, lymphoma and oncology patients. Blood. 2016;128(22).
40. Kyo M, Zhu Z, Fujiogi M, Shibata R, Camargo CA, Hasegawa K. Respiratory Virus-specific Nasopharyngeal Lipidome Signatures and Severity in Infants Hospitalized for Bronchiolitis: A Prospective Multicenter Cohort Study. American Journal of Respiratory and Critical Care Medicine. 2023;207(1).
41. Leite S, Lachado A, Correia-Costa L, Fernandes A, Ramos A, Reis MG. Viral bronchiolitis and risk factors for severe outcome. Pediatric Pulmonology. 2017;52(Supplement 46):S171-S2.
42. Leung TF, Song YP, Tang MF, Tao KP, Leung ASY, Tsun JGS, et al. Longitudinal study of risk factors for asthma exacerbations in Chinese school children. Allergy: European Journal of Allergy and Clinical Immunology. 2019;74(Supplement 106):724-5.
43. Leung TF, Tse LY, Lam WY, Chan WC, Wong GWK, Chan PKS. Human rhinovirus is highly prevalent in Hong Kong children with wheezing illnesses. Paediatric Respiratory Reviews. 2012;13(Supplement 1):S55.
44. Linder-Jackson JE, Plachco TE, Bossi L, Bauer G, Polack FP, Libster RP, et al. Human rhinovirus bronchiolitis predominant in very low birthweight infants in Argentina. Journal of Allergy and Clinical Immunology. 2014;133(2 SUPPL. 1):AB192.
45. Longtin J, Winter A-L, Heng D, Marchand-Austin A, Eshaghi A, Patel S, et al. Severe human rhinovirus outbreak associated with fatalities in a long-term care facility in Ontario, Canada. Journal of the American Geriatrics Society. 2010;58(10):2036-8.
46. MacBean V, Drysdale S, Yarzi MN, Rafferty GF, Greenough A. Impact of respiratory viral infections on school age outcomes in prematurely born children. American Journal of Respiratory and Critical Care Medicine. 2017;195((MacBean, Yarzi, Rafferty, Greenough) King's College London, London, United Kingdom(Drysdale) Oxford University, Oxford, United Kingdom).
47. MacBean V, Lunt A, Drysdale S, Rafferty G, Greenough A. Predicting healthcare outcomes in prematurely born infants using cluster analysis. Archives of Disease in Childhood. 2017;102(Supplement 1):A190-A1.
48. Mahr T, Eppley J. Effects of omalizumab on rhinovirus infections, illnesses and exacerbations of asthma. Pediatrics. 2018;142(Supplement 4):S268-S9.
49. Mandelia Y, Procop GW, Richter SS, Worley S, Liu W, Esper F. Dynamics of respiratory viral co-infections: Predisposition for and clinical impact of viral pairings in children and adults. Open Forum Infectious Diseases. 2019;6(Supplement 2):S916-S7.
50. Mansbach JM, Piedra PA, Laham F, McAdam A, Clark S, Sullivan AF, et al. Nasopharyngeal aspirate lactate dehydrogenase levels predict bronchiolitis severity in a prospective multicenter emergency department study. Academic Emergency Medicine. 2011;18(5 SUPPL. 1):S84-S5.
51. Martin-Quiros A, Romero-Gomez M, Figueira J, Prados C, Martinez-Sanchez N, Mora-Rillo M, et al. Respiratory viruses other than influenza as cause of severely respiratory infections in adult patients admitted to an intensive care unit during 2009/2010 influenza season. Clinical Microbiology and Infection. 2012;18(SUPPL. 3):214.
52. Matz J. Vapendavir for the treatment of naturally acquired rhinovirus infection in asthmatic adults: effect on asthma control in a phase 2 clinical trial. 2013;187.
53. McCallum G, Grimwood K, Oguoma V, Leach A, Smith-Vaughan H, Versteegh L, et al. The point prevalence of respiratory syncytial virus in hospital and community-based studies in children from Northern Australia: Studies in a 'high-risk' population. European Respiratory Journal. 2019;54(Supplement 63).
54. Mikulska M, Del Bono V, Dini S, Raiola A, Dominietto A, Bregante S, et al. Respiratory viral infections after allogeneic haematopoietic stem cell transplantation. Clinical Microbiology and Infection. 2011;17(SUPPL. 4):S615.
55. Miller EK, Williams JV, Gebretsadik T, Carroll K, Dupont W, Mohamed Y, et al. Host and viral risk factors associated with human rhinovirus infant respiratory illness severity. Journal of Allergy and Clinical Immunology. 2010;125(2 SUPPL. 1):AB151.
56. Miller R, De Havenon A. Human rhinovirus lower respiratory tract infection: Morbidity and mortality in the ICU setting. Critical Care Medicine. 2011;39(SUPPL. 12):178.
57. Motoa G, Mendoza MA, Raja M, Simkins J, Anjan S, Martin E, et al. Rhinovirus Infection in Solid Organ Transplant Recipients. American Journal of Transplantation. 2022;22(Supplement 3):1067.
58. Murrell J, Hill T, State A, Francis S, Pickersgill L, Raza M, et al. Impact of societal and healthcare infection control measures on incidence of non-sars-2-cov respiratory viral infections in HSCT in-patient admissions; a single uk centre experience. Bone Marrow Transplantation. 2022;57(Supplement 1):272-3.
59. Naidu G, Wainwright R, MacKinnon D, Poiyadjis S, Rowe B, Nunes M, et al. Respiratory viruses, a common microbiological finding in children treated for cancer in South Africa. Pediatric Blood and Cancer. 2015;62(Supplement 4):S386-S7.
60. Nieminen R, Koistinen A, Vuorinen T, Arku B, Soderlund-Venermo M, Ruuskanen O, et al. Rhinovirus genotypes in the first wheeze. Allergy: European Journal of Allergy and Clinical Immunology. 2013;68(SUPPL. 97):626.
61. Passi A, Pagani C, Gramegna D, Daffini R, Pollara CP, Signorini L, et al. Respiratory viruses infections are a significant clinical problem in haematological patients with underestimated adverse outcome: A single institution 9-years experience. Haematologica. 2019;104(Supplement 2):29-30.
62. Perez GF, Pancham K, Huseni S, Jain A, Rodriguez-Martinez CE, Preciado D, et al. Cytokine airway responses to acute rhinovirus infection and respiratory morbidity in severe premature children. American Journal of Respiratory and Critical Care Medicine. 2015;191(MeetingAbstracts).
63. Quevedo Teruel SJ, Gonzalez Carrasco E, Calvo Rey C, Gomez Salazar JM, Beato Merino M, Garcia Garcia ML. Severe respiratory infections in children who were premature. Journal of Perinatal Medicine. 2015;43(SUPPL. 1).
64. Quinonez AD, Restrepo SM, Unigarro MF, Arjona JS, Zuluaga CA, Bohorquez AP. CLINICAL BEHAVIOR OF PEDIATRIC PATIENTS WITH RESPIRATORY INFECTION BY RHINOVIRUS COMPARED TO INFECTION BY OTHER VIRUSES*IN A HIGH COMPLEXITY INSTITUTION IN BOGOTA. Pediatric Critical Care Medicine. 2022;23(11 Supplement 1).
65. Rebaza AP, Bermejo SD, Sharma L, Gomez JL, Dela Cruz C. Characteristics and outcomes of pediatric patients with recurrent respiratory viral infection. American Journal of Respiratory and Critical Care Medicine. 2020;201(1).
66. Reese OD, Tippett A, Hussaini L, Salazar L, Taylor M, Ciric C, et al. The Burden of Influenza and Rhinovirus among Hospitalized Adults Post the COVID-19 Pandemic. Open Forum Infectious Diseases. 2021;8(SUPPL 1):S757-S8.
67. Restrepo SM, Quinonez-Lopez A, Unigarro-Martinez M, Arjona-Caycedo J, Zuluaga C, Bohorquez-Penaranda A. Respiratory Infection by Rhinovirus Compared to Infection by Other Viruses, in a High Complexity Institution in Bogota, Colombia. American Journal of Respiratory and Critical Care Medicine. 2023;207(1).
68. Roussy JF, Carbonneau J, Hamelin ME, Boivin G. Distribution of rhinoviruses in hospitalized and nonhospitalized children with respiratory tract infections. Canadian Journal of Infectious Diseases and Medical Microbiology. 2013;24(SUPPL. SB):18B-9B.
69. Russell CJ, Kono N, Rush M, Hahn A, Morrison JM, Cogen JD, et al. Bacterial-viral Co-infection and Outcomes in Pediatric Bacterial Tracheostomy-associated Infections: A Multicenter Prospective Cohort Study. American Journal of Respiratory and Critical Care Medicine. 2023;207(1).
70. Russell CJ, Neely MN, Ward SL, Newth CJ, Simon TD. Viral co-infection is associated with poorer outcomes in pediatric bacterial tracheostomy-associated infections. American Journal of Respiratory and Critical Care Medicine. 2020;201(1).
71. Ruthrich MM, Schmitt T, Classen AY, Von Bergwelt-Baildon M, Khodamoradi Y, Doleschall AD, et al. Oncology Research and Treatment. 2022;45(Supplement 2):249.
72. Sarria EE, Mattiello R, Silva ER, Arruda Neto E, Pinto LA, Pitrez PC, et al. Increased severity of LRTI in Brazilian young children with co-detection of RSV and HRV. American Journal of Respiratory and Critical Care Medicine. 2012;185(MeetingAbstracts).
73. Schuster J, Banerjee D, Hassan F, Boom J, Englund J, Halasa N, et al. Rhinovirus detection in children hospitalized with acute respiratory illness and asymptomatic outpatients. Open Forum Infectious Diseases. 2017;4(Supplement 1):S570.
74. Seo S, Martin E, Xie H, Kuypers JM, Campbell AP, Choi SM, et al. Human rhinovirus rna detection in the lower respiratory tract of hematopoietic cell transplant recipients: Association with mortality. Biology of Blood and Marrow Transplantation. 2013;19(2 SUPPL. 1):S167-S8.
75. Setter NW, Peres ML, De Almeida BMM, Raboni SM. Charlson comorbidity index scores and in-hospital prognosis in severe acute respiratory infections patients. Open Forum Infectious Diseases. 2019;6(Supplement 2):S753.
76. Shaar A, Rebeca Da Silva Sena C, Morten M, Meredith J, Kepreotes E, Murphy V, et al. Rhinovirus bronchiolitis during infancy and later pre-school multiple breath washout. Respirology. 2021;26(SUPPL 2):77.
77. Shim JY, Park HG, Kim S, Lee M. Trends of 5-year ambient air pollutant levels and hospitalization rate due to respiratory viral infection in Korean children. Respirology. 2023;28(Supplement 1):105.
78. Singh SP, Kovacs C. CLINICAL IMPACT OF COMMUNITY ACQUIRED RESPIRATORY VIRUSES IN PATIENTS WITH SOLID ORGAN TRANSPLANTS. Chest. 2022;162(4 Supplement):A488.
79. Song Y, Tang MF, Tsun JGS, Pun JCS, Wong GWK, Chan RWY, et al. Interactive effects between rhinovirus subtypes and CDHR3 genotypes for severity of respiratory tract infections. Archives of Disease in Childhood. 2021;106(SUPPL 1):A237.
80. Spaeder M, Custer J, Miles A, Razzi L, Morin N, Scafidi S, et al. A multi-center outcomes analysis of children with severe respiratory infection caused by rhinovirus. Critical Care Medicine. 2013;41(12 SUPPL. 1):A135.
81. Srinivasan J, Mc IC, Soo S, Diggle M, Vyas H. The molecular epidemiology and clinical disease severity of human Rhino virus infections in hospitalised children. Archives of Disease in Childhood. 2016;101(Supplement 1):A309-A10.
82. Sudarwati SS. Etiology and outcome of lower respiratory tract infection in children. Pediatric Pulmonology. 2014;49(SUPPL. 37):S83-S4.
83. Suzuki A, Lupisan S, Fuji N, Ohno A, Furuse Y, Tamaki R, et al. Etiology of childhood pneumonia in Tacloban, the Philippines. International Journal of Infectious Diseases. 2010;14(SUPPL. 1):e27.
84. Suzuki F, Katsurada N, Aoshima M, Yamawaki S, Otsuki A, Watanabe J, et al. Viral infection in patients with nursing and healthcare-associated pneumonia (NHCAP). Respirology. 2014;19(SUPPL. 3):221.
85. Taplitz R, Maziarz RT, Mulroney C, Perales M, Romee R, Goldsmith SR, et al. Incidence and Impact of Community Respiratory Viral Infection (CRV) in Haploidentical and Matched Sibling Donors Receiving Post-Transplant Cyclophosphamide (PTCy): A CIBMTR Analysis. Biology of Blood and Marrow Transplantation. 2020;26(3 Supplement):S71-S2.
86. Taremi M, Shah D, El-Haddad D, El Chaer F, El Haddad L, Prayag A, et al. Rhinovirus infections (RhVI) in 233 hematopoietic cell transplant (HCT) recipients: A single center experience. Open Forum Infectious Diseases. 2016;3(Supplement 1).
87. Taremi M, Shah DP, El Haddad D, El Chaer F, El Haddad L, Chemaly RF. Clinical impact of rhinovirus infection early after hematopoietic stem cell transplantation (HCT). Bone Marrow Transplantation. 2016;51(SUPPL. 1):S394-S5.
88. Trikha G, Conway R, Hiemenz JW, Ljungman PT. Epidemiology and outcomes of respiratory virus infections in adult acute leukemia and hematopoietic stem cell transplant patients. Blood. 2014;124(21).
89. Trikha G, Hiemenz J, Wingard JR, Zou F, LeBlanc K, Mattsson J, et al. Community acquired respiratory viral infections (CARV) in patients with acute leukemia and hematopoietic stem cell transplant (HSCT) recipients. Biology of Blood and Marrow Transplantation. 2016;22(3 SUPPL. 1):S178.
90. Tripathi S, Gladfelter T, Al-Sayyed B. Disease course and outcomes of children hospitalized with single versus multiple virus co-infections. Critical Care Medicine. 2021;49(1 SUPPL 1):491.
91. Tugcu D, Valiyeva L, Sahin S, Tuna R, Bilici M, Unuvar A, et al. EVALUATION OF VIRAL RESPIRATORY TRACT INFECTIONS IN PEDIATRIC HEMATOLOGY-ONCOLOGY PATIENTS BEFORE COVID-19 PANDEMY. Hematology, Transfusion and Cell Therapy. 2023;45(Supplement 3):S29.
92. Versluys AB, Rossen JWA, Van Ewijk B, Schuurman R, Bierings M, Boelens JJ. Common cold viruses leading to life-threatening alloimmune lung syndromes. Bone Marrow Transplantation. 2010;45(SUPPL. 2):S122.
93. Waddey K, Barney J. SOLID ORGAN TRANSPLANT PATIENTS HAVE HIGHER RATES OF NON-CMV VIRAL PNEUMONIA COMPARED TO IMMUNOCOMPETENT HOSTS. Chest. 2020;157(6 Supplement):A451.
94. Waghmare A, Xie H, Kuypers JM, Sorror ML, Leisenring WM, Englund JA, et al. Human rhinovirus infections in hematopoietic cell transplant recipients: Factors determining progression to lower tract disease. Biology of Blood and Marrow Transplantation. 2017;23(3 Supplement 1):S29.
95. Weinstock JJ, Arroyo Morr MA, Salka K, Chorvinsky E, Xu Chen X, Perez GF, et al. Viral respiratory infections in severely premature infants: Is it appropriate to call it viral bronchiolitis? American Journal of Respiratory and Critical Care Medicine. 2020;201(1).
96. Weresk R, Lo TYM, Harvala H, Templeton K. Clinical relevance of rhinovirus infections in children at the critical care unit. Journal of Clinical Virology. 2015;70(SUPPL. 1):S52-S3.
97. Williams LW. The presence of rhinovirus in lower airways of patients with bronchial asthma. Pediatrics. 2008;122(SUPPL. 4):S206.
98. Yoshida LM, Suzuki M, Le MN, Le HT, Morimoto K, Moriuchi H, et al. Respiratory syncytial virus multiple viral infection and risk of pediatric pneumonia in central vietnam. American Journal of Tropical Medicine and Hygiene. 2012;87(5 SUPPL. 1):73.
99. Yun KW, Wallihan R, Desai AP, Alter SJ, Ambroggio L, Cohen DM, et al. Clinical characteristics and etiology of community-acquired pneumonia in children: A contemporary, prospective, multicenter study in Ohio, 2015-2018. Open Forum Infectious Diseases. 2019;6(Supplement 2):S911-S2.
100. Zinna S, Soo S, Sharkey D. The impact of respiratory viruses on neonatal intensive care patients: The importance of rhinovirus. Archives of Disease in Childhood: Fetal and Neonatal Edition. 2014;99(SUPPL. 1):A45-A6.
101. Brandwein A, Esperanza M, Rosen L, Schneider J. Incidence of respiratory failure in children with rhinovirus infection. American Journal of Respiratory and Critical Care Medicine. 2014;189(MeetingAbstracts).

**2. No eligible outcome (n = 97)**

1. Abu Elhassan UE, Mohamed SAA, Rizk MS, Sherif M, El-Harras M. Outcomes of patients with Severe Acute Respiratory Infections (SARI) admitted to the intensive care unit: results from the Egyptian Surveillance Study 2010-2014. Multidisciplinary respiratory medicine. 2020;15(1):465.
2. Agarwal A, Chakma N, Manchanda V, Dabas A. Virological profile of upper respiratory tract infections in children under 5 years of age- a cross sectional study in a tertiary care hospital in North India. Indian Journal of Medical Microbiology. 2023;44((Agarwal, Chakma, Dabas) Department of Paediatrics, Maulana Azad Medical College, New Delhi, India(Manchanda) Department of Microbiology, Maulana Azad Medical College, New Delhi, India):100378.
3. Al-Turab M, Chehadeh W, Al-Mulla F, Al-Nakib W. Human metapneumovirus in patients with respiratory tract infection in Kuwait. Journal of medical virology. 2011;83(10):1811-7.
4. Alzahrani RS, Alzahrani M, Shuraim W, Aldibasi O, Albarrak K, Habib A, et al. Outcome of Respiratory Viral Infections in Hematopoietic Stem Cell Transplant Recipients. Transplantation Proceedings. 2024;56(1):186-90.
5. Anders KL, Nguyen HL, Nguyen NM, Van Thuy NT, Hong Van NT, Hieu NT, et al. Epidemiology and virology of acute respiratory infections during the first year of life: a birth cohort study in Vietnam. The Pediatric infectious disease journal. 2015;34(4):361-70.
6. Aponte FE, Taboada B, Espinoza MA, Arias-Ortiz MA, Monge-Martinez J, Rodriguez-Vazquez R, et al. Rhinovirus is an important pathogen in upper and lower respiratory tract infections in Mexican children. Virology journal. 2015;12(101231645):31.
7. Avcu G, Bal ZS, Cicek C, Vardar F. Clinical and epidemiological evaluation of hospitalized children with respiratory virus infections. Cocuk Enfeksiyon Dergisi. 2017;11(3):e95-e9.
8. Baillie VL, Moore DP, Mathunjwa A, Morailane P, Simoes EAF, Madhi SA. Molecular Subtyping of Human Rhinovirus in Children from Three Sub-Saharan African Countries. Journal of clinical microbiology. 2019;57(9).
9. Banjar H, Chaballout M, Karkour K, Al-Ghamdi H, Al-Mogarri I, Al-Haider S, et al. The prevalence of viral infections in children with cystic fibrosis in a tertiary care center in Saudi Arabia. International Journal of Pediatrics and Adolescent Medicine. 2020;7(2):83-7.
10. Barral S, Mamin A, Dantin C, Masouridi-Levrat S, Chalandon Y, Kaiser L, et al. Rhinovirus Infections among Hematopoietic Stem Cell Transplant Recipients: A Pre-Transplant Dilemma? Viruses. 2022;14(2).
11. Benites ECA, Cabrini DP, Silva ACB, Silva JC, Catalan DT, Berezin EN, et al. Acute respiratory viral infections in pediatric cancer patients undergoing chemotherapy. Jornal de pediatria. 2014;90(4):370-6.
12. Boon H, Meinders AJ, van Hannen EJ, Tersmette M, Schaftenaar E. Comparative analysis of mortality in patients admitted with an infection with influenza A/B virus, respiratory syncytial virus, rhinovirus, metapneumovirus or SARS-CoV-2. Influenza and other Respiratory Viruses. 2024;18(1):e13237.
13. Brestovac B, Lawrence C, Speers DJ, Sammels LM, Mulrennan S. Respiratory viral infections in Western Australians with cystic fibrosis. Respiratory medicine. 2020;161(8908438, rme):105854.
14. Calvo C, Casas I, Garcia-Garcia ML, Pozo F, Reyes N, Cruz N, et al. Role of rhinovirus C respiratory infections in sick and healthy children in Spain. The Pediatric infectious disease journal. 2010;29(8):717-20.
15. Caserta MT, Yang H, Bandyopadhyay S, Qiu X, Gill SR, Java J, et al. Measuring the Severity of Respiratory Illness in the First 2 Years of Life in Preterm and Term Infants. Journal of Pediatrics. 2019;214((Caserta, Scheible, Pryhuber) Department of Pediatrics, University of Rochester Medical Center, Rochester, NY, United States(Yang, Bandyopadhyay, Qiu, McDavid, Holden-Wiltse) Department of Biostatistics and Computational Biology, University of Rochester M):12-9.e3.
16. Chidekel AS, Rosen CL, Bazzy AR. Rhinovirus infection associated with serious lower respiratory illness in patients with bronchopulmonary dysplasia. The Pediatric infectious disease journal. 1997;16(1):43-7.
17. Chiu SS, Ho PL, Peiris MJS, Chan KH, Chan ELY. Population-based hospitalization incidence of respiratory viruses in community-acquired pneumonia in children younger than 5 years of age. Influenza and other Respiratory Viruses. 2014;8(6):626-7.
18. Choi EH, Lee HJ, Kim SJ, Eun BW, Kim NH, Lee JA, et al. The association of newly identified respiratory viruses with lower respiratory tract infections in Korean children, 2000-2005. Clinical Infectious Diseases. 2006;43(5):585-92.
19. Chorazy ML, Lebeck MG, McCarthy TA, Richter SS, Torner JC, Gray GC. Polymicrobial acute respiratory infections in a hospital-based pediatric population. The Pediatric infectious disease journal. 2013;32(5):460-6.
20. Cilla G, Onate E, Perez-Yarza EG, Montes M, Vicente D, Perez-Trallero E. Viruses in community-acquired pneumonia in children aged less than 3 years old: High rate of viral coinfection. Journal of medical virology. 2008;80(10):1843-9.
21. Colombo RE, Schofield C, Richard SA, Fairchok M, Chen WJ, Danaher PJ, et al. Effects of human immunodeficiency virus status on symptom severity in influenza-like illness in an otherwise healthy adult outpatient cohort. Journal of Investigative Medicine. 2021;69(6):1230-7.
22. Delgado-Corcoran C, Witte MK, Ampofo K, Castillo R, Bodily S, Bratton SL. The impact of human rhinovirus infection in pediatric patients undergoing heart surgery. Pediatric cardiology. 2014;35(8):1387-94.
23. Do LAH, Bryant JE, Tran AT, Nguyen BH, Tran TTL, Tran QH, et al. Respiratory Syncytial Virus and Other Viral Infections among Children under Two Years Old in Southern Vietnam 2009-2010: Clinical Characteristics and Disease Severity. PloS one. 2016;11(8):e0160606.
24. Drysdale SB, Alcazar M, Wilson T, Smith M, Zuckerman M, Hodemaekers HM, et al. Functional and genetic predisposition to rhinovirus lower respiratory tract infections in prematurely born infants. European journal of pediatrics. 2016;175(12):1943-9.
25. El-Sahly HM, Atmar RL, Glezen WP, Greenberg SB. Spectrum of clinical illness in hospitalized patients with "common cold" virus infections. Clinical infectious diseases : an official publication of the Infectious Diseases Society of America. 2000;31(1):96-100.
26. Erdem SB, Can D, Girit S, Catal F, Sen V, Pekcan S, et al. Does atopy affect the course of viral pneumonia? Allergologia et immunopathologia. 2018;46(2):119-26.
27. Etemadi MR, Othman N, Savolainen-Kopra C, Sekawi Z, Wahab N, Sann LM. Biodiversity and clinico-demographic characteristics of human rhinoviruses from hospitalized children with acute lower respiratory tract infections in Malaysia. Journal of clinical virology : the official publication of the Pan American Society for Clinical Virology. 2013;58(4):671-7.
28. Fujiogi M, Camargo CA, Jr., Raita Y, Bochkov YA, Gern JE, Mansbach JM, et al. Respiratory viruses are associated with serum metabolome among infants hospitalized for bronchiolitis: A multicenter study. Pediatric allergy and immunology : official publication of the European Society of Pediatric Allergy and Immunology. 2020;31(7):755-66.
29. Ghani ASA, Morrow BM, Hardie DR, Argent AC. An investigation into the prevalence and outcome of patients admitted to a pediatric intensive care unit with viral respiratory tract infections in Cape Town, South Africa. Pediatric critical care medicine : a journal of the Society of Critical Care Medicine and the World Federation of Pediatric Intensive and Critical Care Societies. 2012;13(5):e275-81.
30. Giamberardin HIG, Homsani S, Bricks LF, Pacheco APO, Guedes M, Debur MC, et al. Clinical and epidemiological features of respiratory virus infections in preschool children over two consecutive influenza seasons in southern Brazil. Journal of medical virology. 2016;88(8):1325-33.
31. Gooskens J, van der Ploeg V, Sukhai RN, Vossen ACTM, Claas ECJ, Kroes ACM. Clinical evaluation of viral acute respiratory tract infections in children presenting to the emergency department of a tertiary referral hospital in the Netherlands. BMC Pediatrics. 2014;14(1):297.
32. Greenberg SB. Viral respiratory infections in elderly patients and patients with chronic obstructive pulmonary disease. The American journal of medicine. 2002;112 Suppl 6A(0267200, 3ju):28S-32S.
33. Hedberg P, Karlsson Valik J, Van Der Werff S, Tanushi H, Requena Mendez A, Granath F, et al. Clinical phenotypes and outcomes of SARS-CoV-2, influenza, RSV and seven other respiratory viruses: A retrospective study using complete hospital data. Thorax. 2022;77(2):1-10.
34. Hellferscee O, Treurnicht FK, Walaza S, Du Plessis M, Von Gottberg A, Wolter N, et al. The Fraction of Rhinovirus Detections Attributable to Mild and Severe Respiratory Illness in a Setting of High Human Immunodeficiency Virus Prevalence, South Africa, 2013-2015. The Journal of infectious diseases. 2019;219(11):1697-704.
35. Horvat C, Casalegno JS, Masson E, Benveniste C, Haesebaert J, Paget J, et al. Contribution of Infant Rhinovirus Bronchiolitis to Hospital Bed and Ventilation Use. JAMA Network Open. 2024;7(2):E2355033.
36. Iroh Tam P-Y, Zhang L, Cohen Z. Clinical characteristics and outcomes of human rhinovirus positivity in hospitalized children. Annals of thoracic medicine. 2018;13(4):230-6.
37. Jiang W, Wu M, Zhou J, Wang Y, Hao C, Ji W, et al. Etiologic spectrum and occurrence of coinfections in children hospitalized with community-acquired pneumonia. BMC infectious diseases. 2017;17(1):787.
38. Kandeel A, Fahim M, Deghedy O, W HR, M KK, El Shesheny R, et al. Multicenter study to describe viral etiologies, clinical profiles, and outcomes of hospitalized children with severe acute respiratory infections, Egypt 2022. Scientific reports. 2023;13(1):21860.
39. Kantor DB, Stenquist N, McDonald MC, Schultz BJ, Hauptman M, Smallwood CD, et al. Rhinovirus and serum IgE are associated with acute asthma exacerbation severity in children. The Journal of allergy and clinical immunology. 2016;138(5):1467-71.e9.
40. Khan T, Das RS, Chaudhary A, Chatterjee J, Bhattacharya SD. Association of nasopharyngeal viruses and pathogenic bacteria in children and their parents with and without HIV. Pneumonia (Nathan Qld). 2021;13(1):8.
41. Kim DR, Kim KR, Park H, Park E, Cho J, Kim J, et al. Severe Human Rhinovirus Lower Respiratory Tract Infections in Young Children. Pediatric Infection and Vaccine. 2023;30(3):111-20.
42. Kim YJ, Waghmare A, Xie H, Holmberg L, Pergam SA, Jerome KR, et al. Respiratory viruses in hematopoietic cell transplant candidates: impact of preexisting lower tract disease on outcomes. Blood Advances. 2022;6(18):5307-16.
43. Kloepfer KM, Sarsani VK, Poroyko V, Lee WM, Pappas TE, Kang T, et al. Community-acquired rhinovirus infection is associated with changes in the airway microbiome. The Journal of allergy and clinical immunology. 2017;140(1):312-5.e8.
44. Korsun N, Angelova S, Trifonova I, Voleva S, Grigorova I, Tzotcheva I, et al. Predominance of ON1 and BA9 genotypes of respiratory syncytial virus (RSV) in Bulgaria, 2016-2018. Journal of Medical Virology. 2021;93(6):3401-11.
45. Kouni S, Karakitsos P, Chranioti A, Theodoridou M, Chrousos G, Michos A. Evaluation of viral co-infections in hospitalized and non-hospitalized children with respiratory infections using microarrays. Clinical microbiology and infection : the official publication of the European Society of Clinical Microbiology and Infectious Diseases. 2013;19(8):772-7.
46. Kraft CS, Jacob JT, Sears MH, Burd EM, Caliendo AM, Lyon GM. Severity of human rhinovirus infection in immunocompromised adults is similar to that of 2009 H1N1 influenza. Journal of clinical microbiology. 2012;50(3):1061-3.
47. Lambert KA, Prendergast LA, Dharmage SC, Tang M, O'Sullivan M, Tran T, et al. The role of human rhinovirus (HRV) species on asthma exacerbation severity in children and adolescents. The Journal of asthma : official journal of the Association for the Care of Asthma. 2018;55(6):596-602.
48. Lauinger IL, Bible JM, Halligan EP, Bangalore H, Tosas O, Aarons EJ, et al. Patient characteristics and severity of human rhinovirus infections in children. Journal of clinical virology : the official publication of the Pan American Society for Clinical Virology. 2013;58(1):216-20.
49. Laurent C, Dugue AE, Brouard J, Nimal D, Dina J, Parienti JJ, et al. Viral epidemiology and severity of respiratory infections in infants in 2009: A prospective study. Pediatric Infectious Disease Journal. 2012;31(8):827-31.
50. Leotte J, Trombetta H, Faggion HZ, Almeida BM, Nogueira MB, Vidal LR, et al. Impact and seasonality of human rhinovirus infection in hospitalized patients for two consecutive years. Jornal de pediatria. 2017;93(3):294-300.
51. Li L, Wang C, Sun L, Zhang X, Yang G. Clinical characteristics and prognostic risk factors of mortality in patients with interstitial lung diseases and viral infection: a retrospective cohort study. Journal of medical microbiology. 2021;70(11).
52. Louie JK, Yagi S, Nelson FA, Kiang D, Glaser CA, Rosenberg J, et al. Rhinovirus outbreak in a long term care facility for elderly persons associated with unusually high mortality. Clinical infectious diseases : an official publication of the Infectious Diseases Society of America. 2005;41(2):262-5.
53. Luchsinger V, Ampuero S, Palomino MA, Chnaiderman J, Levican J, Gaggero A, et al. Comparison of virological profiles of respiratory syncytial virus and rhinovirus in acute lower tract respiratory infections in very young Chilean infants, according to their clinical outcome. Journal of clinical virology : the official publication of the Pan American Society for Clinical Virology. 2014;61(1):138-44.
54. Maffey AF, Barrero PR, Venialgo C, Fernandez F, Fuse VA, Saia M, et al. Viruses and atypical bacteria associated with asthma exacerbations in hospitalized children. Pediatric pulmonology. 2010;45(6):619-25.
55. Mak RKY, Tse LY, Lam WY, Wong GWK, Chan PKS, Leung TF. Clinical spectrum of human rhinovirus infections in hospitalized Hong Kong children. The Pediatric infectious disease journal. 2011;30(9):749-53.
56. Marcone DN, Ellis A, Videla C, Ekstrom J, Ricarte C, Carhallal G, et al. Viral etiology of acute respiratory infections in hospitalized and outpatient children in Buenos Aires, Argentina. Pediatric Infectious Disease Journal. 2013;32(3):e105-e10.
57. McManus TE, Marley A-M, Baxter N, Christie SN, O'Neill HJ, Elborn JS, et al. Respiratory viral infection in exacerbations of COPD. Respiratory medicine. 2008;102(11):1575-80.
58. Miller EK, Bugna J, Libster R, Shepherd BE, Scalzo PM, Acosta PL, et al. Human rhinoviruses in severe respiratory disease in very low birth weight infants. Pediatrics. 2012;129(1):e60-7.
59. Miller EK, Edwards KM, Weinberg GA, Iwane MK, Griffin MR, Hall CB, et al. A novel group of rhinoviruses is associated with asthma hospitalizations. The Journal of allergy and clinical immunology. 2009;123(1):98-104.e1.
60. Miller EK, Linder J, Kraft D, Johnson M, Lu P, Saville BR, et al. Hospitalizations and outpatient visits for rhinovirus-associated acute respiratory illness in adults. The Journal of allergy and clinical immunology. 2016;137(3):734-43.e1.
61. Miller EK, Lu X, Erdman DD, Poehling KA, Zhu Y, Griffin MR, et al. Rhinovirus-associated hospitalizations in young children. The Journal of infectious diseases. 2007;195(6):773-81.
62. Miller EK, Williams JV, Gebretsadik T, Carroll KN, Dupont WD, Mohamed YA, et al. Host and viral factors associated with severity of human rhinovirus-associated infant respiratory tract illness. The Journal of allergy and clinical immunology. 2011;127(4):883-91.
63. Monteiro AIMP, Bellei NCJ, Sousa AR, dos Santos AMN, Weckx LY. Respiratory infections in children up to two years of age on prophylaxis with palivizumab. Revista Paulista de Pediatria. 2014;32(2):152-8.
64. Nascimento MS, Souza AVd, Ferreira AVdS, Rodrigues JC, Abramovici S, Silva Filho LVFd. High rate of viral identification and coinfections in infants with acute bronchiolitis. Clinics (Sao Paulo, Brazil). 2010;65(11):1133-7.
65. Neugebauer F, Bergs S, Liebert UG, Honemann M. Human Rhinoviruses in Pediatric Patients in a Tertiary Care Hospital in Germany: Molecular Epidemiology and Clinical Significance. Viruses. 2022;14(8).
66. Nicolai A, Frassanito A, Nenna R, Cangiano G, Petrarca L, Papoff P, et al. Risk Factors for Virus-induced Acute Respiratory Tract Infections in Children Younger Than 3 Years and Recurrent Wheezing at 36 Months Follow-Up After Discharge. The Pediatric infectious disease journal. 2017;36(2):179-83.
67. O'Callaghan C, Diez N, Bassat Q, Morais L, MacHevo S, Nhamposa T, et al. Epidemiology of viral pneumonia among young children admitted to hospital in rural Mozambique. Tropical Medicine and International Health. 2009;14(SUPPL. 2):214-5.
68. O'Callaghan-Gordo C, Bassat Q, Morais L, Diez-Padrisa N, Machevo S, Nhampossa T, et al. Etiology and epidemiology of viral pneumonia among hospitalized children in rural Mozambique: a malaria endemic area with high prevalence of human immunodeficiency virus. The Pediatric infectious disease journal. 2011;30(1):39-44.
69. O'Callaghan-Gordo C, Diez-Padrisa N, Abacassamo F, Perez-Brena P, Casas I, Alonso PL, et al. Viral acute respiratory infections among infants visited in a rural hospital of southern Mozambique. Tropical medicine & international health : TM & IH. 2011;16(9):1054-60.
70. Olabarrieta I, Gonzalez-Carrasco E, Calvo C, Pozo F, Casas I, Garcia-Garcia ML. Hospital admission due to respiratory viral infections in moderate preterm, late preterm and term infants during their first year of life. Allergologia et immunopathologia. 2015;43(5):469-73.
71. Pitrez PMC, Stein RT, Stuermer L, Macedo IS, Schmitt VM, Jones MH, et al. [Rhinovirus and acute bronchiolitis in young infants]. Jornal de pediatria. 2005;81(5):417-20.
72. Prill MM, Dahl RM, Midgley CM, Chern S-WW, Lu X, Feikin DR, et al. Severe Respiratory Illness Associated With Rhinovirus During the Enterovirus D68 Outbreak in the United States, August 2014-November 2014. Clinical infectious diseases : an official publication of the Infectious Diseases Society of America. 2018;66(10):1528-34.
73. Ramgopal S, Cotter JM, Navanandan N, Shah SS, Ruddy RM, Ambroggio L, et al. Viral Detection Is Associated With Severe Disease in Children With Suspected Community-Acquired Pneumonia. Pediatric Emergency Care. 2023;39(7):465-9.
74. Renois F, Leveque N, Deliege P-G, Fichel C, Bouin A, Abely M, et al. Enteroviruses as major cause of microbiologically unexplained acute respiratory tract infections in hospitalized pediatric patients. The Journal of infection. 2013;66(6):494-502.
75. Rhoden J, Hoffmann AT, Stein JF, Rocha BSD, Barros VMD, Silva EVD, et al. Viral coinfection in hospitalized patients during the COVID-19 pandemic in Southern Brazil: a retrospective cohort study. Respiratory Research. 2024;25(1):71.
76. Rudi JM, Molina F, Diaz R, Bonet V, Ortellao L, Cantarutti D, et al. The role of rhinovirus in children hospitalized for acute respiratory disease, Santa Fe, Argentina. Journal of medical virology. 2015;87(12):2027-32.
77. Sanchez Garcia L, Calvo C, Casas I, Pozo F, Pellicer A. Viral respiratory infections in very low birthweight infants at neonatal intensive care unit: prospective observational study. BMJ paediatrics open. 2020;4(1):e000661.
78. Self WH, Williams DJ, Zhu Y, Ampofo K, Pavia AT, Chappell JD, et al. Respiratory viral detection in children and adults: Comparing asymptomatic controls and patients with community-acquired pneumonia. Journal of Infectious Diseases. 2016;213(4):584-91.
79. Seo YB, Song JY, Choi MJ, Kim IS, Yang TU, Hong KW, et al. Etiology and clinical outcomes of acute respiratory virus infection in hospitalized adults. Infection and Chemotherapy. 2014;46(2):67-76.
80. Shih H-I, Wang H-C, Su I-J, Hsu H-C, Wang J-R, Sun HFS, et al. Viral Respiratory Tract Infections in Adult Patients Attending Outpatient and Emergency Departments, Taiwan, 2012-2013: A PCR/Electrospray Ionization Mass Spectrometry Study. Medicine. 2015;94(38):e1545.
81. Shorr AF, Ilges DT, Micek ST, Kollef MH. The importance of viruses in ventilator-associated pneumonia. Infection control and hospital epidemiology. 2023;44(7):1137-42.
82. Sik N, Cakan Baserdem KA, Baserdem O, Appak O, Sayiner AA, Yilmaz D, et al. Distribution of Viral Respiratory Pathogens During the COVID-19 Pandemic: A Single-Center Pediatric Study from Turkey. Turkish Archives of Pediatrics. 2022;57(3):354-9.
83. Smit PM, Bongers KM, Kuiper RJL, von Rosenstiel IA, Smits PHM, Brandjes DPM. Characterization of 2009 H1N1 pandemic influenza in a population of Dutch children with influenza-like signs and symptoms. Acta paediatrica (Oslo, Norway : 1992). 2012;101(1):67-72.
84. Sun H, Sun J, Ji W, Hao C, Yan Y, Chen Z, et al. Impact of RSV Coinfection on Human Bocavirus in Children with Acute Respiratory Infections. Journal of tropical pediatrics. 2019;65(4):342-51.
85. Takia L, Anguranq SK, Angurana SK, Sarkar S, Bora I, Ratho RK, et al. Clinico-virological profile, intensive care needs, and outcome of infants with acute viral bronchiolitis: A prospective observational study. Indian Journal of Critical Care Medicine. 2021;25(SUPPL 1):S8-S9.
86. Turunen R, Jartti T, Bochkov YA, Gern JE, Vuorinen T. Rhinovirus species and clinical characteristics in the first wheezing episode in children. Journal of medical virology. 2016;88(12):2059-68.
87. Vanmali H, Everson F, de Beer C. Detection of human rhinovirus in Sudden Unexpected Death in Infancy (SUDI) cases at Tygerberg medico-legal mortuary, Cape Town, South Africa. African Journal of Respiratory Medicine. 2022;17(7).
88. Williams AL, Uren EC, Bretherton L. Respiratory viruses and sudden infant death. British Medical Journal. 1984;288(6429):1491-3.
89. Xiang Z, Gonzalez R, Xie Z, Xiao Y, Chen L, Li Y, et al. Human rhinovirus group C infection in children with lower respiratory tract infection. Emerging infectious diseases. 2008;14(10):1665-7.
90. Yamaguto GE, Zhen F, Moreira MM, Montesanti BM, Raboni SM. Community respiratory viruses and healthcare-associated infections: epidemiological and clinical aspects. Journal of Hospital Infection. 2022;122((Yamaguto, Zhen, Moreira, Raboni) Infectious Diseases Division, Complexo Hospital de Clinicas, Universidade Federal Do Parana, Curitiba, Brazil(Montesanti) Hospital Epidemiology Division, Complexo Hospital de Clinicas, Universidade Federal Do Parana, Curi):187-93.
91. Yan Y, Huang L, Wang M, Wang Y, Ji W, Zhu C, et al. Clinical and epidemiological profiles including meteorological factors of low respiratory tract infection due to human rhinovirus in hospitalized children. Ital J Pediatr. 2017;43(1):23.
92. Yan Y, Sun J, Ji K, Guo J, Han L, Li F, et al. High incidence of the virus among respiratory pathogens in children with lower respiratory tract infection in northwestern China. Journal of medical virology. 2023;95(1):e28367.
93. Yen C-Y, Wu W-T, Chang C-Y, Wong Y-C, Lai C-C, Chan Y-J, et al. Viral etiologies of acute respiratory tract infections among hospitalized children - A comparison between single and multiple viral infections. Journal of microbiology, immunology, and infection = Wei mian yu gan ran za zhi. 2019;52(6):902-10.
94. Sayama A, Okamoto M, Tamaki R, Saito-Obata M, Saito M, Kamigaki T, et al. Comparison of Rhinovirus A-, B-, and C-Associated Respiratory Tract Illness Severity Based on the 5'-Untranslated Region Among Children Younger Than 5 Years. Open forum infectious diseases. 2022;9(10):ofac387.
95. I G Giamberardino H, O Pacheco AP, Pereira LA, Debur MdC, Genehold G, Raboni SM. Respiratory syncytial virus: host genetic susceptibility and factors associated with disease severity in a cohort of pediatric patients. Journal of tropical pediatrics. 2022;68(6).
96. Marcone DN, Culasso A, Carballal G, Campos R, Echavarria M. Genetic diversity and clinical impact of human rhinoviruses in hospitalized and outpatient children with acute respiratory infection, Argentina. Journal of clinical virology : the official publication of the Pan American Society for Clinical Virology. 2014;61(4):558-64.
97. Lucena CM, Torres A, Rovira M, Marcos MA, de la Bellacasa JP, Sanchez M, et al. Pulmonary complications in hematopoietic SCT: a prospective study. Bone marrow transplantation. 2014;49(10):1293-9.

**3. No risk factor or comparator (n = 42)**

1. Aramburo A, van Schaik S, Louie J, Boston E, Messenger S, Wright C, et al. Role of real-time reverse transcription polymerase chain reaction for detection of respiratory viruses in critically ill children with respiratory disease: Is it time for a change in algorithm? Pediatric critical care medicine : a journal of the Society of Critical Care Medicine and the World Federation of Pediatric Intensive and Critical Care Societies. 2011;12(4):e160-5.
2. Baillie VL, Moore DP, Mathunjwa A, Baggett HC, Brooks A, Feikin DR, et al. Epidemiology of the Rhinovirus (RV) in African and Southeast Asian Children: A Case-Control Pneumonia Etiology Study. Viruses. 2021;13(7).
3. Bouzid D, Hadad O, Bertine M, Houhou-Fidouh N, Mirand A, Duval X, et al. Rhinoviruses: molecular diversity and clinical characteristics. International journal of infectious diseases : IJID : official publication of the International Society for Infectious Diseases. 2022;118(c3r, 9610933):144-9.
4. Calvo C, Garcia-Garcia ML, Pozo F, Paula G, Molinero M, Calderon A, et al. Respiratory Syncytial Virus Coinfections With Rhinovirus and Human Bocavirus in Hospitalized Children. Medicine. 2015;94(42):e1788.
5. Costa LF, Queiroz DAO, Lopes da Silveira H, Bernardino Neto M, de Paula NT, Oliveira TFMS, et al. Human rhinovirus and disease severity in children. Pediatrics. 2014;133(2):e312-21.
6. Cox DW, Khoo S-K, Zhang G, Lindsay K, Keil AD, Knight G, et al. Rhinovirus is the most common virus and rhinovirus-C is the most common species in paediatric intensive care respiratory admissions. The European respiratory journal. 2018;52(2).
7. De Luca M, D'Amore C, Romani L, Tripiciano C, Clemente V, Mercadante S, et al. Severe viral respiratory infections in the pre-COVID era: A 5-year experience in two pediatric intensive care units in Italy. Influenza and other respiratory viruses. 2023;17(1):e13038.
8. De Rose DU, Maddaloni C, Martini L, Ronci S, Pugnaloni F, Marrocco G, et al. Are lung ultrasound features more severe in infants with bronchiolitis and coinfections? Frontiers in Pediatrics. 2023;11((De Rose, Maddaloni, Martini, Ronci, Pugnaloni, Ronchetti, Calzolari, Dotta) Neonatal Intensive Care Unit, "Bambino Gesu" Children's Hospital IRCCS, Rome, Italy(De Rose) PhD Course in Microbiology, Immunology, Infectious Diseases, and Transplants (MIMIT)):1238522.
9. Dogan A, Ersoy Cinar Y, Otlu B, Kuzucu C. Investigation of Viral and Atypical Pathogens in Patients with Pneumonia Who Need Intensive Care Unit. Flora. 2022;27(1):28-36.
10. Ekinci Sert S, Karagol C, Gungor A, Gulhan B. Comparison of Clinical, Demographic Features, and Costs in Respiratory Syncytial Virus, Rhinovirus, and Viral Co-infections in Children Hospitalized with Viral Infections of the Lower Respiratory Tract. Japanese journal of infectious diseases. 2022;75(2):164-8.
11. Esposito S, Daleno C, Tagliabue C, Scala A, Tenconi R, Borzani I, et al. Impact of rhinoviruses on pediatric community-acquired pneumonia. European journal of clinical microbiology & infectious diseases : official publication of the European Society of Clinical Microbiology. 2012;31(7):1637-45.
12. Fujiogi M, Camargo CA, Jr., Raita Y, Bochkov YA, Gern JE, Mansbach JM, et al. Association of rhinovirus species with nasopharyngeal metabolome in bronchiolitis infants: A multicenter study. Allergy. 2020;75(9):2379-83.
13. Furuse Y, Tamaki R, Suzuki A, Kamigaki T, Okamoto M, Saito-Obata M, et al. Epidemiological and clinical characteristics of children with acute respiratory viral infections in Philippines: a prospective cohort study. Clinical microbiology and infection : the official publication of the European Society of Clinical Microbiology and Infectious Diseases. 2020((Furuse) Institute for Frontier Life and Medical Sciences, Kyoto University, Kyoto, Japan; Hakubi Center for Advanced Research, Kyoto University, Kyoto, Japan(Tamaki, Suzuki, Kamigaki, Okamoto, Saito, Oshitani) Department of Virology, Tohoku University Gr).
14. George RB, Mogabgab WJ. Atypical pneumonia in young men with rhinovirus infections. Annals of internal medicine. 1969;71(6):1073-8.
15. Golke P, Honemann M, Bergs S, Liebert UG. Human Rhinoviruses in Adult Patients in a Tertiary Care Hospital in Germany: Molecular Epidemiology and Clinical Significance. Viruses. 2021;13(10).
16. Gulla KM, Balaji A, Mukherjee A, Jat KR, Sankar J, Lodha R, et al. Course of illness after viral infection in Indian children with cystic fibrosis. Journal of Tropical Pediatrics. 2019;65(2):176-82.
17. Hasegawa K, Pate BM, Mansbach JM, Macias CG, Fisher ES, Piedra PA, et al. Risk factors for requiring intensive care among children admitted to ward with bronchiolitis. Academic pediatrics. 2015;15(1):77-81.
18. Hassan IA, Chopra R, Swindell R, Mutton KJ. Respiratory viral infections after bone marrow/peripheral stem-cell transplantation: the Christie hospital experience. Bone marrow transplantation. 2003;32(1):73-7.
19. Hong KW, Choi SM, Lee DG, Cho SY, Lee HJ, Choi JK, et al. Lower Respiratory Tract Diseases Caused by Common Respiratory Viruses among Stem Cell Transplantation Recipients: A Single Center Experience in Korea. Yonsei medical journal. 2017;58(2):362-9.
20. Johnston SL, Pattemore PK, Sanderson G, Smith S, Campbell MJ, Josephs LK, et al. The relationship between upper respiratory infections and hospital admissions for asthma: A time-trend analysis. American Journal of Respiratory and Critical Care Medicine. 1996;154(3 I):654-60.
21. Kapur N, Mackay IM, Sloots TP, Masters IB, Chang AB. Respiratory viruses in exacerbations of non-cystic fibrosis bronchiectasis in children. Archives of disease in childhood. 2014;99(8):749-53.
22. Karppinen S, Terasjarvi J, Auranen K, Schuez-Havupalo L, Siira L, He Q, et al. Acquisition and Transmission of Streptococcus pneumoniae Are Facilitated during Rhinovirus Infection in Families with Children. American journal of respiratory and critical care medicine. 2017;196(9):1172-80.
23. Li L, Hsu SH, Wang C, Li B, Sun L, Shi J, et al. Characteristics of viral pneumonia in non-HIV immunocompromised and immunocompetent patients: a retrospective cohort study. BMC Infectious Diseases. 2021;21(1):767.
24. Liang J, Wang Y, Liu Y, Li Q, Zeng Z, Yang Z, et al. Epidemiology dynamic of the common respiratory virus in winter-spring, 2018-2023 in Guangdong province, China. Journal of Thoracic Disease. 2023;15(12):7165-7.
25. Louie JK, Roy-Burman A, Guardia-Labar L, Boston EJ, Kiang D, Padilla T, et al. Rhinovirus associated with severe lower respiratory tract infections in children. The Pediatric infectious disease journal. 2009;28(4):337-9.
26. Mansbach JM, McAdam AJ, Clark S, Hain PD, Flood RG, Acholonu U, et al. Prospective multicenter study of the viral etiology of bronchiolitis in the emergency department. Academic emergency medicine : official journal of the Society for Academic Emergency Medicine. 2008;15(2):111-8.
27. Marinelli T, Wee LYA, Rowe E, Chhetri R, Friel O, Higgins G, et al. Respiratory Viruses Cause Late Morbidity in Recipients of Hematopoietic Stem Cell Transplantation. Biology of blood and marrow transplantation : journal of the American Society for Blood and Marrow Transplantation. 2020;26(4):782-8.
28. Martino R, Porras RP, Rabella N, Williams JV, Ramila E, Margall N, et al. Prospective study of the incidence, clinical features, and outcome of symptomatic upper and lower respiratory tract infections by respiratory viruses is adult recipients of hematopoietic stem cell transplants for hematologic malignancies. Biology of Blood and Marrow Transplantation. 2005;11(10):781-96.
29. Meier K, Riepl A, Voitl P, Lischka L, Voitl JJM, Langer K, et al. Characterisation of RSV infections in children without chronic diseases aged 0-36 months during the post-COVID-19 winter season 2022/2023. Frontiers in Pediatrics. 2024;12((Meier, Riepl, Voitl, Lischka, Voitl, Langer, Kuzio, Diesner-Treiber) First Vienna Pediatric Medical Center, Vienna, Austria(Voitl) Sigmund Freud University Vienna, Vienna, Austria(Redlberger-Fritz) Center of Virology, Medical University of Vienna, Vienna):1342399.
30. Paul SP, Mukherjee A, McAllister T, Harvey MJ, Clayton BA, Turner PC. Respiratory-syncytial-virus- and rhinovirus-related bronchiolitis in children aged <2 years in an English district general hospital. The Journal of hospital infection. 2017;96(4):360-5.
31. Richard N, Komurian-Pradel F, Javouhey E, Perret M, Rajoharison A, Bagnaud A, et al. The impact of dual viral infection in infants admitted to a pediatric intensive care unit associated with severe bronchiolitis. The Pediatric infectious disease journal. 2008;27(3):213-7.
32. Ruotsalainen M, Hyvarinen MK, Piippo-Savolainen E, Korppi M. Adolescent asthma after rhinovirus and respiratory syncytial virus bronchiolitis. Pediatric pulmonology. 2013;48(7):633-9.
33. Toivonen L, Camargo CA, Jr., Gern JE, Bochkov YA, Mansbach JM, Piedra PA, et al. Association between rhinovirus species and nasopharyngeal microbiota in infants with severe bronchiolitis. The Journal of allergy and clinical immunology. 2019;143(5):1925-8.e7.
34. Uzum O, Kanik A, Eliacik K, Hortu HO, Demircelik Y, Yan M, et al. Comparison of clinically related factors and treatment approaches in patients with acute bronchiolitis. Turkish Archives of Pediatrics. 2020;55(4):376-85.
35. Wald TG, Shult P, Krause P, Miller BA, Drinka P, Gravenstein S. A rhinovirus outbreak among residents of a long-term care facility. Annals of internal medicine. 1995;123(8):588-93.
36. Wan Q, Li YW, Cheng Y, Hu H. The Impact of Bacterial Co-Infection on Hospitalized Children with Human Rhinovirus and Human Metapneumovirus Infections: A Retrospective Analytical Cross-Sectional Study. Jundishapur Journal of Microbiology. 2023;16(9):e139106.
37. Wang K, Xi W, Yang D, Zheng Y, Zhang Y, Chen Y, et al. Rhinovirus is associated with severe adult community-acquired pneumonia in China. Journal of thoracic disease. 2017;9(11):4502-11.
38. Wang L, Allen J, Diong C, Goh Y-T, Gopalakrishnan S, Ho A, et al. Respiratory virus infection after allogeneic hematopoietic stem cell transplant in a tropical center: Predictive value of the immunodeficiency scoring index. Transplant infectious disease : an official journal of the Transplantation Society. 2017;19(3).
39. Willis AL, Calton JB, Calton J, Kim AS, Lee R, Torabzadeh E, et al. RV-C infections result in greater clinical symptoms and epithelial responses compared to RV-A infections in patients with CRS. Allergy. 2020;75(12):3264-7.
40. Wu D, Lu J, Sun Z, Cao L, Zeng Q, Liu Q, et al. Rhinovirus remains prevalent in school teenagers during fight against COVID-19 pandemic. Immunity, inflammation and disease. 2021;9(1):76-9.
41. Holster A, Terasjarvi J, Vuononvirta J, Koponen P, Peltola V, Helminen M, et al. Polymorphisms in the promoter region of IL10 gene are associated with virus etiology of infant bronchiolitis. World journal of pediatrics : WJP. 2018;14(6):594-600.
42. Jartti T, Hasegawa K, Mansbach JM, Piedra PA, Camargo CA, Jr. Rhinovirus-induced bronchiolitis: Lack of association between virus genomic load and short-term outcomes. The Journal of allergy and clinical immunology. 2015;136(2):509-12.e11.

**4. Wrong population or not laboratory-confirmed rhinovirus infection (n = 16)**

1. Abandeh FI, Lustberg M, Devine S, Elder P, Andritsos L, Martin SI. Outcomes of hematopoietic SCT recipients with rhinovirus infection: a matched, case-control study. Bone marrow transplantation. 2013;48(12):1554-7.
2. Baddal B, Bostanci A, Unal Evren E, Gazi U. Assessment of Respiratory Viral Co-infections Among SARS-CoV-2-Infected Patients. Flora. 2023;28(2):217-24.
3. Boonyaratanakornkit J, Vivek M, Xie H, Pergam SA, Cheng G-S, Mielcarek M, et al. Predictive Value of Respiratory Viral Detection in the Upper Respiratory Tract for Infection of the Lower Respiratory Tract With Hematopoietic Stem Cell Transplantation. The Journal of infectious diseases. 2020;221(3):379-88.
4. Choi YY, Kim YK, Choi EH. Clinical and epidemiological characteristics of common human coronaviruses in children: A single center study, 2015-2019. Pediatric Infection and Vaccine. 2021;28(2):101-9.
5. Chow EJ, Mermel LA. Hospital-acquired respiratory viral infections: Incidence, morbidity, and mortality in pediatric and adult patients. Open Forum Infectious Diseases. 2017;4(1).
6. Cohen-Hagai K, Rozenberg I, Korzets Ze, Zitman-Gal T, Einbinder Y, Benchetrit S. Upper Respiratory Tract Infection among Dialysis Patients. The Israel Medical Association journal : IMAJ. 2016;18(9):557-60.
7. Li W, Wang BH, Chen BH, Sun Y, Li L, Xiang WQ, et al. Coinfection of SARS-CoV-2 Omicron variant and other respiratory pathogens in children. World Journal of Pediatrics. 2024;20(1):92-6.
8. MacBean V, Lunt A, Drysdale SB, Yarzi MN, Rafferty GF, Greenough A. Predicting healthcare outcomes in prematurely born infants using cluster analysis. Pediatric pulmonology. 2018;53(8):1067-72.
9. Prasad N, Trenholme AA, Huang QS, Thompson MG, Pierse N, Widdowson MA, et al. Interactive effects of age and respiratory virus on severe lower respiratory infection. Epidemiology and Infection. 2018;146(14):1861-9.
10. Resch B, Puchas C, Resch E, Urlesberger B. Epidemiology of Respiratory Syncytial Virus-related Hospitalizations and the Influence of Viral Coinfections in Southern Austria in a 7-year Period. The Pediatric infectious disease journal. 2020;39(1):12-6.
11. Ryoo J, Kim SC, Lee J. Changes in respiratory infection trends during the COVID-19 pandemic in the haematologic malignancy patients. medRxiv. 2023((Ryoo) Division of Pulmonary and Critical Care Medicine, Department of Internal Medicine, Bucheon St. Mary's Hospital, College of Medicine, The Catholic University of Korea, Seoul, South Korea(Kim, Lee) Division of Pulmonary and Critical Care Medicine, De).
12. Shannon KL, Osula VO, Shaw-Saliba K, Hardick J, McBryde B, Dugas A, et al. Viral co-infections are associated with increased rates of hospitalization in those with influenza. Influenza and other respiratory viruses. 2022;16(4):780-8.
13. Yerkovich ST, Hales BJ, Carroll ML, Burel JG, Towers MA, Smith DJ, et al. Reduced rhinovirus-specific antibodies are associated with acute exacerbations of chronic obstructive pulmonary disease requiring hospitalisation. BMC pulmonary medicine. 2012;12(100968563):37.
14. Yesildag M, Bakdik BO, Senturk Z, Bekci TT. Clinical Characteristics of Hospitalized Patients with Viral Pneumonia Between October 2017 and April 2018. Eastern Journal of Medicine. 2024;29(1):29-38.
15. Moynihan KM, McGarvey T, Barlow A, Heney C, Gibbons K, Clark JE, et al. Testing for Common Respiratory Viruses in Children Admitted to Pediatric Intensive Care: Epidemiology and Outcomes. Pediatric critical care medicine : a journal of the Society of Critical Care Medicine and the World Federation of Pediatric Intensive and Critical Care Societies. 2020;21(6):e333-e41.
16. Gonzalez-Rosales N, Kasi AS, McCracken CE, Silva GL, Starks M, Stecenko A, et al. Impact of viral respiratory infections on pulmonary exacerbations in children with cystic fibrosis. Pediatric Pulmonology. 2023;58(3):871-7.

**5. Wrong study design (n = 15)**

1. Abbas S, Raybould JE, Sastry S, de la Cruz O. Respiratory viruses in transplant recipients: more than just a cold. Clinical syndromes and infection prevention principles. International journal of infectious diseases : IJID : official publication of the International Society for Infectious Diseases. 2017;62(c3r, 9610933):86-93.
2. Cavallazzi R, Ramirez JA. Influenza and Viral Pneumonia. Infectious Disease Clinics of North America. 2024;38(1):183-212.
3. Chetty A. Lower respiratory tract infection in children. Indian Pediatrics. 1985;22(9):637-40.
4. Chidekel AS, Bazzy AR, Rosen CL. Rhinovirus infection associated with severe lower respiratory tract illness and worsening lung disease in infants with bronchopulmonary dysplasia. Pediatric pulmonology. 1994;18(4):261-3.
5. Dong L, Xing L. Editorial: The biological mechanism and health effect of co-infection with multiple pathogens. Frontiers in Cellular and Infection Microbiology. 2024;14((Dong, Xing) Institutes of Biomedical Sciences, Shanxi University, Shanxi, Taiyuan, China(Dong, Xing) Shanxi Provincial Key Laboratory of Medical Molecular Cell Biology, Shanxi University, Taiyuan, China(Dong, Xing) Shanxi Provincial Key Laboratory for Pr):1370067.
6. El Idrissi KR, Isabel S, Carbonneau J, Lafond M, Quach C, Caya C, et al. Molecular and epidemiologic investigation of a rhinovirus outbreak in a neonatal intensive care unit. Infection control and hospital epidemiology. 2019;40(2):245-7.
7. Everman JL, Sajuthi S, Saef B, Rios C, Stoner AM, Numata M, et al. Functional genomics of CDHR3 confirms its role in HRV-C infection and childhood asthma exacerbations. Journal of Allergy and Clinical Immunology. 2019;144(4):962-71.
8. Falsey AR. Viral respiratory tract infections in elderly persons. Infectious Diseases in Clinical Practice. 1996;5(1):53-8.
9. Hohenthal U, Vainionpaa R, Nikoskelainen J, Kotilainen P. The role of rhinoviruses and enteroviruses in community acquired pneumonia in adults. Thorax. 2008;63(7):658-9.
10. Korppi M. The role of rhinoviruses is overestimated in the aetiology of community-acquired pneumonia in children. Acta paediatrica (Oslo, Norway : 1992). 2017;106(3):363-5.
11. Puro V, Minosse C, Cappiello G, Lauria FN, Capobianchi MR. Rhinovirus and lower respiratory tract infection in adults [5]. Clinical Infectious Diseases. 2005;40(7):1068-9.
12. Sandulescu M, Sandulescu O. Changing clinical patterns and ear-nose-throat complications of seasonal viral respiratory tract infections. GERMS. 2023;13(4):311-3.
13. Schmidt HJ, Fink RJ. Rhinovirus as a lower respiratory tract pathogen in infants. The Pediatric infectious disease journal. 1991;10(9):700-2.
14. Stott EJ. Rhinovirus infections in Glasgow in 1962-66. Bulletin of the World Health Organization. 1969;41(6):947-52.
15. Wick JY. Rhinovirus in the elderly: The commonest of colds. Pharmacy Times. 2015;81(1):1-2.

**6. Non-English publication (n = 11)**

1. Bichurina MA, Voloshchuk LV, Go A, Pisareva MM, Guzhov DA. Clinical features of rhinovirus infection in hospitalized adult patients during the epidemic season 2017-2018. Jurnal Infektologii. 2020;12(4):19-22.
2. Bueno Campana M, Calvo Rey C, Vazquez Alvarez MC, Parra Cuadrado E, Molina Amores C, Rodrigo Garcia G, et al. Viral respiratory tract infections in the first six months of life. Anales de Pediatria. 2008;69(5):400-5.
3. Hou M, Wang W, Zhai J, Fang Y, Wang L, Wu J, et al. Epidemiology and clinical characteristics of human rhinovirus infection in hospitalized children in Tianjin. Chinese Journal of Laboratory Medicine. 2021;44(4):317-22.
4. Kwon Y, Cho WJ, Kim HM, Lee J. Single or dual infection with respiratory syncytial virus and human rhinovirus: Epidemiology and clinical characteristics in hospitalized children in a rural area of South Korea. Pediatric Infection and Vaccine. 2019;26(2):99-111.
5. Lebecque P. Lower respiratory tract infections in children. Louvain Medical. 2002;121(5):S111-S20.
6. Li L, Ji W, Shao X-J, Xu J, Jiang W-J, Chen Z-R, et al. [An epidemiological study on human rhinovirus C in hospitalized children with respiratory tract infections]. Zhongguo dang dai er ke za zhi = Chinese journal of contemporary pediatrics. 2016;18(11):1094-9.
7. Matsumoto I, Yoshida S, Kawana R. [Virological surveillance of acute respiratory tract illnesses of children in Morioka, Japan. II. Rhinovirus infection]. Kansenshogaku zasshi The Journal of the Japanese Association for Infectious Diseases. 1991;65(10):1286-96.
8. Ruiz Contreras J, Rojo Conejo P. Community acquired pneumoniae in children. Revista Espanola de Pediatria. 2004;60(1):63-9.
9. Song M-H, Zhao L-Q, Qian Y, Zhu R-N, Deng J, Wang F, et al. [Human rhinovirus with different genotypes in children with acute respiratory tract infections in Beijing]. Bing du xue bao = Chinese journal of virology. 2013;29(2):97-105.
10. Vidaurreta SM, Marcone DN, Ellis A, Ekstrom J, Cukier D, Videla C, et al. Acute viral respiratory infection in children under 5 years. Epidemiological study in two centers in Buenos Aires, Argentina. Archivos Argentinos de Pediatria. 2011;109(4):296-304.
11. Wang H-h, Mao N-y, Xu S-t, Tang L-y, Wang H-l, Xie Z-d, et al. [The study of human rhinovirus in infants with lower respiratory tract infections]. Zhonghua shi yan he lin chuang bing du xue za zhi = Zhonghua shiyan he linchuang bingduxue zazhi = Chinese journal of experimental and clinical virology. 2011;25(2):120-2.

**7. Protocol (n = 1)**

1. Nct. A Study of Vapendavir Treatment of Hematopoietic Stem Cell Transplant Subjects With Symptomatic Rhinovirus Infection. 2017.

# Figure S1. Age <1 year as a risk factor for lower respiratory tract infection.


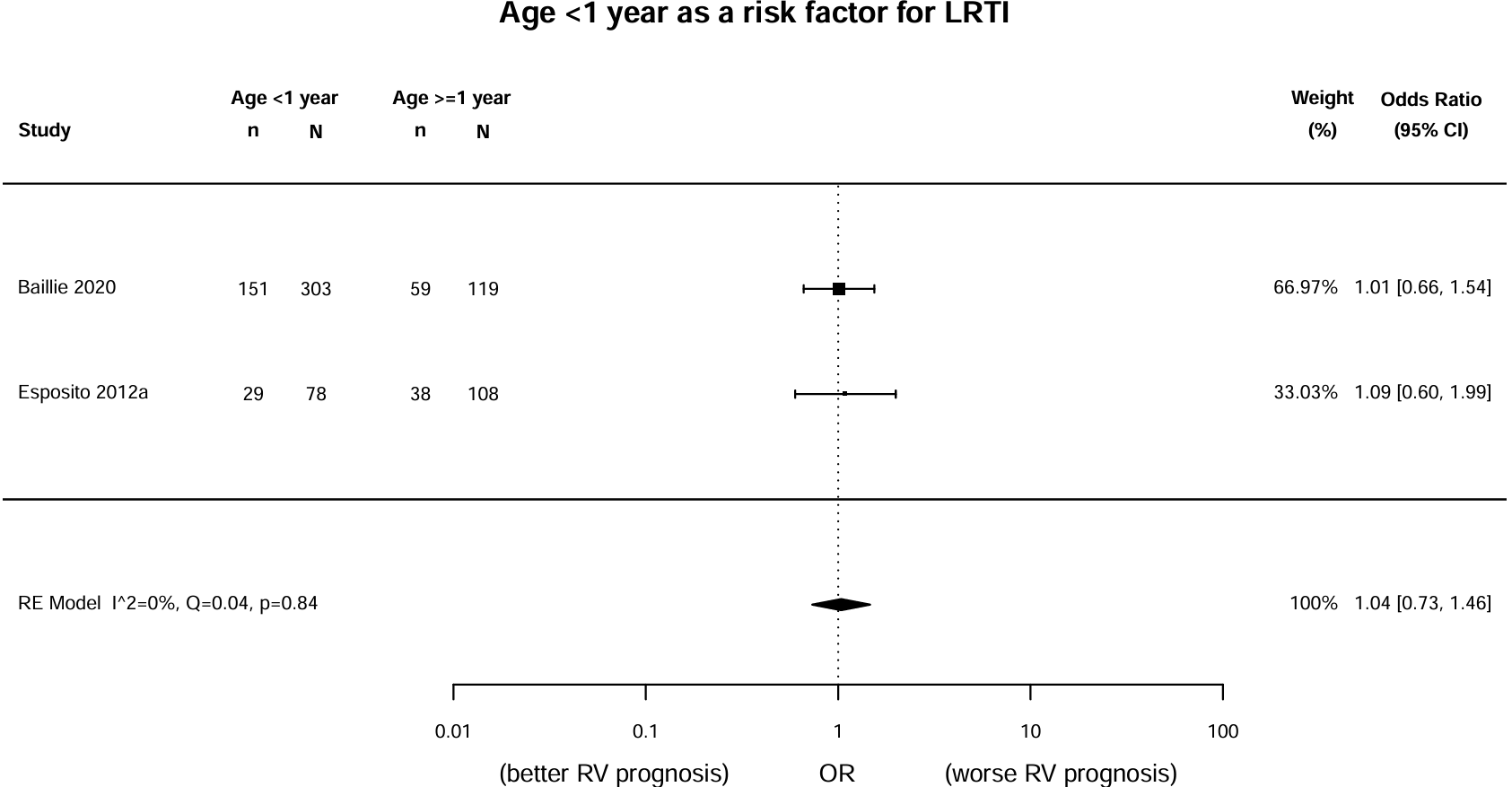


# Figure S2. Age ≥18 years as a risk factor for mortality.


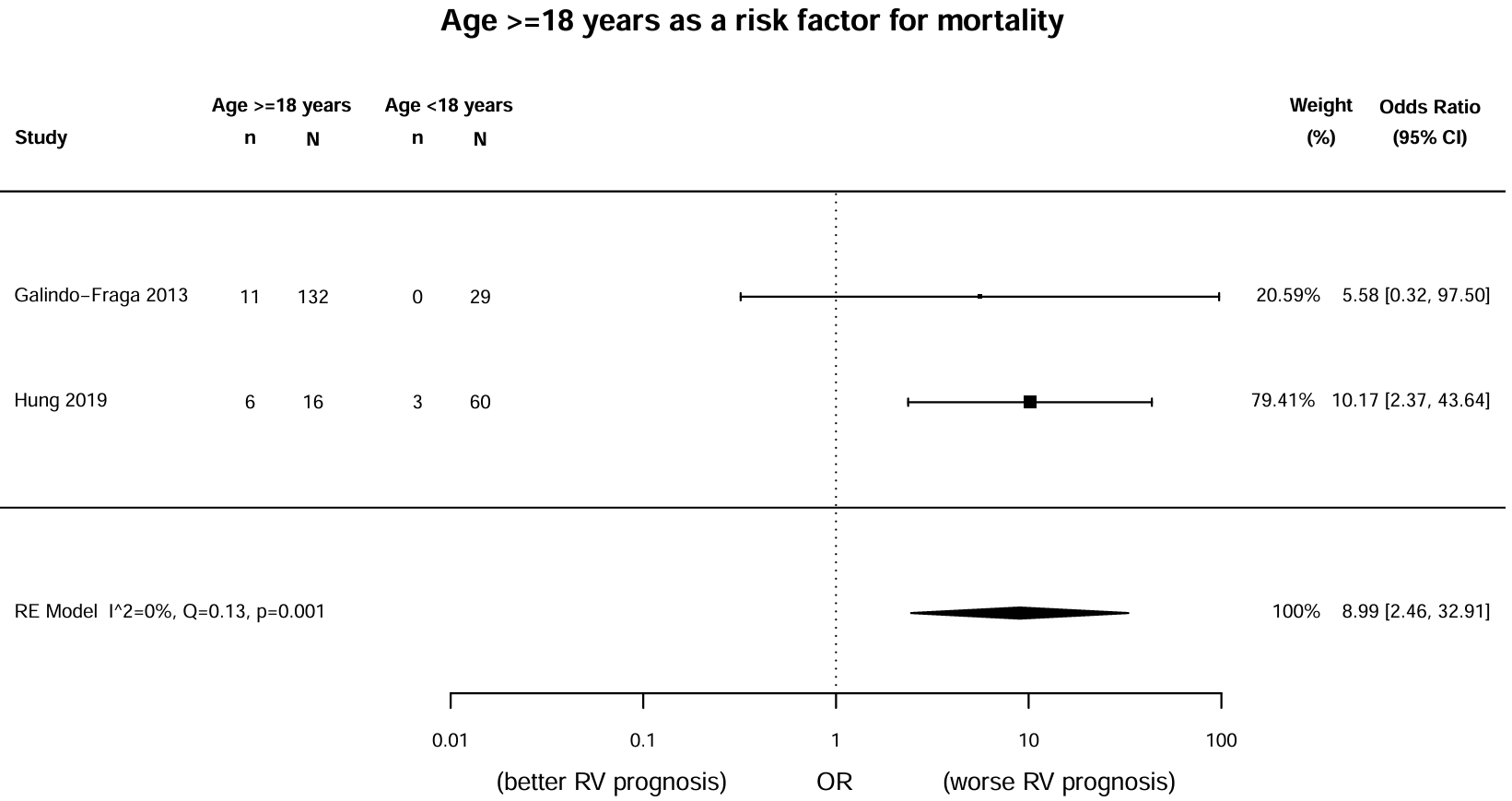


# Figure S3. Male sex as a risk factor for admission to a critical care unit.


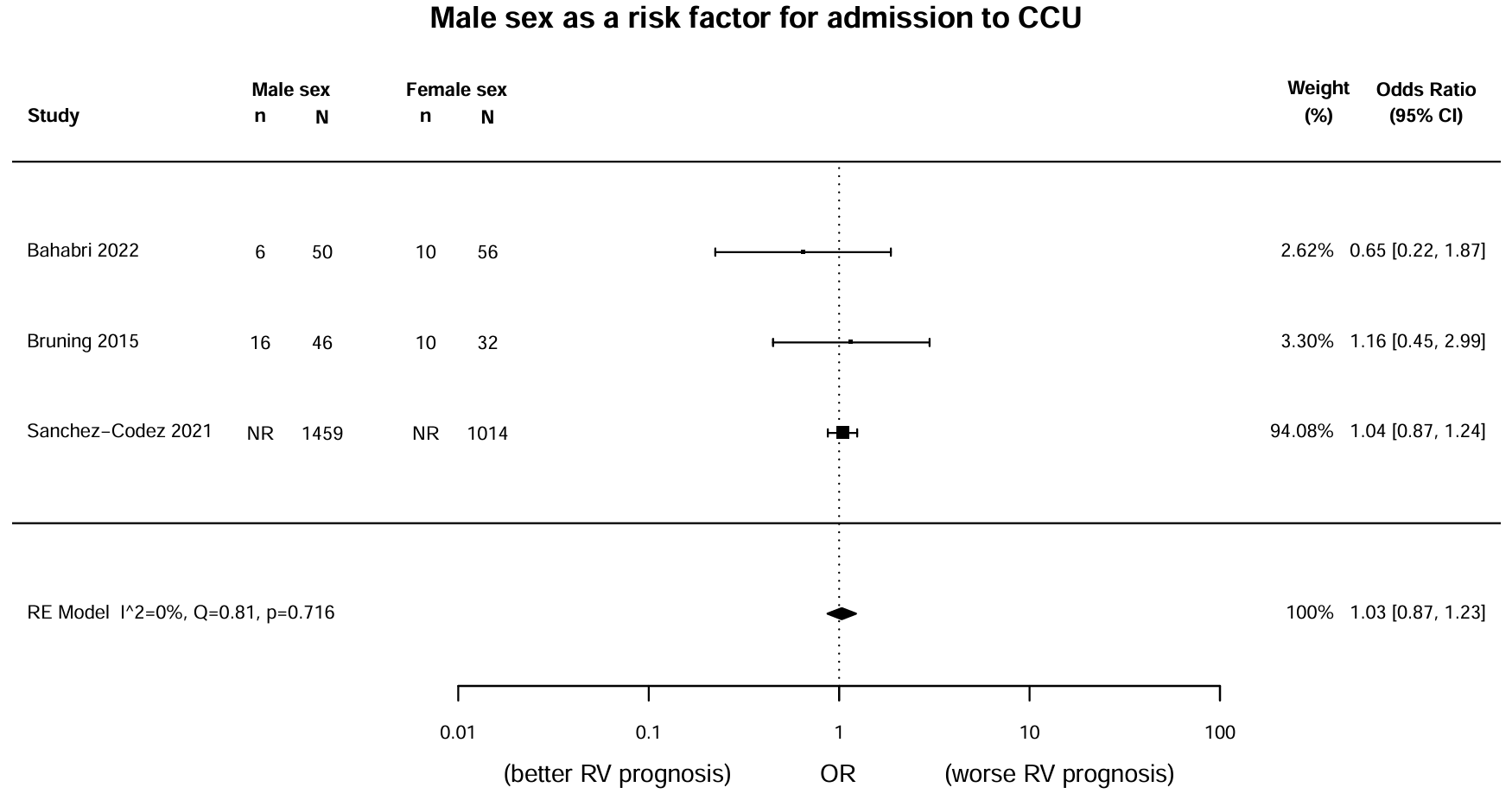


# Figure S4. Male sex as a risk factor for lower respiratory tract infection.


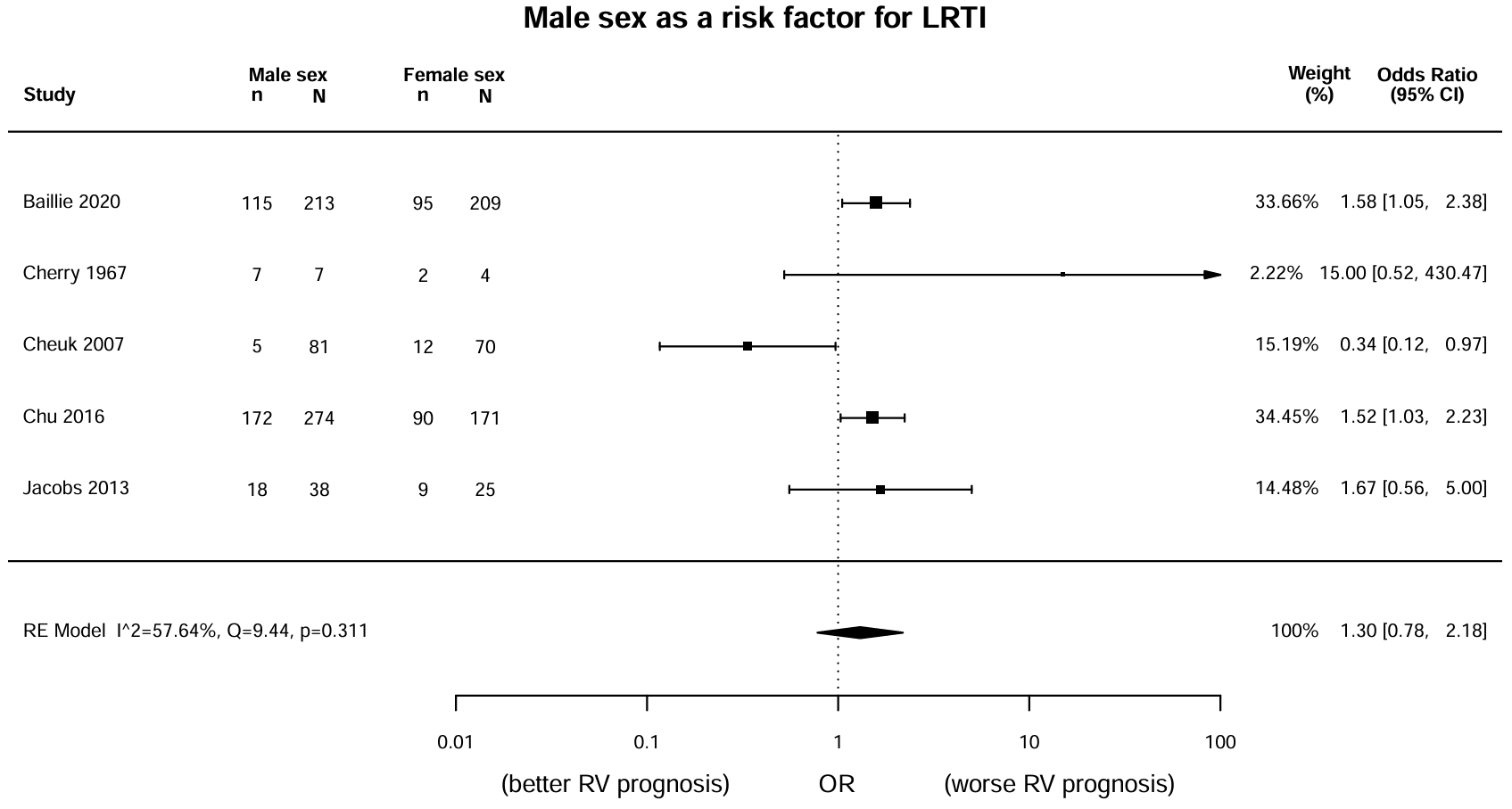


# Figure S5. Premature birth as a risk factor for lower respiratory tract infection.


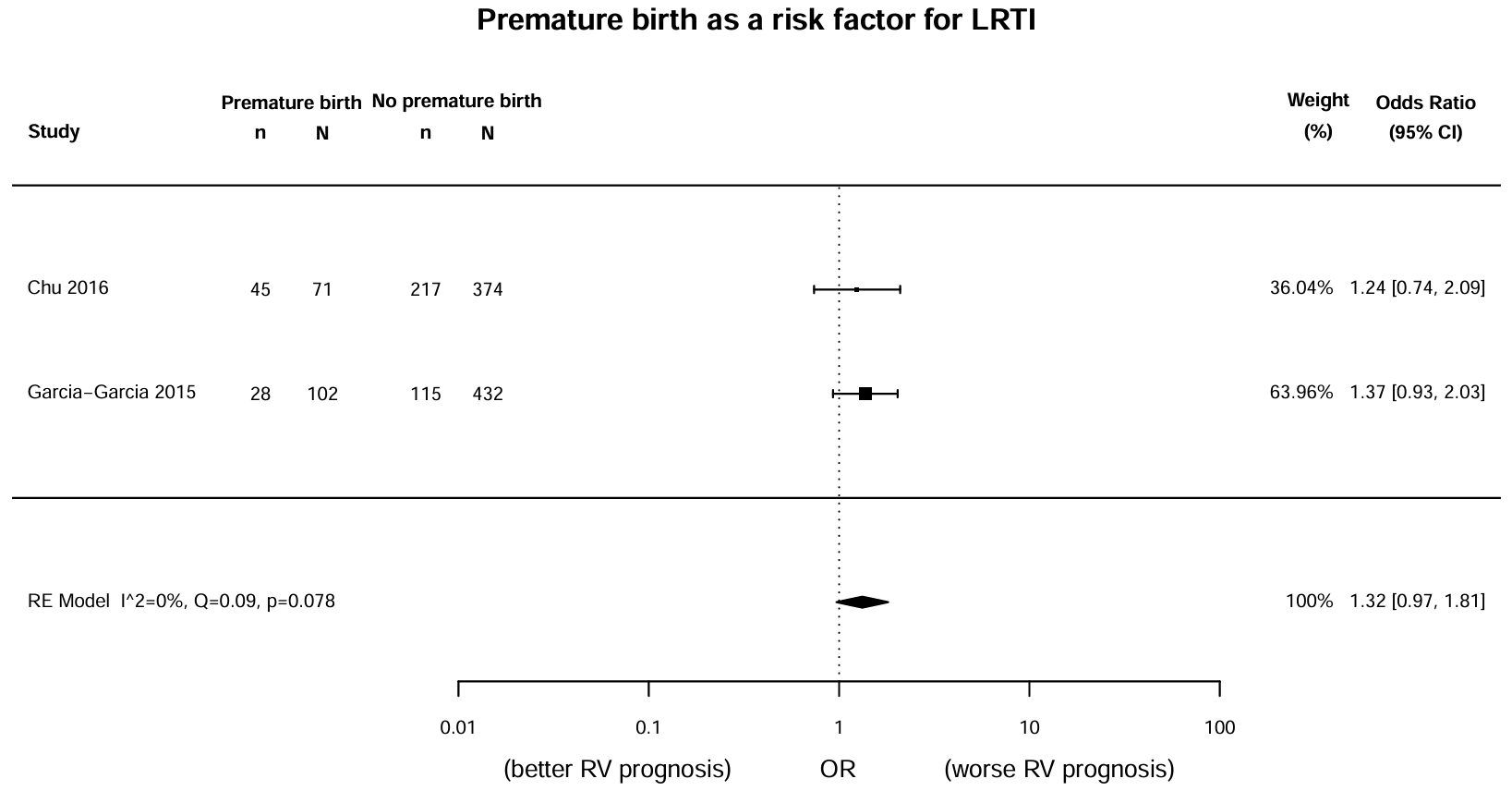


# Figure S6. Smoking as a risk factor for lower respiratory tract infection.


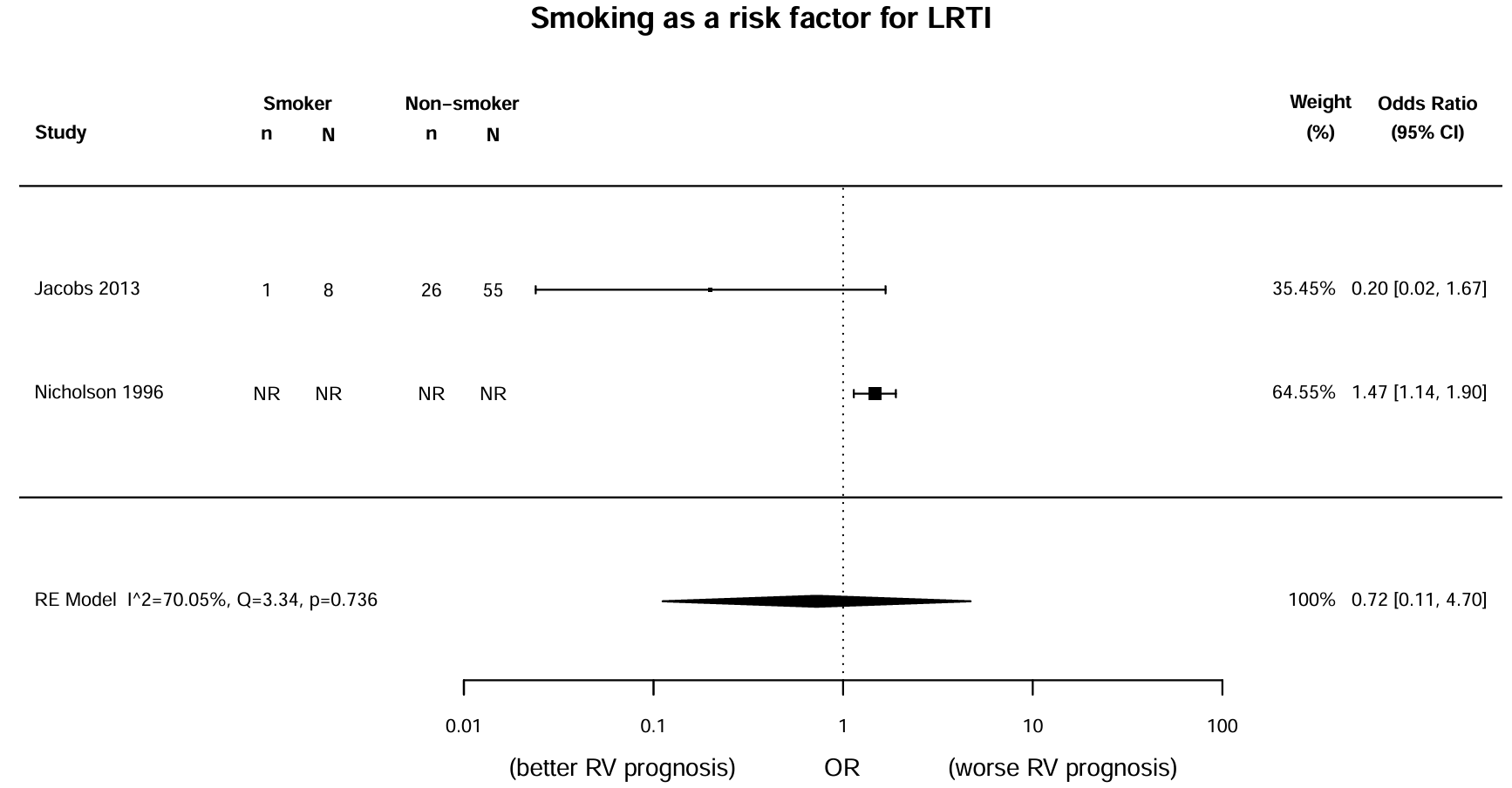


# Figure S7. Forest plot for any comorbidity as a risk factor for admission to a critical care unit.


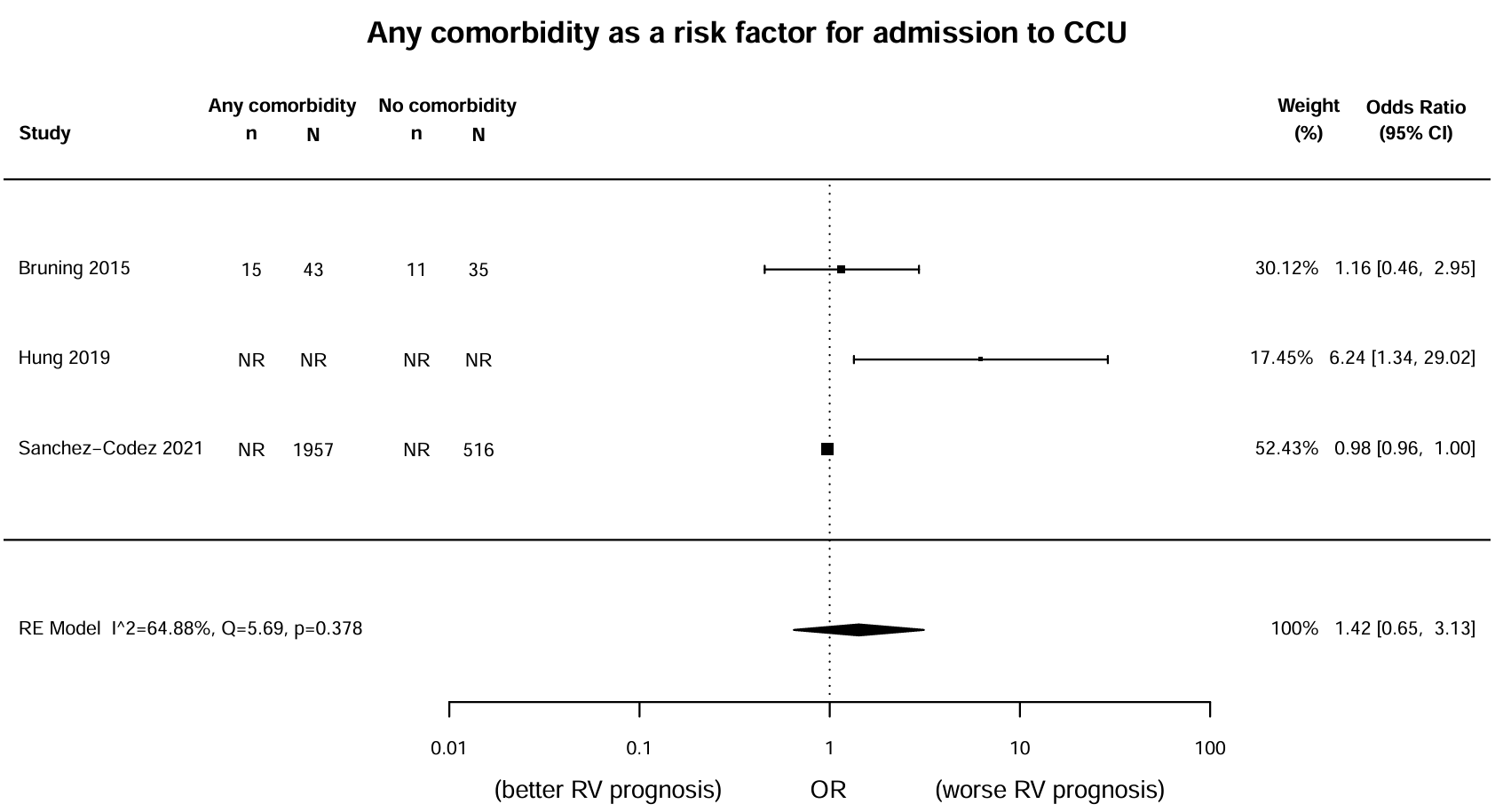


# Figure S8. Forest plot for any comorbidity as a risk factor for lower respiratory tract infection.


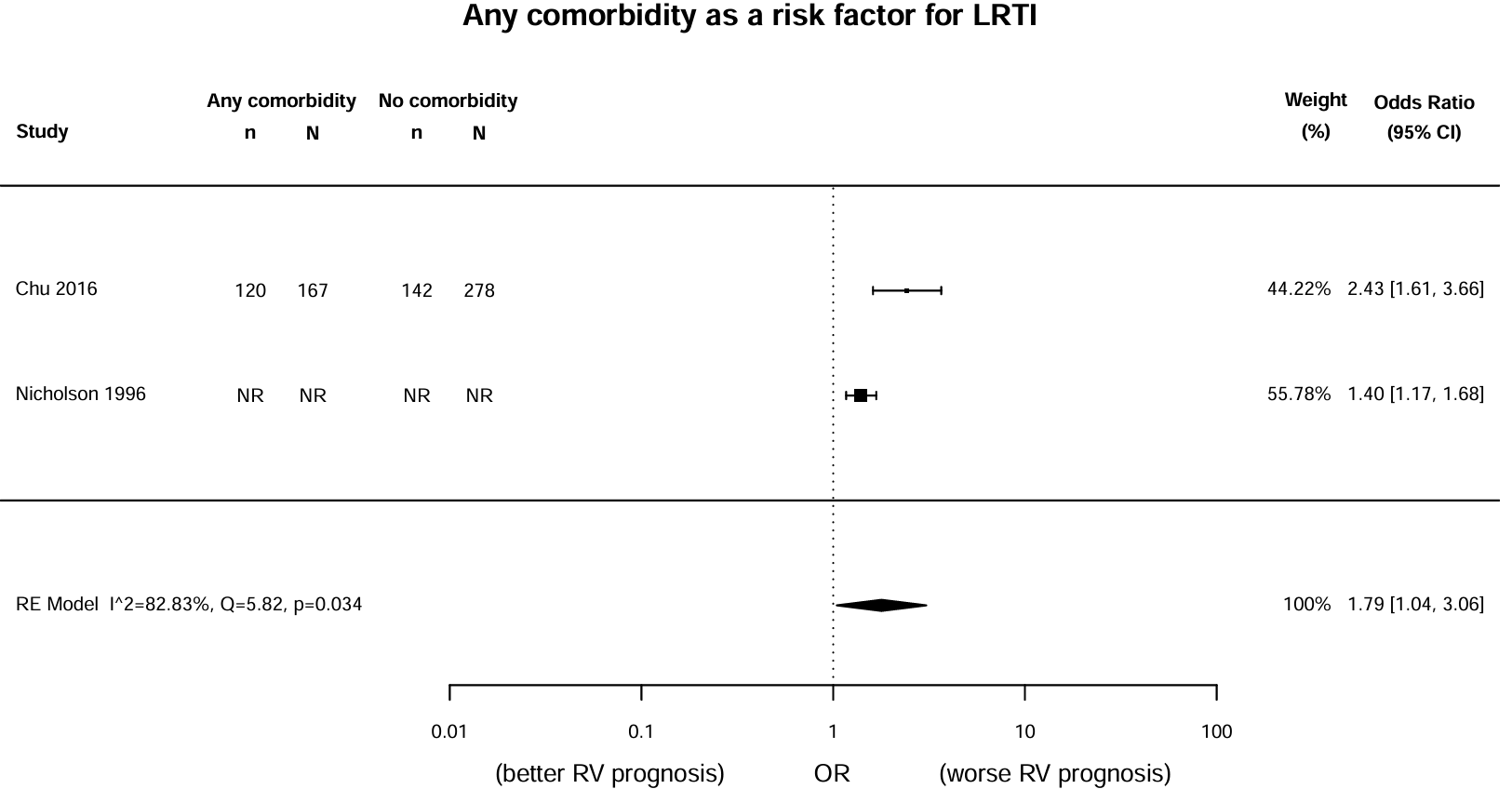


# Figure S9. Forest plot for diabetes mellitus as a risk factor for mortality.


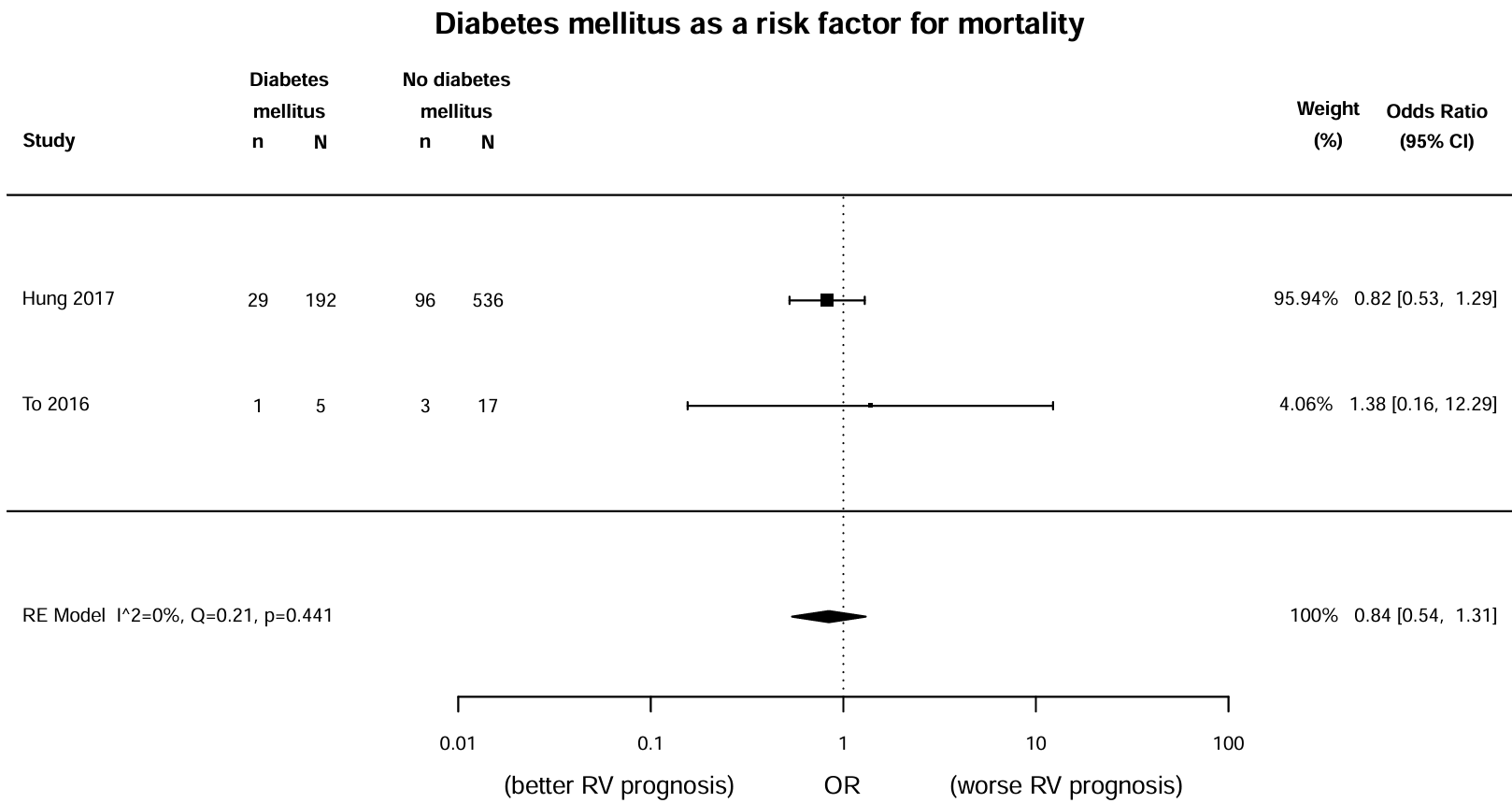


# Figure S10. Malignancy as a risk factor for admission to a critical care unit.


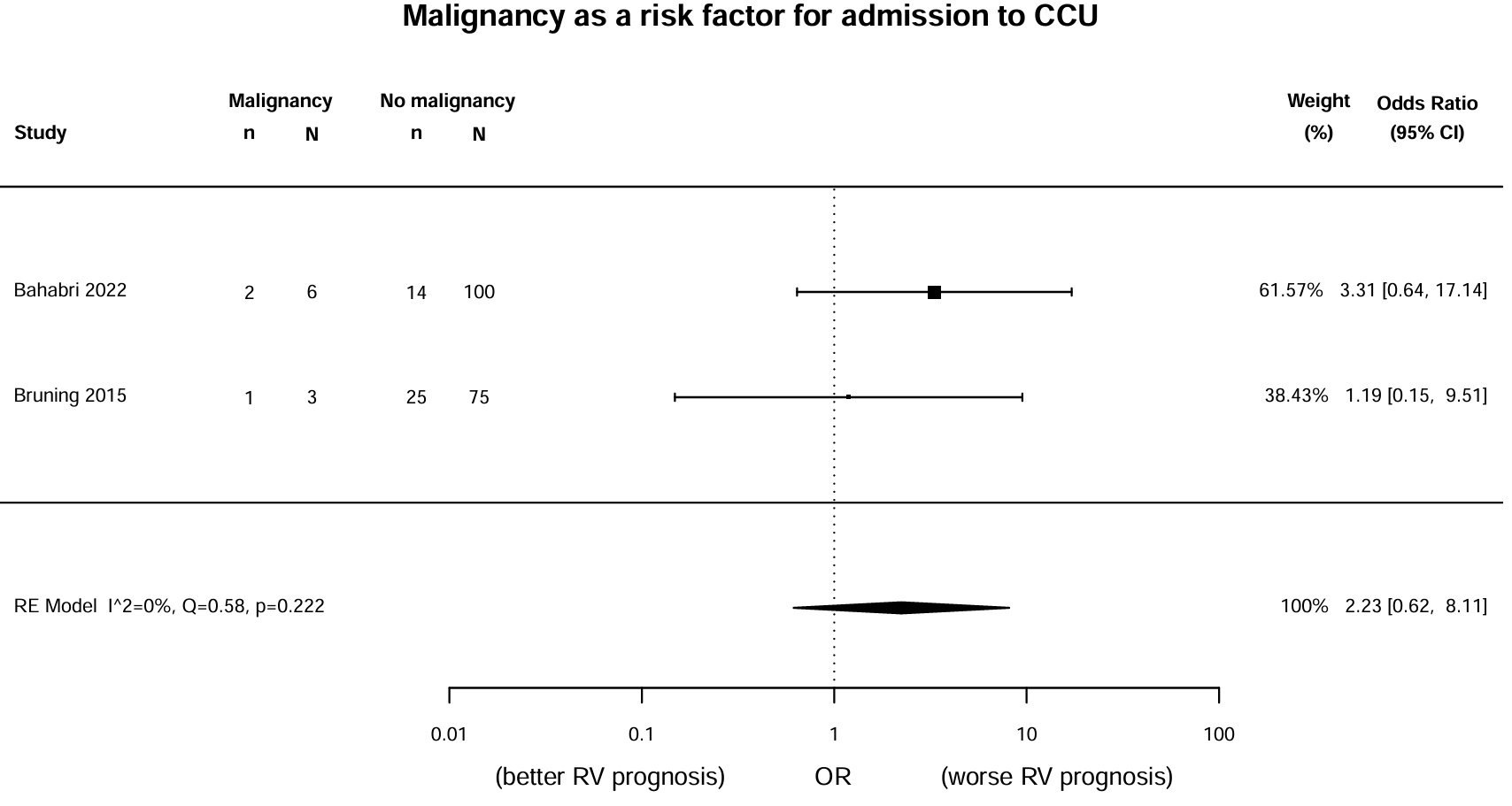


# Figure S11. Malignancy as a risk factor for mortality.


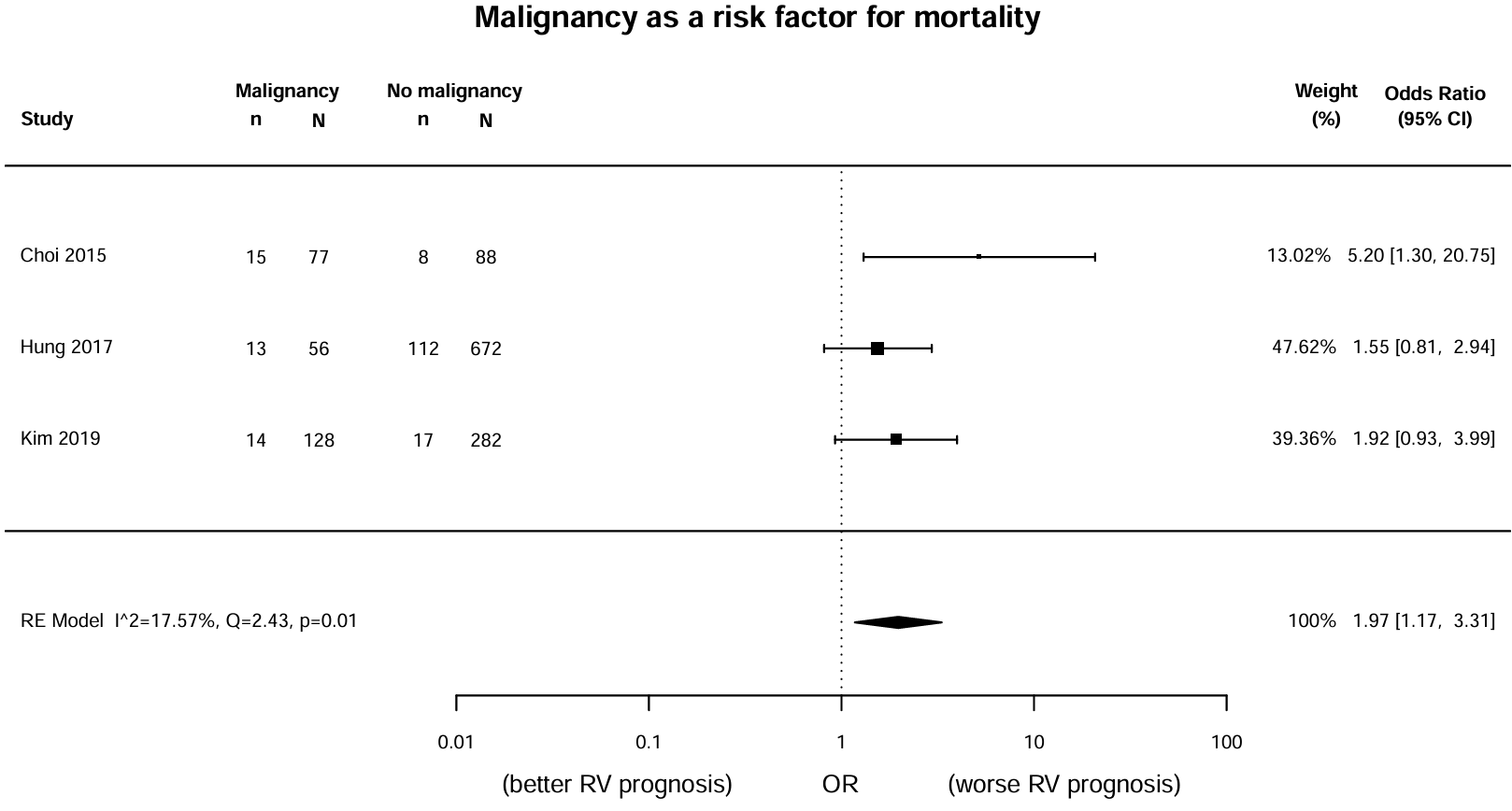


# Figure S12. Pulmonary comorbidity as a risk factor for lower respiratory tract infection.


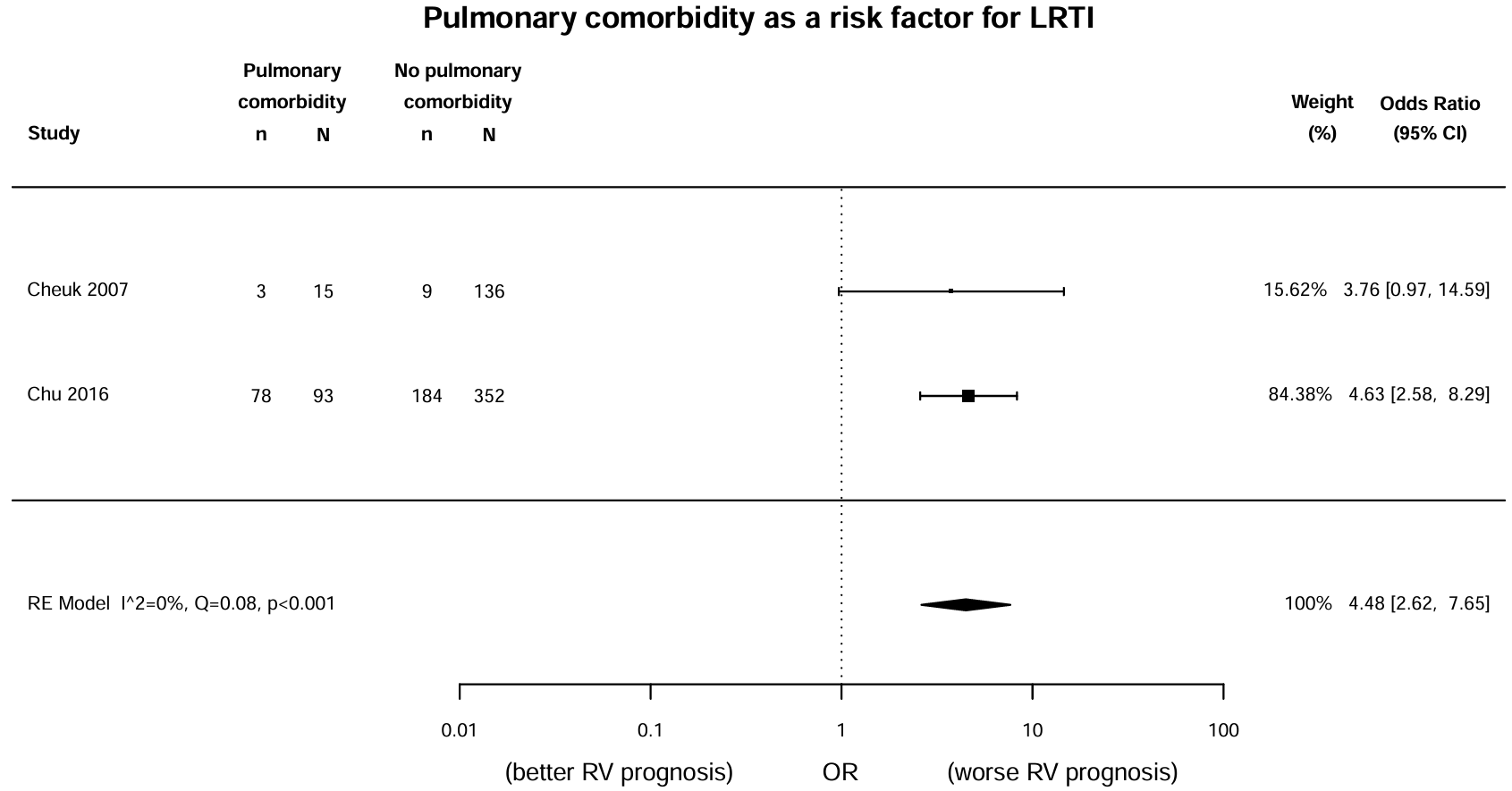


# Figure S13. Stroke as a risk factor for mortality.


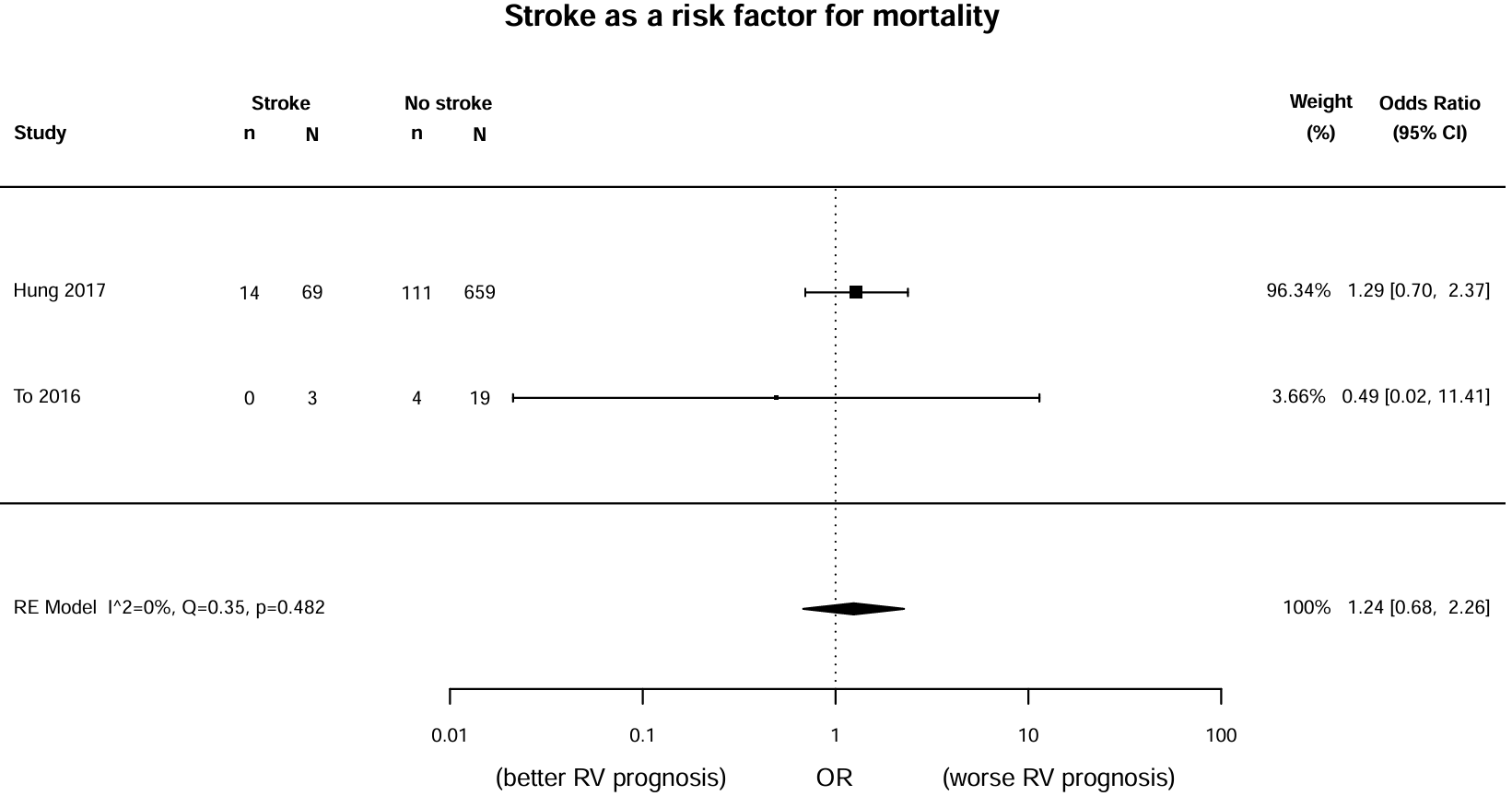

Supplement: Supplementary file 1 — Table S1: Completed PRISMA 2020 checklist. Table S2: CENTRAL search strategy from inception to April 4, 2024. Table S3: EMBASE search strategy from inception to April 4, 2024. Table S4: MEDLINE search strategy from inception to April 4, 2024. Table S5: Risk factor–outcome combinations not amenable to meta‐analysis. Table S6: Newcastle–Ottawa Scale risk of bias assessments for the included cohort studies (n = 25). Table S7: Newcastle–Ottawa Scale risk of bias assessments for the included case‐control study (n = 1). Table S8: Newcastle–Ottawa Scale risk of bias assessments for the included cross‐sectional studies (n = 3). Result S1. Citations of excluded full‐text studies. Figure S1: Age < 1 year as a risk factor for lower respiratory tract infection. Figure S2: Age ≥ 18 years as a risk factor for mortality. Figure S3: Male sex as a risk factor for admission to a critical care unit. Figure S4: Male sex as a risk factor for lower respiratory tract infection. Figure S5: Premature birth as a risk factor for lower respiratory tract infection. Figure S6: Smoking as a risk factor for lower respiratory tract infection. Figure S7: Forest plot for any comorbidity as a risk factor for admission to a critical care unit. Figure S8: Forest plot for any comorbidity as a risk factor for lower respiratory tract infection. Figure S9: Forest plot for diabetes mellitus as a risk factor for mortality. Figure S10: Malignancy as a risk factor for admission to a critical care unit. Figure S11: Malignancy as a risk factor for mortality. Figure S12: Pulmonary comorbidity as a risk factor for lower respiratory tract infection. Figure S13: Stroke as a risk factor for mortality. [file IRV-20-e70251-s001.docx]
